# Cingulin and paracingulin tether myosins-2 to junctions to mechanoregulate the plasma membrane

**DOI:** 10.1083/jcb.202208065

**Published:** 2023-05-19

**Authors:** Florian Rouaud, Wenmao Huang, Arielle Flinois, Kunalika Jain, Ekaterina Vasileva, Thomas Di Mattia, Marine Mauperin, David A.D. Parry, Vera Dugina, Christine Chaponnier, Isabelle Méan, Sylvie Montessuit, Annick Mutero-Maeda, Jie Yan, Sandra Citi

**Affiliations:** 1 Department of Molecular and Cellular Biology, Faculty of Sciences, University of Geneva, Geneva, Switzerland; 2 Department of Physics, National University of Singapore, Singapore, Singapore; 3 Mechanobiology Institute, National University of Singapore, Singapore, Singapore; 4 School of Natural Sciences, Massey University, Palmerston North, New Zealand; 5 Belozersky Institute of Physico-Chemical Biology, Moscow State University, Moscow, Russia; 6 Department of Pathology and Immunology, Faculty of Medicine, University of Geneva, Geneva, Switzerland

## Abstract

The mechanisms that regulate the spatial sorting of nonmuscle myosins-2 (NM2) isoforms and couple them mechanically to the plasma membrane are unclear. Here we show that the cytoplasmic junctional proteins cingulin (CGN) and paracingulin (CGNL1) interact directly with NM2s through their C-terminal coiled-coil sequences. CGN binds strongly to NM2B, and CGNL1 to NM2A and NM2B. Knockout (KO), exogenous expression, and rescue experiments with WT and mutant proteins show that the NM2-binding region of CGN is required for the junctional accumulation of NM2B, ZO-1, ZO-3, and phalloidin-labeled actin filaments, and for the maintenance of tight junction membrane tortuosity and apical membrane stiffness. CGNL1 expression promotes the junctional accumulation of both NM2A and NM2B and its KO results in myosin-dependent fragmentation of adherens junction complexes. These results reveal a mechanism for the junctional localization of NM2A and NM2B and indicate that, by binding to NM2s, CGN and CGNL1 mechanically couple the actomyosin cytoskeleton to junctional protein complexes to mechanoregulate the plasma membrane.

## Teaser

Rouaud et al. show that cingulin and paracingulin bind to specific myosin-2 isoforms through coiled-coil rod-mediated interactions and tether them to junctions to transmit force and modulate ZO-1 junctional accumulation, tight junction membrane tortuosity, apical membrane stiffness, and junctional architecture.

## Introduction

The apical junctional complex (AJC) of epithelial cells comprises tight junctions (TJ) and adherens junctions (AJ), and maintains cell–cell adhesion, tissue integrity, and barrier functions. The actomyosin cytoskeleton is a critical component of the AJC, since it regulates junction assembly and paracellular permeability and defines the shape and mechanical properties of the junctional and apical plasma membranes (Van Itallie and Anderson, 2014; Buckley and Turner, 2018; Tang, 2018; Citi, 2019).

Despite the essential roles of the actomyosin cytoskeleton at the AJC (Ivanov et al., 2022), the molecular mechanisms that control the spatial sorting of myosin and actin isoforms at junctions are not known. Nonmuscle myosins-2A (NM2A) and NM2B have distinct functions in cadherin clustering and adhesion, AJ integrity and dynamics, and junctional accumulation of phalloidin-labeled actin filaments (Ivanov et al., 2007; Smutny et al., 2010; Ozawa, 2018; Heuzé et al., 2019). Filaments comprising NM2A and NM2B are localized within the circumferential peri-junctional belt of bundled actin filaments on the cytoplasmic face of the AJ, but only NM2B also shows a juxta-membrane localization, associated with branched actin filaments (Efimova and Svitkina, 2018; Heuzé et al., 2019). Epithelial cells express two actin isoforms, β-actin and γ-actin, which are differentially localized at junctions and along lateral contacts (β-actin), and along the apical membrane and at AJC (γ-actin; Dugina et al., 2009; Baranwal et al., 2012). Actin filaments are connected to AJ and TJ through complexes of cytoplasmic scaffolding and adaptor proteins (reviewed in Takeichi, 2014; Rouaud et al., 2020). At TJ, ZO proteins (Zonula Occludens, ZO-1, ZO-2, ZO-3) bind to actin filaments (Fanning et al., 1998; Wittchen et al., 1999) and ZO-1 regulates the mechanics of the apical and junctional membrane. For example, ZO-1–depleted cells show decreased tortuosity of the TJ membrane and altered distribution of NM2B (Van Itallie et al., 2009; Tokuda et al., 2014), increased apical stiffness (Cartagena-Rivera et al., 2017), and altered organization of actomyosin filaments (Choi et al., 2016; Odenwald et al., 2018). However, the mechanisms through which ZO-1 organizes the actomyosin cytoskeleton and modulates membrane mechanics are not clear. Importantly, ZO-1 is a mechanosensing protein, since its conformation can be either stretched or folded, depending on actomyosin tension and heterodimerization with ZO-2 (Spadaro et al., 2017). ZO-1 stretching controls its interaction with occludin and DbpA and downstream barrier function, gene expression, and proliferation (Spadaro et al., 2017). ZO-1 stretching is also required for ZO-1 phase separation, driving TJ assembly and morphogenesis (Beutel et al., 2019; Schwayer et al., 2019; Citi, 2020). Thus, deciphering the molecular interactions involved in the mechanical coupling of actomyosin to ZO-1 is critical to understand the assembly and function of TJ. Moreover, how force is transduced from the peri-junctional belt to the AJ protein complexes is also not completely understood.

Cingulin (CGN) and paracingulin (CGNL1, JACOP) are homodimers that comprise globular head, coiled-coil rod, and globular tail domains, and are localized at TJ (CGN) and at TJ and AJ (CGNL1; Citi et al., 1988; Cordenonsi et al., 1999; Ohnishi et al., 2004; Guillemot and Citi, 2006; Pulimeno et al., 2011). CGN and CGNL1 are recruited to TJ by ZO-1, through interaction of their N-terminal ZIM (ZO-1 Interaction Motif) sequences with the C-terminal ZU5 domain of ZO-1 (D’Atri et al., 2002; Umeda et al., 2004; Pulimeno et al., 2011; Vasileva et al., 2022). CGNL1 is recruited to AJ by PLEKHA7 (Pulimeno et al., 2011). CGN binding to ZO-1 enhances the junctional accumulation and stability of ZO-1 by promoting its stretched conformation (Vasileva et al., 2022). Since the rod regions of CGN and CGNL1 show highest sequence homology to the rods of NM2s, and CGN interacts with NM2A (Cordenonsi et al., 1999; Citi et al., 2000), we hypothesized that CGN controls ZO-1 junctional accumulation by binding to NM2s and thus transmitting actomyosin-generated tension to ZO-1. Here we test this hypothesis and show that CGN and CGNL1 bind directly to NM2B and CGNL1 to NM2A in vitro, and that these interactions promote the recruitment of NM2A and NM2B to the AJC, the TJ accumulation of ZO-1, the tortuosity of the TJ membrane, the stiffness of the apical membrane, and the linear integrity of the AJ.

## Results

### CGN and CGNL1 bind directly to NM2s through interaction of their respective C-terminal coiled-coil rod domains

**Figure 1.**
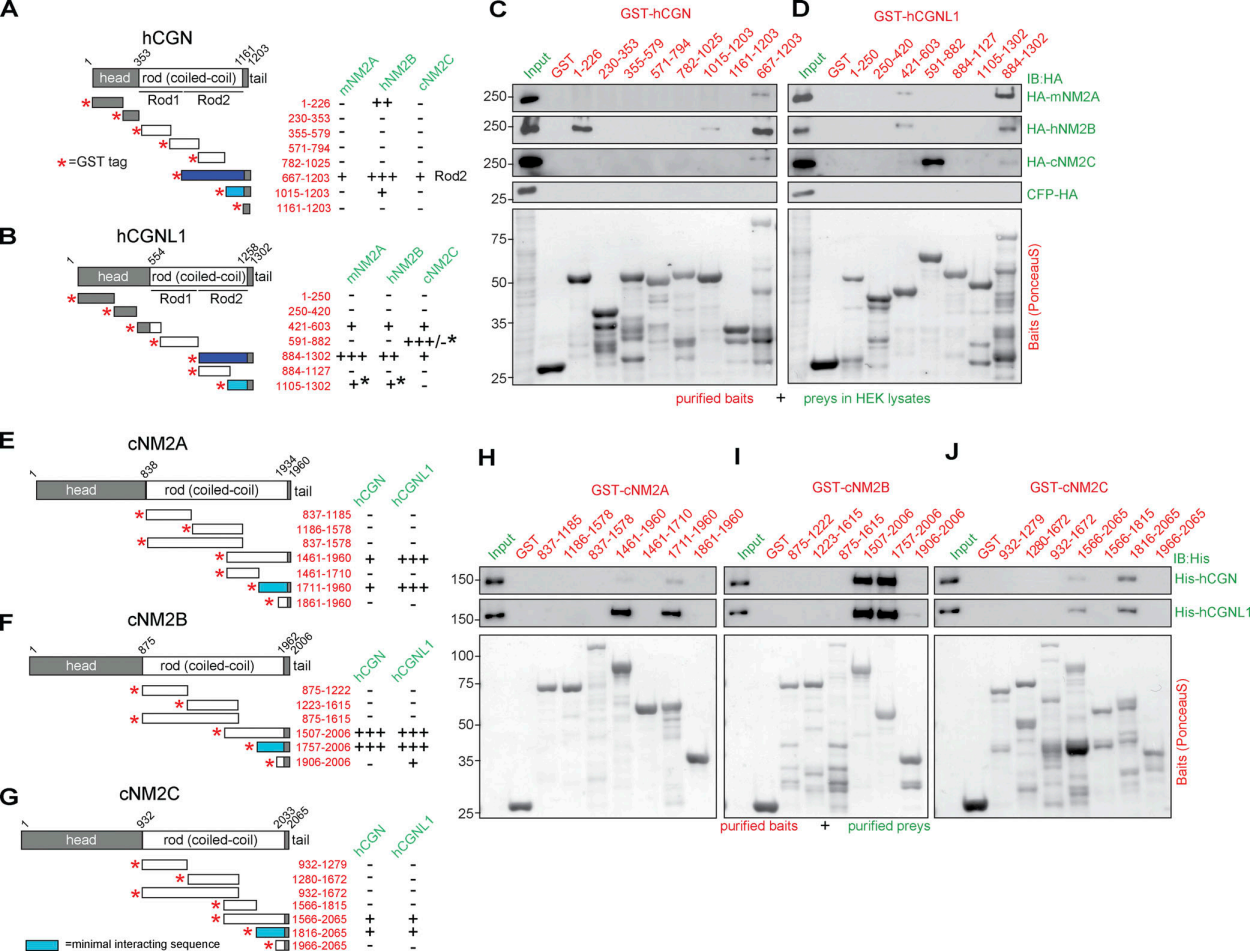
**CGN and CGNL1 interact in vitro with NM2A, NM2B, and NM2C through coiled-coil–mediated interactions. (A, B, and E–G)** Schemes (not proportional to size) of proteins and constructs (left), and summaries of interactions (right) of CGN with NM2s (A), CGNL1 with NM2s (B), NM2A with CGN and CGNL1 (E), NM2B with CGN and CGNL1 (F), and NM2C with CGN and CGNL1 (G). Red asterisks indicate N-terminal GST tag of fusion proteins. Preys are indicated in green and baits (numbers refer to amino-acid residues) in red: (−) no interaction, (+) weak interaction, (++) interaction, (+++) strong interaction (see Fig. S1 for quantifications). Dark blue–filled schemes of GST fusion constructs indicate baits of CGN and CGNL1 showing strongest interaction with NM2s. Light blue–filled schemes show the minimal interacting sequences based on pulldowns as shown in Fig. 1, C and D and H–J and Fig. S1. Black asterisks in B indicate interaction/lack of interaction based on pulldowns using purified NM2 rods (Fig. S1 D). **(C and D)** IB analysis, using anti-HA antibodies, of pulldowns using affinity purified GST-tagged CGN (C) and CGNL1 (D) baits and HA-tagged full-length NM2 preys expressed in HEK cells (HA-mNM2A, HA-hNM2B, and HA-cNM2C; prey normalization in input lanes). Bottom panels show Ponceau-red labeled baits. GST alone was the negative control bait and CFP-HA was the negative control prey. Numbers on the left (kD) indicate migration of pre-stained markers. **(H–J)** IB analysis, using anti-His antibodies, of pulldowns using affinity purified GST-tagged cNM2A (H), cNM2B (I), cNM2C (J) baits (fragments of the rod region) and His-tagged full-length CGN (top) and CGNL1 (bottom) preys purified from baculovirus-infected insect cells (Fig. S1 A). Source data are available for this figure: SourceData F1.

The domain organization of CGN and CGNL1, with head, rod, and tail domains (schemes, Fig. 1, A and B) is similar to that of NM2s (NM2A, NM2B, and NM2C, schemes in Fig. 1, E–G). The rod sequences of CGN and CGNL1 can be divided into N-terminal Rod1 and C-terminal Rod2 domains (Citi et al., 2000; Fig. 1, A and B). The Rod2 sequences are most conserved and show highest homology with the rods of NM2s (Cordenonsi et al., 1999; Citi et al., 2000; Ohnishi et al., 2004; Guillemot and Citi, 2006), suggesting potential coiled-coil–mediated interactions between the rods of CGN/CGNL1 and the rods of NM2s.

**Figure S1.**
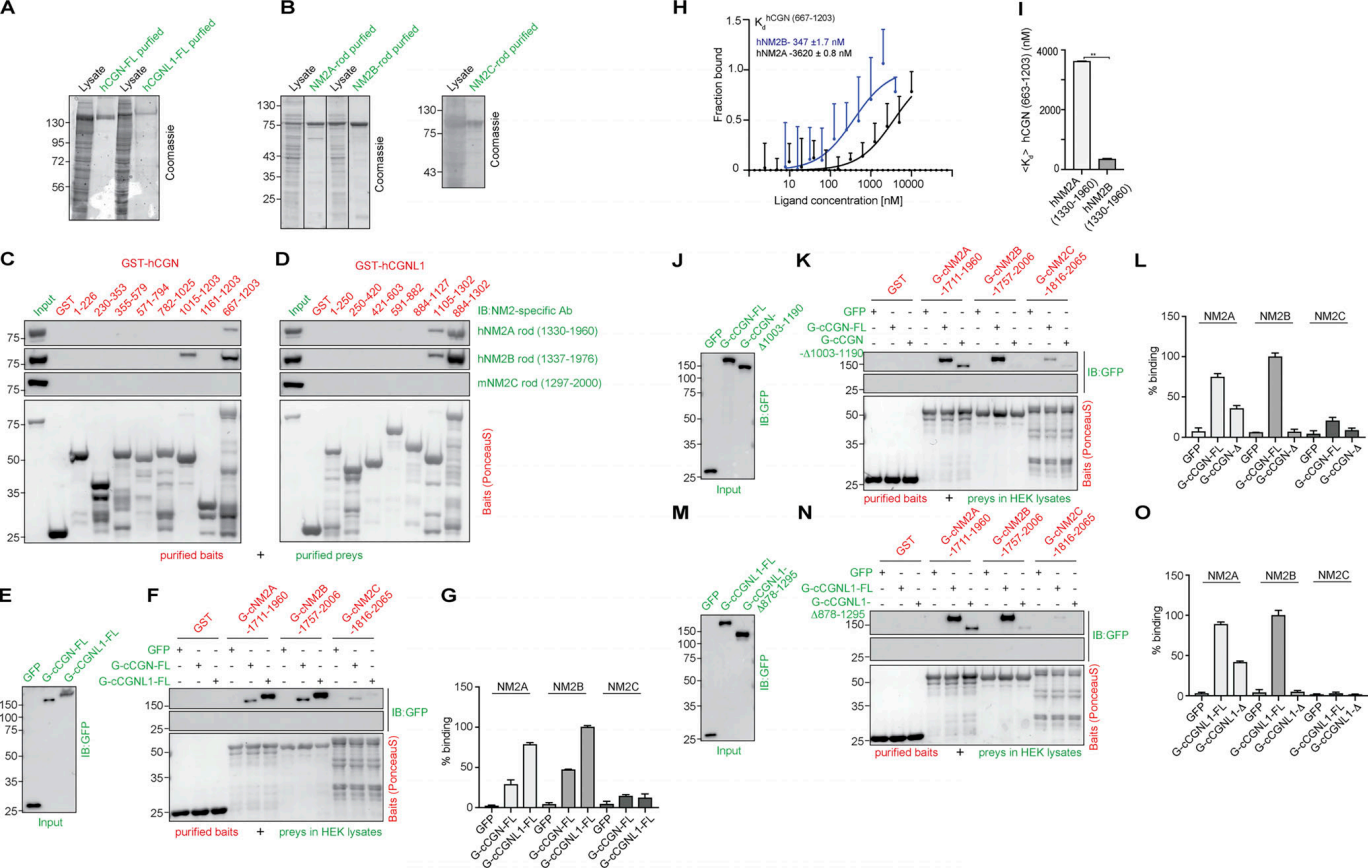
**In vitro interaction of CGN and CGNL1 with NM2A, NM2B, and NM2C.** (Related to Fig. 1.) **(A and B)** Coomassie-blue-stained SDS-PAGE gels showing purification (lysates and purified proteins) of full-length (FL) CGN and CGNL1 from baculovirus-infected cells (A) and purification of rod fragments of NM2A (1330–1960), NM2B (1337–1976), NM2C (1297–2000) from bacteria (B). Numbers on the left indicate migration of pre-stained markers. **(C and D)** IB analysis, using anti-NM2 antibodies, of pulldowns using CGN (C) and CGNL1 (D) GST-fusion protein fragments as baits (see Fig. 1, A and B, for construct schemes), and purified NM2 rod fragments (B) as preys. Baits stained with Ponceau-red are shown below IBs. Red numbers correspond to the amino acid residues comprised in each bait construct. GST alone was used as a negative control bait. **(E–G)** Comparison of the interaction of the last 250 residues of NM2s with either full-length CGN or CGNL1. IB analysis, using anti-GFP antibodies, of prey normalization (E) and GST pulldowns (F) using GST-tagged fragments of the last 250 residues of NM2s as baits, and GFP-tagged either full-length or C-terminally truncated CGN (J; FL or Δ1003-1190), or CGNL1 (M; FL or Δ878-1295) in HEK lysates as preys. **(G)** Quantification of prey binding, taking the full-length prey signal as 100% (*N* = 2 independent experiments, and data in quantifications are represented as mean ± SD). **(H and I)** MST analysis of the interaction of purified CGN Rod2 with either NM2A or NM2B purified rod. The curves (H) show fraction bound as a function of ligand concentration (nM). The labeled target was hCGN rod (667–1203, 200 nM) and the ligands were either hNM2A rod (1330–1960, black curve) or hNM2B rod (1337–1975, blue curve) with concentrations ranging from 0 to 10,000 nM. The bar graph (I) shows mean K_d_ values, and data in quantifications are represented as mean + SD (*n* = 7, Mann–Whitney test, two-tailed **P < 0.01). **(J, K, M, and N)** IB analysis, using anti-GFP antibodies, of GST pulldowns using GST fusions of C-terminal rod+tail fragments of NM2A, NM2B, and NM2C as baits and either full-length or C-terminally truncated CGN (K, prey normalization in J) or CGNL1 (N, prey normalization in M) as preys. **(L and O)** Comparative quantification of binding based on densitometric analysis of bands (*N* = 2 independent experiments and data in quantifications are represented as mean ± SD), ratioed to the strongest signal (100%) of either full-length CGN or CGNL1 interacting with the NM2B bait. The preys ending in Δ in L and O lack the C-terminal sequences indicated in J and M, respectively. Source data are available for this figure: SourceData FS1.

To test this hypothesis, we carried out GST pulldown experiments using affinity purified GST-tagged fragments as baits and preys either expressed in HEK (Human Embryonic Kidney) cell lysates (Fig. 1, C and D; and Fig. S1, E–G and J–O) or purified from either baculovirus-infected insect cells or bacteria (Fig. 1, H–J; and Fig. S1, A–D). None of the fragments comprising CGN head domain sequences interacted with full-length NM2s in HEK lysates, except for an interaction of fragment (1–226) of CGN with NM2B (Fig. 1 C), which was not observed using purified NM2 rod fragments as preys (Fig. S1 C). Similarly, a weak interaction of fragment (421–603) of CGNL1 with NM2A and NM2B (Fig. 1 D) was not observed using purified NM2 rods as preys (Fig. S1 D). This suggests that the rods of NM2s do not interact with the heads of CGN and CGNL1, but does not rule out the possibility of direct or indirect head–head interactions. Instead, the Rod2 baits of CGN and CGNL1 (667–1203 for CGN, 884–1302 for CGNL1, Fig. 1, A and B) interacted well with full-length NM2 preys (Fig. 1, C and D). Specifically, the Rod2 fragment of CGN interacted strongly with NM2B and weakly with NM2A and NM2C (Fig. 1 C), and the Rod2 fragment of CGNL1 interacted strongly with NM2A and NM2B, and weakly with NM2C (Fig. 1 D). The Rod1 of CGNL1 interacted with full-length NM2C from HEK lysates (Fig. 1 D, 591–882); however, this interaction was not detected using purified NM2C rod as a prey (Fig. S1 D), suggesting that this interaction is either indirect or mediated by the head region of NM2C. Using purified NM2 rod preys, we could also confirm that a 187-residue C-terminal fragment of CGN Rod2 was sufficient to interact with NM2B, but not with either NM2A or NM2C (Fig. 1 C and Fig. S1 C, 1015–1203). In addition, the last 197 residues of CGNL1 interact with NM2A and NM2B, but not NM2C (Fig. S1 D).

Next, we used purified full-length CGN and CGNL1 from baculovirus-infected insect cells as preys and affinity-purified GST-tagged fragments of NM2s rods as baits (Fig. 1, H–J). NM2A rod baits comprising either the last ≈500 or the last 250 residues interacted weakly with CGN but strongly with CGNL1 (NM2A 1461–1960 and 1711–1960, Fig. 1 H). Instead, the rod baits comprising the last ≈250–500 residues of NM2B interacted strongly with both CGN and CGNL1 (NM2B 1507–2006 and 1757–2006, Fig. 1 I). For NM2C, the interaction was very weak for both CGN and CGNL1 (NM2C baits 1566–2065 and 1816–2065, Fig. 1 J). Importantly, a fragment of the NM2A rod lacking the C-terminal 250 residues did not bind to purified CGN and CGNL1 (1461–1710, Fig. 1 H), and a fragment of NM2B comprising only the last 100 residues did not bind to either purified CGN or CGNL1 (1906–2006, Fig. 1 I). Together, these observations indicate that the last 250 residues of NM2s are necessary and sufficient for NM2 interaction with CGN and CGNL1. These results were confirmed using the baits comprising the last ≈250 residues of the rods of NM2A, NM2B, and NM2C, and the preys of full-length CGN and CGNL1 expressed in HEK lysates (Fig. S1, E and F, quantifications in Fig. S1 G). Moreover, microscale thermophoresis (MST) analysis showed that the Rod2 of CGN interacts with the NM2B rod with ∼10-fold higher affinity than with the NM2A rod (K_d_ of 346 nM for NM2B versus 3620 nM for NM2A, Fig. S1, H and I), in agreement with the results of the GST pulldowns (Fig. 1 C and Fig. 1, H and I). Finally, analysis of the interaction of NM2 rod fragments with either full-length or C-terminally truncated CGN and CGNL1 preys showed that deletion of either the last 187 residues of CGN or the last 417 residues of CGNL1 significantly reduced interaction with NM2A and essentially abolished interaction with NM2B (Fig. S1, J and K; and Fig. S1, M and N, quantifications in Fig. S1 L and Fig. S1 O). Together, these in vitro results show that: (1) CGN and CGNL1 interact directly with NM2s through coiled-coil–mediated interactions involving their respective C-terminal ≈200–250 residues; (2) both CGN and CGNL1 interact strongly with NM2B; (3) CGNL1 interacts strongly and CGN weakly with NM2A; (4) both CGN and CGNL1 interact weakly with NM2C.

### CGN and CGNL1 interact with NM2s in an antiparallel arrangement and the CGN C-terminal rod region perturbs the assembly of the NM2B rod

To further study the interaction between CGN/CGNL1 and NM2s, we analyzed the coiled-coil sequences of CGN and CGNL1 to predict whether parallel or antiparallel arrangement is favored (Parry et al., 1977; Table 1). Antiparallel assembly is largely favored in interactions of CGN with NM2A, NM2B, and NM2C. For CGNL1, antiparallel assembly is favored for NM2A and NM2C, whereas for NM2B, parallel and antiparallel arrangements are similarly favored. The staggers between the C-terminal ends of the two coiled-coil antiparallel molecules are ≈50 residues (CGN) and ≈72 residues (CGNL1; Table 1).

**Table 1.** Scores for intermolecular arrangements of CGN and CGNL1 with NM2A, NM2B, and NM2C

	Parallel arrangement			Antiparallel arrangement		
	Stagger	Score	Overlap	Stagger	Score	Overlap
CGN-NM2A	ND	ND	ND	421	0.2508	CC50
CGN-NM2B	440	0.2599	NS	421	0.2508	CC50
CGN-NM2C	441	0.2364	NS	421	0.2606	CC50
CGNL1-NM2A	267	NS	CN109	304	0.2373	CC72
CGNL1-NM2B	330	0.2420	CN46	304	0.2442	CC72
CGNL1-NM2C	326	NS	CN50	304	0.2716	CC72

Table shows normalized scores (>0.23) for the total number of potential apolar and ionic interactions are shown as a function of axial stagger and relative orientation between interacting molecules. Scores <0.23 or short overlaps (<32) were NS. ND indicates no score. Axial stagger is designated in terms of the number of residues in a coiled-coil conformation. CC50, for example, indicates molecules overlapping by 50 residues over the C-terminal portion of each of their coiled-coil domains, and CN indicates a C-terminal overlap of CGN or CGNL1 with an N-terminal region of the rod of NM2A, NM2B, or NM2C. Scores were calculated as described in Hulmes et al. (1973); Parry et al. (1977); Fraser et al. (1986).

**Figure 2.**
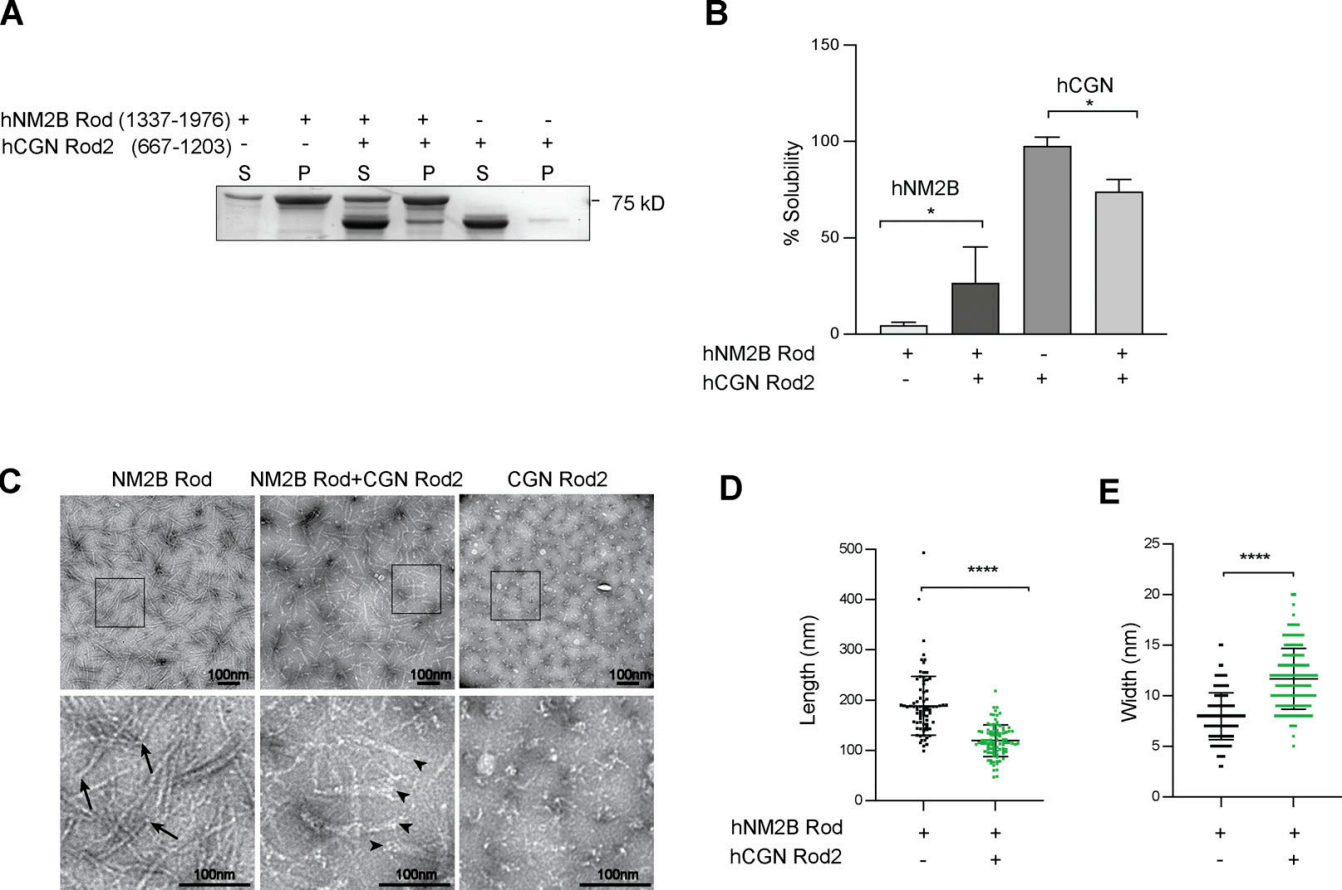
**CGN Rod2 prevents NM2B Rod filament assembly. (A and B)** SDS-PAGE analysis of supernatant (S) and pellet (P) fractions (A) and quantification of soluble supernatant fractions (B) for either purified NM2B Rod (1337–1976), or purified CGN Rod2 (667–1203), either alone or mixed together. Data in quantifications are represented as mean ± SD. Statistical significance was determined by unpaired Mann–Whitney’s test (*P ≤ 0.5; *n* = 7 experiments). **(C)** Electron microscopy analysis of negatively stained samples (top = low magnification, bottom = high magnification of insets) after dialysis of NM2B Rod alone (left panels), NM2B Rod + CGN Rod2 (middle panels), and CGN Rod2 alone (right panels). Arrows indicate compact ends of NM2B Rod filaments, arrowheads indicate frayed ends of NM2B Rod filaments in the presence of CGN Rod2. Scale bars = 100 nm. **(D and E)** Dot plots showing quantification NM2B C-terminal rod filament length and width in the absence (black dots) and in the presence (green dots) of CGN C-terminal rod fragment. Measurements were carried out on micrographs of negatively stained filaments (*n* = 200 filaments, Mann–Whitney test, two-tailed, ****P < 0.0001). Data in quantifications are represented as mean ± SD. Source data are available for this figure: SourceData F2.

We next asked whether CGN affects the ability of NM2 to form filaments. To address this question, we used a filament pelleting assay (Straussman et al., 2005; Dahan et al., 2012) and tested whether a purified C-terminal fragment of CGN rod (Rod2, 667–1203, Fig. 1 A) modulates the assembly of the NM2B Rod (1337–1976). The NM2B Rod alone was pelleted efficiently, but in the presence of the CGN Rod2, a fraction of the NM2B Rod was soluble (Fig. 2 A, quantification in Fig. 2 B). Conversely, the CGN Rod2 was soluble, but in the presence of the NM2B Rod, it was partially pelleted (Fig. 2 A, quantification in Fig. 2 B).

Negative staining electron microscopy analysis of samples after dialysis against a physiological buffer showed that the NM2B Rod formed filaments with a homogeneous size distribution and thin, tapering ends (arrows in magnified insets, Fig. 2 C, left). However, in the presence of the CGN Rod2, the NM2B Rod filaments showed heterogeneous shapes and splayed ends (arrowheads in magnified insets, Fig. 2 C, middle) and were shorter and wider (quantifications, Fig. 2, D and E). No filamentous structures could be detected after dialysis of the CGN Rod2 alone (Fig. 2 C, right), in agreement with the pelleting assay. Together, these observations confirm that CGN Rod2 does not assemble into filaments and suggest that strong binding of CGN to NM2B can perturb its filament assembly, and in turn NM2B rod filaments can trap the CGN Rod2 domain.

### CGN knockout (KO) causes decreased NM2B accumulation at junctions, depending on the CGN NM2-binding region (NM2BR)

**Figure 3.**
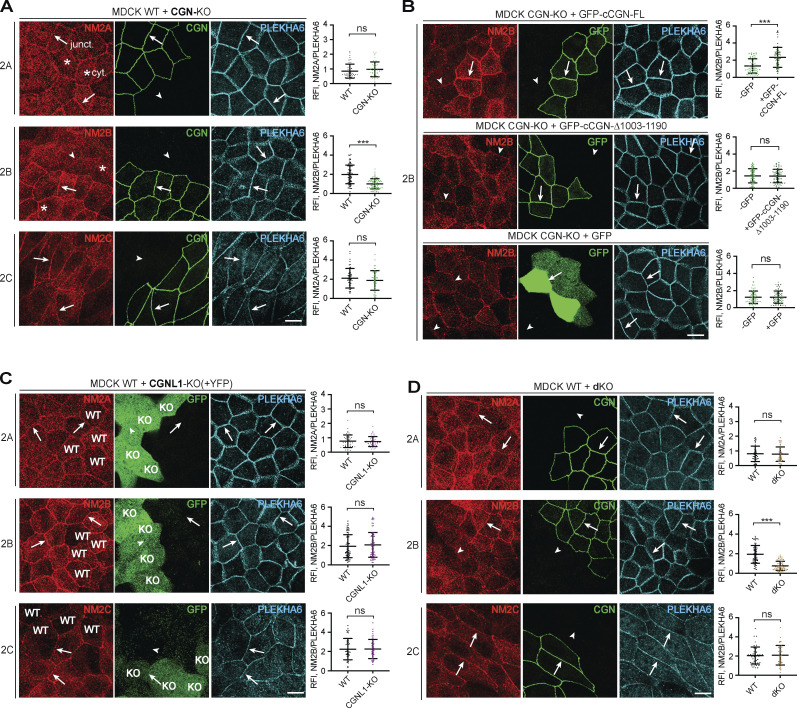
**The junctional accumulation of endogenous NM2B requires CGN and its NM2-binding region. (A, C, and D)** IF microscopy analysis (left) and quantifications of junctional labeling relative fluorescence intensity (right) of NM2A (top panels), NM2B (middle panels), and NM2C (bottom panels) in mixed cultures of WT+CGN-KO MDCK cells (A), mixed WT+CGNL1-KO MDCK cells (C; CGNL1-KO labeled by YFP), and mixed WT+CGN-CGNL1-double-KO (dKO) MDCK cells (D). **(B)** IF microscopy analysis (left) and junctional labeling relative fluorescence intensity (right) of the localization of NM2B in CGN-KO MDCK cells rescued with either full-length GFP-tagged canis CGN (cCGN, top panels), or C-terminally truncated GFP-tagged cCGN (middle panels, CGN-Δ1003-1190) or by GFP-myc alone (bottom panels, negative control). **(A–D)** Arrows and arrowheads show normal and decreased/undetected junctional labeling, respectively. Quantification of relative fluorescent intensity (RFI) shows the ratio between the junctional staining of NM2A, NM2B, and NM2C versus the junctional marker PLEKHA6 (*n* = 72 for NM2B and *n* = 48 for NM2A and NM2C junctions) from three independent experiments for NM2A/2B and two experiments for NM2C. Data in quantifications are represented as mean ± SD. Statistical significance was determined by unpaired Mann–Whitney’s test. ***P ≤ 0.001. Scale bars = 10 μm.

**Figure S2.**
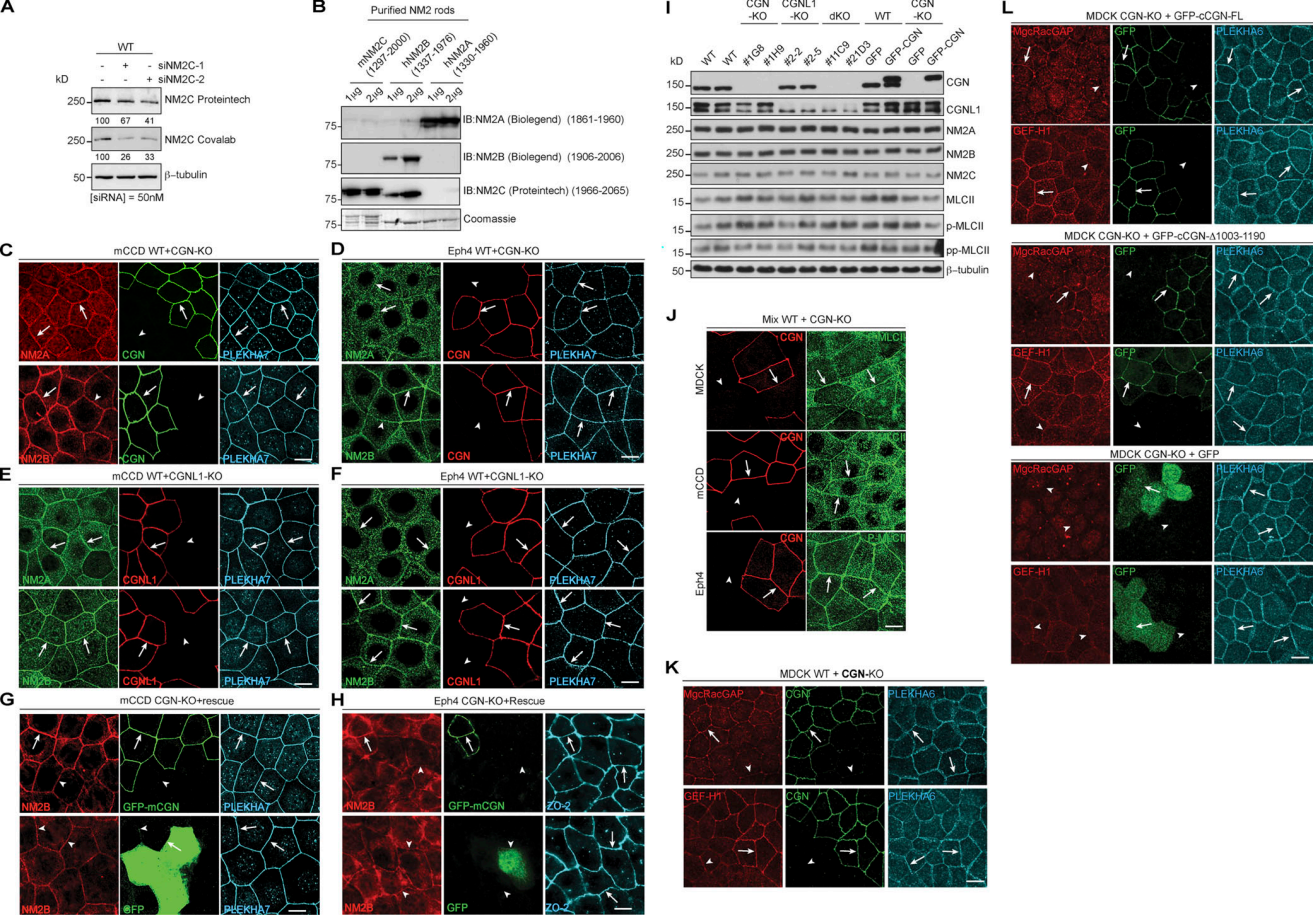
**Specificity of anti-NM2 antibodies and role of CGN in the regulation of NM2s, MgcRacGAP, and GEF-H1 in cells.** (Related to Fig. 3.) **(A and B)** Validation of specificity of the indicated antibodies against NM2C (see also Materials and methods and Table S1) by IB after siRNA-mediated depletion of NM2C in MDCK cells (A), and using purified recombinant fragments of NM2s (B). The minimal NM2 sequences recognized by the antibodies, determined by IB analysis of NM2 rod fragments (not shown), is indicated in B. Note that Proteintech anti-NM2C cross-reacts with NM2B. **(C–F)** IF microscopy localization of NM2A (top) and NM2B (bottom) in mixed cultures of WT and either CGN-KO (C and D) or CGNL1-KO (E and F) mCCD (C and E) and Eph4 (D and F) cells. Scale bars, 10 μm. **(G and H)** IF microscopy localization of NM2B in CGN-KO mCCD (G) and Eph4 (H) cells rescued either with GFP-tagged full-length CGN (top) or with GFP alone (bottom). PLEKHA7 was used as an internal reference for AJC. Scale bars, 10 μm. **(I)** IB analysis of lysates of MDCK WT, KO, and rescue lines, using the indicated antibodies. IB of CGN or CGNL1, and β-tubulin were used as line phenotype and loading controls, respectively. Numbers on the left indicate migration of prestained markers. **(J)** IF microscopy localization of phosphorylated myosin light chain (P-MLCII, green) in mixed cultures of WT+CGN-KO cells. Arrows = normal labeling, arrowheads = reduced/undetectable labeling. Scale bars, 10 μm. **(K)** IF microscopy localization of MgcRacGAP (top) and GEF-H1 (bottom) in mixed cultures of WT and CGN-KO MDCK cells. Scale bars, 10 μm. **(L)** IF microscopy localization of MgcRacGAP (top) and GEF-H1 (bottom) in CGN-KO MDCK cells rescued with either full-length (FL) GFP-tagged canis CGN (cCGN, top panels), or C-terminally truncated GFP-tagged cCGN (middle panels, CGN-Δ1003-1190) or by GFP-myc alone (bottom panels, negative control). Scale bars, 10 μm. Source data are available for this figure: SourceData FS2.

Next, we asked whether CGN and CGNL1 affect the junctional recruitment of NM2s in cells. We first examined by immunofluorescence (IF) microscopy the localization of endogenous NM2s in epithelial cells (MDCK, mouse collecting duct cells [mCCD], and Eph4) lacking either CGN or CGNL1, or both (CGN-KO, CGNL1-KO, double-KO; Vasileva et al., 2022). Junctional labeling was quantified using PLEKHA6 as an internal reference (Fig. 3, graphs on the right of IF panels). We used anti-NM2A/B/C antibodies whose specificity was established in previous studies (see Materials and methods), and we determined by immunoblotting (IB) of NM2C-depleted cells the specificity of two additional anti-NM2C antibodies (Materials and methods and Fig. S2, A and B). In WT MDCK cells, NM2s showed cytoplasmic/cortical (asterisks, cyt., Fig. 3 A) and junctional labeling (arrows, junct., Fig. 3 A). In CGN-KO MDCK cells, the intensity and localization of NM2A and NM2C signal were indistinguishable from that of the adjacent WT cells (arrows, Fig. 3 A, top and bottom panels). Instead, junctional labeling for NM2B was significantly decreased in CGN-KO cells, compared to WT (arrowhead, Fig. 3 A, middle panels). A weak residual junctional labeling for NM2B was still observed in CGN-KO cells (arrowheads in Fig. 3 A, middle panel), which could be due to NM2B copolymerized with and stabilized by peri-junctional NM2A filaments. Expression of GFP-tagged full-length CGN in CGN-KO MDCK cells rescued normal NM2B junctional labeling (Fig. 3 B, top), whereas expression of either GFP-CGN lacking the last 187 residues (NM2BR; Fig. 3 B, middle), or GFP alone (Fig. 3 B, bottom) did not. Thus, the last 187 residues of CGN, which are sufficient for binding to NM2B (Fig. S1, J–L), are also necessary for robust accumulation of NM2B at junctions.

CGNL1-KO MDCK cells were identified by exogenous expression of YFP (Fig. 3 C, green, KO), because of low levels of endogenous CGNL1 in WT cells. In CGNL1-KO cells, the cytoplasmic and junctional labeling for NM2A, NM2B, and NM2C was indistinguishable from that of WT cells (arrows, Fig. 3 C, bottom). In double CGN/CGNL1-KO cells, the localization of NM2A, NM2B, and NM2C was identical to that of CGN-KO cells, with only a reduction in NM2B junctional labeling (arrowhead, Fig. 3 D, middle), and no effect on either NM2A or NM2C (arrows, Fig. 3 D, top and bottom).

Similar results were obtained using mCCD and mouse mammary epithelial cells (Eph4; Fig. S2, C–H). Junctional labeling for NM2B but not NM2A was decreased in CGN-KO cells (arrowheads and arrows, Fig. S2, C and D) neither was affected in CGNL1-KO cells (arrows, Fig. S2, E and F) and junctional NM2B was rescued by expression of full-length CGN in CGN-KO cells (arrows, Fig. S2, G–H, top panels). The protein levels of NM2A, NM2B, and NM2C heavy chains, total myosin light chains, single-phosphorylated, and double-phosphorylated myosin light chains were similar in WT, single-KO, rescue, and double-KO MDCK cells (Fig. S2 I). The junctional signal for phosphorylated myosin light chains was similar in WT and CGN-KO cells (Fig. S2 J), indicating that reduced NM2B localization at junctions of CGN-KO cells does not correlate with either reduced NM2B levels, or up-regulation of NM2A and NM2C levels, or altered global and junctional myosin light-chain phosphorylation. Moreover, the CGN mutant lacking the NM2BR was normally localized at junctions and rescued, like the WT construct, the localization of MgcRacGAP and GEF-H1 at junctions (Fig. S2, K and L), indicating that the loss of the NM2BR has no effect on the conformation and known functions of the head and Rod1 regions of CGN.

In summary, analysis of KO cells indicated that the NM2BR of CGN is specifically required for the recruitment of NM2B to junctions, whereas KO of CGNL1 does not result in detectable loss of junctional NM2s in MDCK, mCCD, and Eph4 cells.

### Expression of CGN and CGNL1 in double-KO MDCK cells promotes the accumulation of NM2B (CGN), and NM2A and NM2B (CGNL1) at junctions

**Figure S3.**
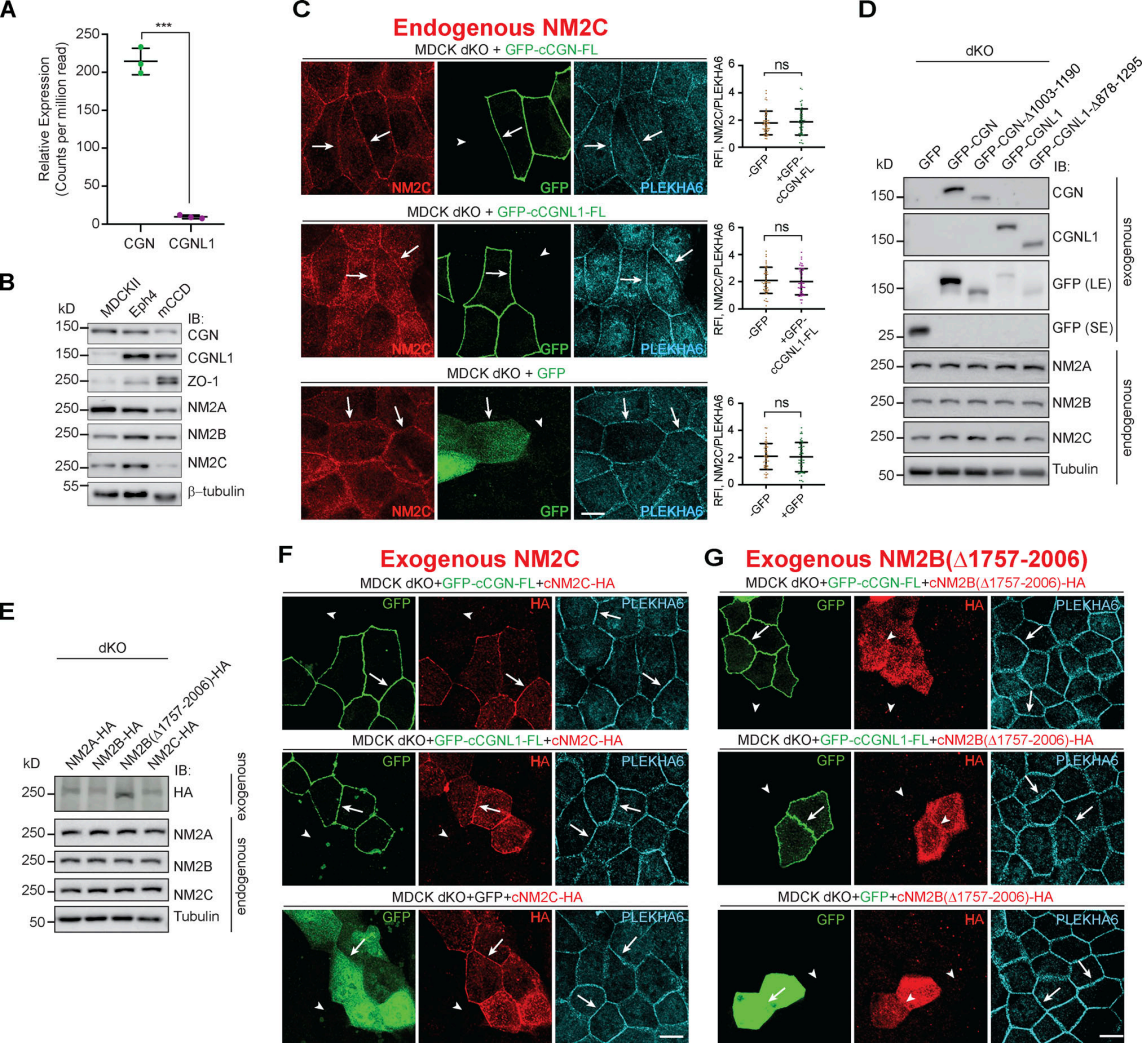
**The junctional accumulation of NM2C is not promoted by CGN and CGNL1.** (Related to Fig. 4.)** (A)** Levels of CGN and CGNL1 expression by RNASeq in MDCKII cells (*n* = 3). Data in quantifications are represented as mean ± SD, and statistical significance was determined by unpaired Mann–Whitney’s test. ***P ≤ 0.001. **(B)** IB analysis of lysates of MDCK cells, Eph4 cells, and mCCD cells using different antibodies against CGN, CGNL1, ZO-1, NM2A, NM2B, and NM2C. Antibody against β-tubulin was used as loading control. **(C)** IF microscopy analysis of the localization of endogenous NM2C in CGN-CGNL1 double-KO (dKO) MDCK cells, upon overexpression of GFP-tagged constructs of full-length (FL) CGN (top panel), full-length CGNL1 (middle panel), or GFP alone (bottom panel). Cells that overexpress exogenous constructs are shown in the same field as non-transfected cells, for direct comparison. Data in quantifications are represented as mean ± SD and statistical significance was determined by unpaired Mann–Whitney’s test (*n* = 48 junctions of two independent experiment). **(D and E)** IB analysis of lysates of MDCK dKO cells overexpressed CGN or CGNL1 or mutant tagged with GFP (D), or overexpressed NM2A or NM2B or NM2C or mutant (E), using the indicated antibodies. β-tubulin was used as loading control. Numbers on the left indicated migration of prestained markers. **(F and G)** IF microscopy localization of exogenous, full-length HA-tagged NM2A (F) and NM2B lacking the CGN/CGNL1 binding region (NM2BΔ1757-2006; G) in CGN/CGNL1 double-KO cells expressing either GFP-tagged full-length CGN (top panels), or GFP-tagged full-length CGNL1 (middle panels), or GFP alone (bottom panels). Arrows indicate junctional localization and arrowheads reduced/undetected junctional localization. PLEKHA6 labeling is used as a reference junctional labeling. Scale bars, 10 μm. Source data are available for this figure: SourceData FS3.

**Figure 4.**
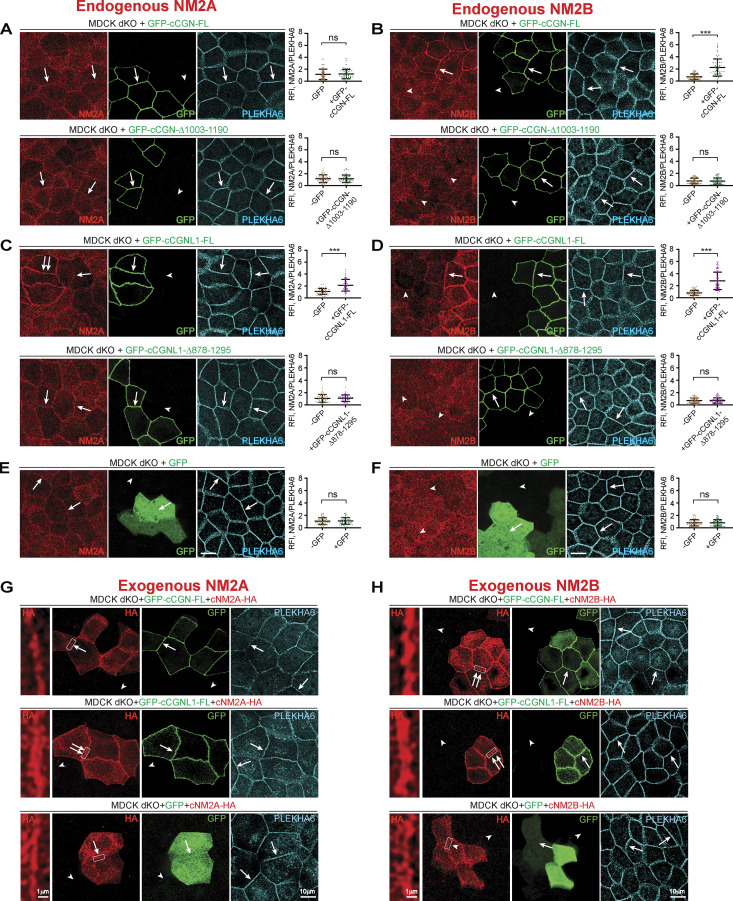
**Expression of CGN and CGNL1 promotes the junctional accumulation of NM2B and NM2A and NM2B, respectively. (A–F)** IF microscopy analysis of the localization of endogenous NM2A (A, C, and E), NM2B (B, D, and F) in CGN-CGNL1 double-KO (dKO) MDCK cells, upon expression of GFP-tagged constructs of full-length (FL) CGN (A and B, top), C-terminally truncated CGN (A and B, bottom), FL CGNL1 (C and D, top), C-terminally truncated CGNL1 (C and D, bottom), or GFP alone (E and F). NM2 labeling in neighboring transfected and non-transfected cells is directly compared and quantified using PLEKHA6 as a junctional reference marker for quantifications on the right of the IF panels. Arrows indicate junctional localization, arrowheads indicate reduced or undetectable labeling, double arrows indicate increased junctional labeling. Data in quantifications are represented as mean ± SD (NM2A *n* = 54, NM2B *n* = 66 junctions) from three independent experiments. Statistical significance was determined by unpaired Mann–Whitney’s test. ***P ≤ 0.001. **(G and H)** IF microscopy analysis of the localization of exogenous HA-tagged FL NM2A (G) and NM2B (H) in double-KO MDCK cells, upon co-expression of GFP-tagged constructs of FL CGN (top), CGNL1 (middle), and GFP (bottom). Magnified insets on the left show details of NM2 localization (red channel). Scale bars = 10 μm (main panels, A–H) and 1 μm (magnified insets, G and H).

Since MDCK cells express low levels of CGNL1 (Vasileva et al., 2017; Fig. S3, A and B), we next analyzed the role of CGN and CGNL1 in the junctional accumulation of NM2s by expressing either CGN or CGNL1 in the background of CGN/CGNL1 double-KO MDCK cells (Fig. 4 and Fig. S3, C and D). We used either full-length constructs of CGN and CGNL1, or constructs lacking their respective C-terminal NM2BR.

The junctional accumulation of endogenous NM2A was not increased by expression of either full-length or C-terminally truncated constructs of CGN (arrows, Fig. 4 A). In contrast, junctional NM2A was increased by expression of full-length CGNL1, but not C-terminally truncated CGNL1 (double arrows and arrow, Fig. 4 C). Full-length but not C-terminally truncated CGN rescued the decreased junctional accumulation of endogenous NM2B in double-KO cells (arrow and arrowheads, Fig. 4 B), confirming that the last 187 residues of CGN are required to recruit NM2B to junctions. Expression of full-length CGNL1 also rescued the decreased junctional NM2B (arrow, Fig. 4 D, top), whereas either the C-terminally truncated mutant of CGNL1 (arrowheads, Fig. 4 D, bottom) or GFP alone (Fig. 4, E and F) neither increased NM2A nor rescued NM2B. For endogenous NM2C, no significant increase in junction associated labeling was detected in cells expressing either full-length CGN or full-length CGNL1 or GFP (arrows in Fig. S3 C).

Finally, we exogenously overexpressed either GFP-tagged CGN or CGNL1 in combination with HA-tagged NM2 heavy chains in the background of double-KO cells (Fig. 4, G and H; and Fig. S3, D–G). The expression levels of endogenous NM2s were not affected by expression of CGN, CGNL1, and NM2s constructs (Fig. S3, D and E). When co-expressed with GFP alone in double-KO cells, both full-length NM2A (arrow, Fig. 4 G, bottom panels) and NM2C (arrow, Fig. S3 F, bottom panels) were detectable at cell–cell junctions, indicating that NM2A and NM2C can be targeted to junctions independently of CGN and CGNL1. However, expression of CGNL1 but not of CGN increased junctional labeling for NM2A (Fig. 4 G, middle panel). NM2B labeling in double-KO cells expressing GFP was diffusely peri-junctional (arrowheads, Fig. 4 H bottom), possibly due to copolymerization with NM2A. However, expression of either CGN or CGNL1 increased junctional NM2B labeling, and rendered it sharply accumulated at junctions (double arrows, Fig. 4 H). In contrast, junctional labeling for NM2C was not increased by expression of either CGN or CGNL1 (Fig. S3 F, top and middle). When we overexpressed either CGN or CGNL1 together with a C-terminally truncated NM2B, which cannot neither interact with CGN and CGNL1 nor, presumably, form homo-polymeric or hetero-polymeric filaments, NM2B failed to sharply accumulate at junctions (arrowheads, Fig. S3 G).

In summary, rescue/expression experiments confirmed the role of CGN in sorting NM2B to junctions and revealed a role for CGNL1 in promoting the junctional accumulation of both NM2A and NM2B, but not NM2C.

### Architecture of the junction-myosin interface: CGN and CGNL1 connect ZO-1 and PLEKHA7, respectively, to NM2B

**Figure S4.**
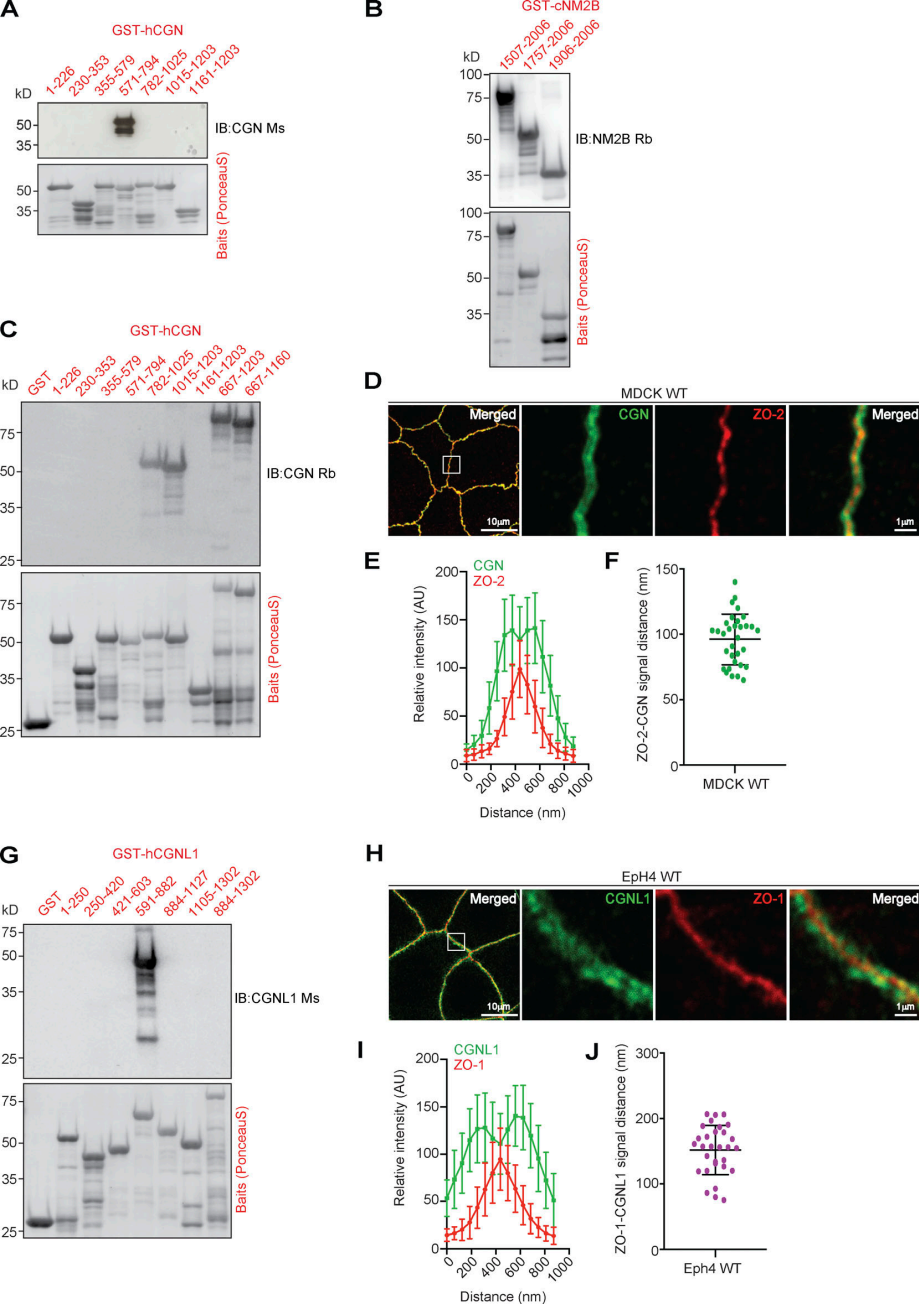
**Mapping of antibody-binding regions and calculated distances between CGN and CGNL1 and ZO proteins.** (Related to Fig. 5.) **(A–C and G)** IB analysis (top panels) and Ponceau-S labeling (bottom panels) of bacterially expressed GST-fusion protein fragments of CGN (A and C), NM2B (B), and CGNL1 (G) using the indicated antibodies, to map the antibody epitopes. **(D–F and H–J)** IF microscopy analysis of the localization of endogenous CGN in MDCK WT cells (D) and CGNL1 in EpH4 WT cells (H). High magnification panels correspond to highlighted white box in low magnification micrograph; scale bars = 10 μm (low magnification) and 1 μm (high magnification) for (D) and (H). **(E, F, I, and J)** Linescan analysis of signal distribution (E and I) and box plots of distances of CGN signal from ZO-2 signal (F) and CGNL1 signal from ZO-1 signal (J). For E, F, I, and J, *n* = 24 from two independent experiments, and data in quantifications are represented as mean ± SD. Source data are available for this figure: SourceData FS4.

**Figure 5.**
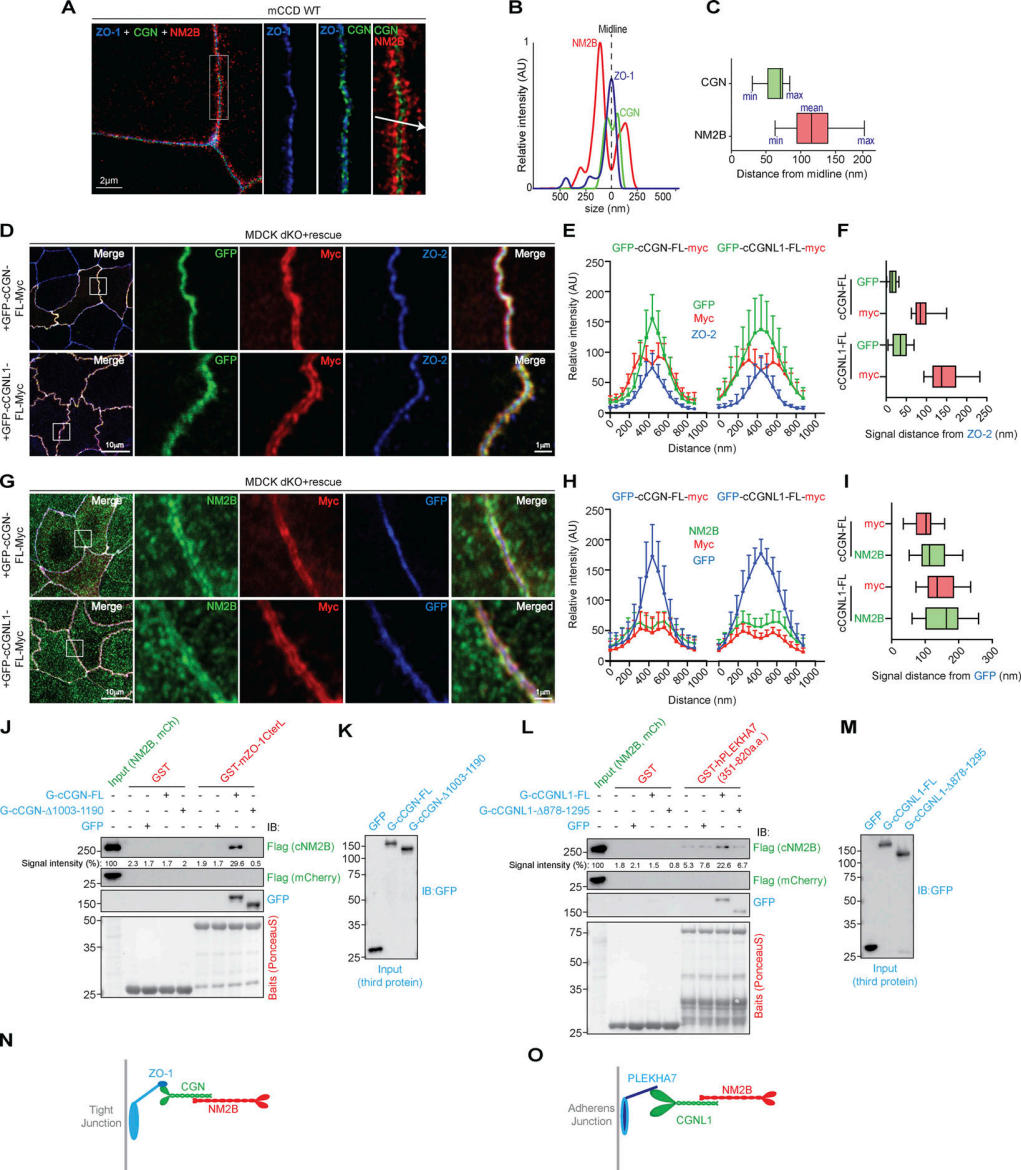
**CGN and CGNL1 are positioned between TJ and AJ scaffolding complexes, respectively, and NM2s. (A)** Super-resolution STED analysis of ZO-1 (blue), CGN (green), and NM2B (red) in mCCD WT cells (merged in left panel, scale bar = 2 µm), and magnified (2.5×) images of rectangular inset detailing labeling for ZO-1 (blue), ZO-1+CGN, and merge image with arrow indicating linescan direction. **(B)** Linescan analysis with fluorescence intensities of the three channels as a function of distance from midline ZO-1 labeling. **(C)** Box plots showing measured distances of CGN (green) and NM2B (red) from midline (ZO-1 labeling; nm; *n* = 25 from two independent experiments). **(D)** IF microscopy analysis of the localization of the N- and C-termini (GFP-green and myc-red tags, respectively) of exogenous CGN (upper panels in D) and CGNL1 (bottom panels in D) and endogenous ZO-2 (blue) in double-KO (dKO) MDCK cells. High magnification panels correspond to highlighted white box in low magnification micrograph; scale bars = 10 μm (low magnification) and 1 μm (high magnification). **(E and F)** Linescan analysis of signal distribution (E) and box plots of distances of GFP and myc signal from ZO-2 signal (F; *n* = 24 from two independent experiments and data in quantifications are represented as mean ± SD). **(G)** IF microscopy analysis of the localization of the N- and C-termini (GFP-blue and myc-red tags, respectively) of exogenous CGN (upper panels in G) and CGNL1 (bottom panels in G) and endogenous NM2B (green) in double-KO MDCK cells. High magnification panels correspond to highlighted white box in low magnification micrograph; scale bars = 10 μm (low magnification) and 1 μm (high magnification). **(H and I)** Linescan analysis of signal distribution (H) and box plots of distances of NM2B and myc signal from GFP signal (I; *n* = 23 from two independent experiments and data in quantifications are represented as mean ± SD). **(J–M)** IB analysis, using anti-Flag tag antibodies, of preys (either Flag-NM2B or Flag-mCherry as negative control [J and L]) and third proteins (IB with anti-GFP in J and L), in trimolecular GST pulldowns using either GST (negative control) or GST-mZO-1CTerL (J) or GST (negative control) or GST-hPLEKHA7(351–820) (L) as baits, and either GFP or GFP-tagged full length or C-terminally truncated CGN (normalization in K), or either GFP-tagged full length or C-terminally truncated CGNL1 (normalization in M) as third proteins. Numbers on the left of IBs show migration of pre-stained markers. Baits are shown in Ponceau-S labeled blots. **(N and O)** Schematic models of the spatial organization and relative positions of the ZO-1–CGN–NM2B and PLEKHA7–CGNL1–NM2B complexes at TJ and AJ, respectively. The stoichiometries of interactions between the proteins of the complexes are not known. Source data are available for this figure: SourceData F5.

To confirm that CGN and CGNL1 tether NM2s to the scaffolding complexes of TJ and AJ, we carried out super resolution (STED) and conventional IF microscopy using antibodies binding to specific regions of ZO-1, CGN, CGNL1, and NM2B (Rouaud et al., 2019 and Fig. S4, A–C and G). Analysis of intensity profiles across the junction indicated that CGN is positioned between ZO-1 and NM2B, with an intensity peak for CGN at a distance of 64.6 ± 13.8 nm and for NM2B at 117.13 ± 29.4 nm, with respect to the ZO-1 midline (Fig. 5, A–C).

To map more precisely the positions of CGN and CGNL1, we exogenously expressed in the background of double-KO cells constructs of CGN and CGNL1 with N-terminal GFP and C-terminal myc tags (Fig. 5, D–I). The N-termini of both CGN and CGNL1 were closest to ZO-2 labeling, whereas the C-termini were at a distance of ≈90 nm (90 ± 21.2 nm, *n* = 38) for CGN (Fig. 5 D top panels and profile distances in Fig. 5, E and F) and ≈150 nm (147 ± 36.4 nm, *n* = 40) for CGNL1 (Fig. 5 D bottom panels and profile distances in Fig. 5, E and F). This suggested that the CGNL1 C-terminus extends away from the junctional membrane farther than CGN. The relative positions of CGN and ZO-2 were confirmed using antibodies that bind to the C-terminus of endogenous CGN, indicating that the C-terminal half of CGN is ≈ 100 nm distal with respect to ZO-2 (Fig. S4, C–F, 96 ± 19.3 nm distance), consistent with the length of the CGN rod (≈130 nm; Citi et al., 1988). For CGNL1, an antibody that binds to the N-terminal portion of the rod gives an IF signal with two peaks with respect to the ZO-1 labeled central midline (Fig. S4, H and I) and a distance of ≈150 nm (151 ± 37.8 nm; Fig. S4, G–J). Labeling for endogenous NM2B spatially mapped near the C-termini of both CGN and CGNL1 (Fig. 5, G–I). Together, these results indicate that CGN and CGNL1 are localized in an intermediate position between the submembrane junctional plaque and NM2B, with the N-termini closest to the ZO protein-containing TJ plaque and the C-termini near NM2B.

**Figure S5.**
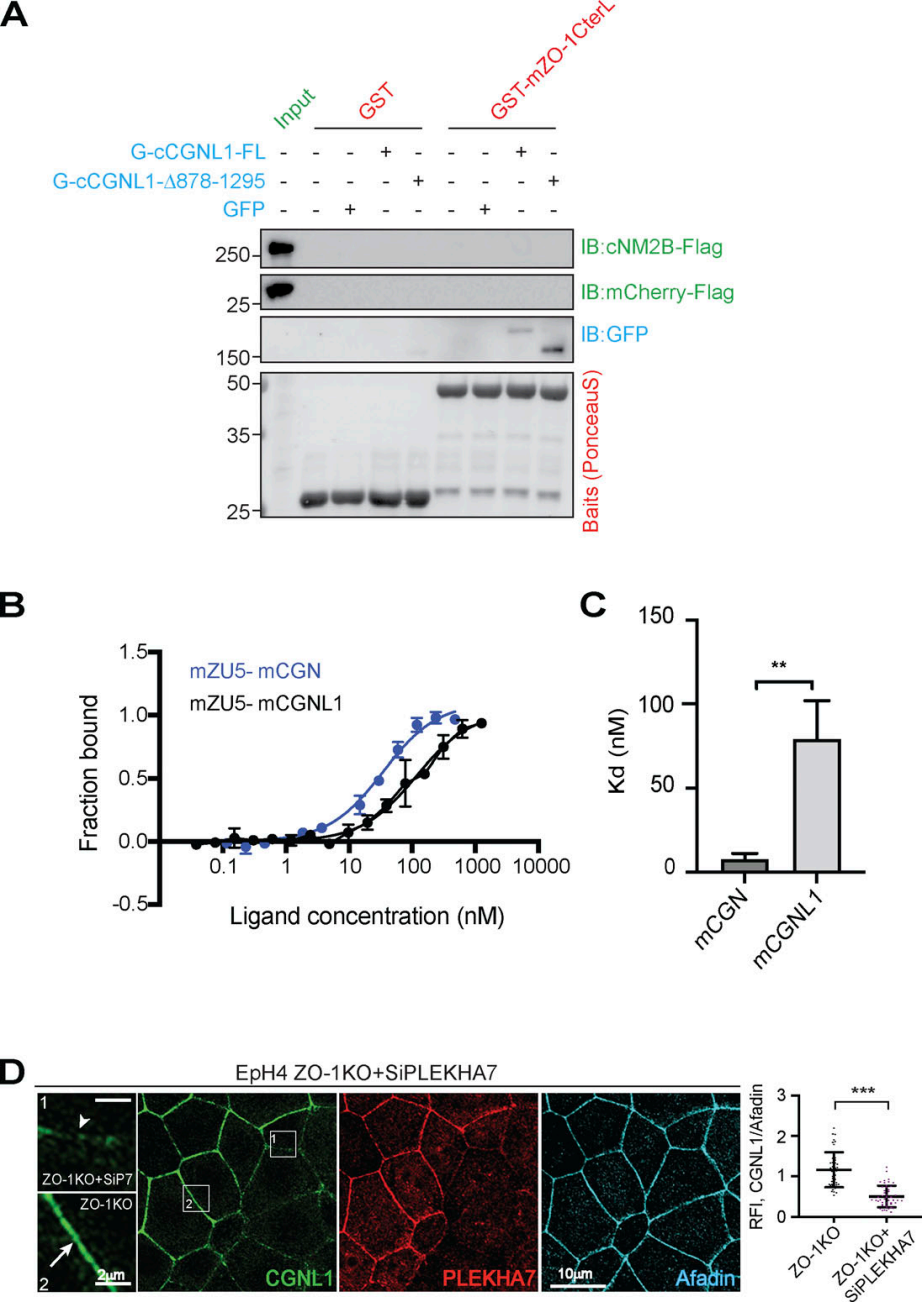
**CGNL1 does not link ZO-1 to NM2B in vitro and binds to the ZU5 domain of ZO-1 with 10-fold lower affinity than CGN.** (Related to Fig. 5.) **(A)** IB analysis of GST pulldowns using either GST alone or GST-mZO-1CterL as baits (red), and either Flag-tagged FL NM2B or Flag-tagged mCherry (negative control) as preys (green, normalized input in first lane). The third protein (blue) was either GFP-tagged FL CGNL1, or GFP-tagged CGNL1 lacking the C-terminal NM2-binding region (normalization in Fig. 5 M). NM2B and mCherry were detected by anti-Flag antibodies. Numbers on the left indicate migration of prestained markers. **(B and C)** MST measurement (B) of the dissociation constant (K_d_ [C]) for the interaction between the ZU5-containing region of mZO-1 (residues 1520–1745), and either FL CGN or FL CGNL1. The curves (B) show fraction bound as a function of ligand concentration (nM). The concentration of the target was 50 nM and the concentrations of CGN and CGNL1 ranged between 1 and 1,000 nM. The bar graph (C) shows mean K_d_ values, and data in quantifications are represented as mean + SD (*n* = 5, unpaired Mann–Whitney test [**P < 0.01]). **(D)** IF microscopy analysis of the localization of CGNL1 (green) in ZO-1-KO Eph4 cells treated with siRNA targeting PLEKHA7 (red), using afadin as a junctional reference marker for quantifications. High magnification panels correspond to highlighted white box in low magnification micrograph; scale bars = 10 μm (low magnification) and 2 μm (high magnification). Boxed areas are magnified in insets on the left, comparing PLEKHA7-undepleted and -depleted cells (arrow and arrowheads indicating normal and reduced CGNL1 labeling). For quantifications, data in plots (*n* = 48 from two independent experiments) are represented as mean ± SD (unpaired Mann–Whitney test [***P <0.001]). Source data are available for this figure: SourceData FS5.

To confirm these observations by a biochemical assay, we carried out trimolecular GST pulldowns. A ZU5-containing C-terminal fragment of ZO-1 (mZO-1CterL; Vasileva et al., 2022) was used as a bait, Flag-tagged NM2B was used as a prey, and either full-length or C-terminally truncated CGN or CGNL1, or GFP (negative control) were added as third (linker) proteins. The C-terminal fragment of ZO-1 did not bind to NM2B, unless full-length CGN was present (Fig. 5 J, third protein normalization in Fig. 5 K). However, in the presence of the C-terminally truncated CGN fragment that does not bind to NM2B, no trimolecular complex was formed (Fig. 5 J, third protein normalization in Fig. 5 K). In contrast, full-length CGNL1 failed to mediate the interaction of NM2B with the ZU5-containing ZO-1 bait (Fig. S5 A). The inability of CGNL1 to link ZO-1 to NM2B could be due to a low affinity of binding of CGNL1 to the ZU5 domain of ZO-1, as suggested by our previous studies (Vasileva et al., 2022). MST analysis confirmed that the dissociation constant (K_d_) to the ZU5-containing fragment was 7.7 ± 3.5 nM for CGN, and about 10 times higher for CGNL1 (K_d_ = 79.8 ± 25 nM; Fig. S5, B and C).

CGNL1 is recruited to the AJC not only by ZO-1 but also by PLEKHA7 (Pulimeno et al., 2011), and simultaneous depletion of ZO-1 and PLEKHA7 is required to significantly decrease CGNL1 junctional labeling (Fig. S5 D). Thus, we asked whether CGNL1 can tether NM2B to PLEKHA7 at AJ by carrying out GST pulldowns using the CGNL1-binding region of PLEKHA7 as a bait. IB analysis showed that the PLEKHA7 bait interacted weakly with NM2B and that this interaction was strongly increased in the presence of full-length CGNL1, but not truncated CGNL1, lacking the NM2-binding region (Fig. 5 L, third protein normalization Fig. 5 M).

Together, these results suggest that CGN tethers NM2B to ZO-1 at TJ (Fig. 5 N), whereas CGNL1 tethers NM2B to PLEKHA7 at AJ (Fig. 5 O).

### The accumulation of ZO-1 and ZO-3 at TJ requires CGN binding to NM2B

**Figure 6.**
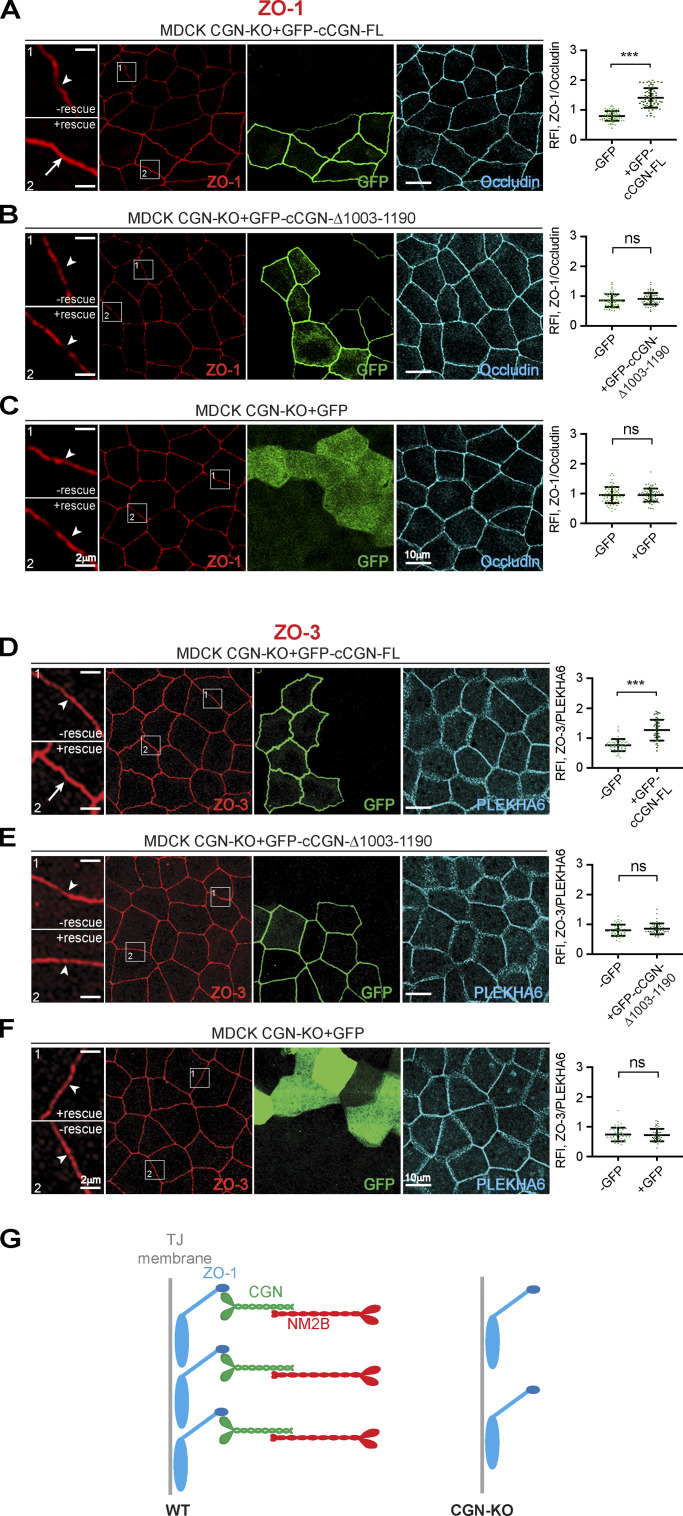
**CGN binding to NM2B is required for the normal accumulation of ZO-1 and ZO-3 at TJ. (A–F)** IF microscopy localization of endogenous ZO-1 (A–C) and ZO-3 (D–F) in CGN-KO MDCK cells expressing either GFP-tagged full-length CGN (A and D), or C-terminally truncated CGN (cCGN-Δ1003-1190; B and E) or GFP (C and F; labeled with anti-GFP antibodies). High magnification panels correspond to highlighted white box in low magnification micrograph; scale bars = 10 μm (low magnification) and 2 μm (high magnification). Arrowheads indicate reduced ZO-1 and ZO-3 labeling in CGN-KO cells. Arrows indicate increased ZO-1 and ZO-3 labeling in cells expressing full-length CGN. Quantification (histograms on the right) of relative fluorescent intensity (RFI) shows the ratio between the junctional staining of ZO-1 versus either occludin (A–C) or PLEKHA6 (D and E), used as junctional reference markers. Data are represented as mean ± SD (ZO-1 *n* = 72 from three independent experiments and ZO-3 *n* = 48 from two independent experiments). Statistical significance, unpaired Mann–Whitney’s test. ***P ≤ 0.001. **(G)** Scheme depicting reduced accumulation of ZO-1 in CGN-KO cells.

Next, we analyzed the functional consequences of NM2 interaction with CGN and CGNL1. We previously showed that the KO of CGN but not CGNL1 results in decreased TJ accumulation of ZO-1 and ZO-3, destabilization of junctional ZO-1, and increased folded conformation of ZO-1 (Vasileva et al., 2022). To determine whether the interaction of CGN with NM2B is mechanistically implicated in this phenotype, we rescued CGN-KO cells with either full-length or a C-terminally truncated construct of CGN, which lacks the NM2BR. IF microscopy analysis showed that while expression of full-length CGN rescued the reduced junctional labeling for ZO-1 (Fig. 6 A) and ZO-3 (Fig. 6 D), the C-terminally truncated CGN (cCGN-Δ1003-1090) and GFP alone failed to rescue normal junctional labeling of ZO-1 (Fig. 6, B and C) and ZO-3 (Fig. 6, D–F). These results indicate that interaction of CGN with NM2B is required for normal accumulation of ZO-1 and ZO-3 at TJ (scheme in Fig. 6 G).

### The accumulation of phalloidin labeling at TJ requires the CGN NM2BR and CGN promotes the TJ proximity of γ-actin

**Figure 7.**
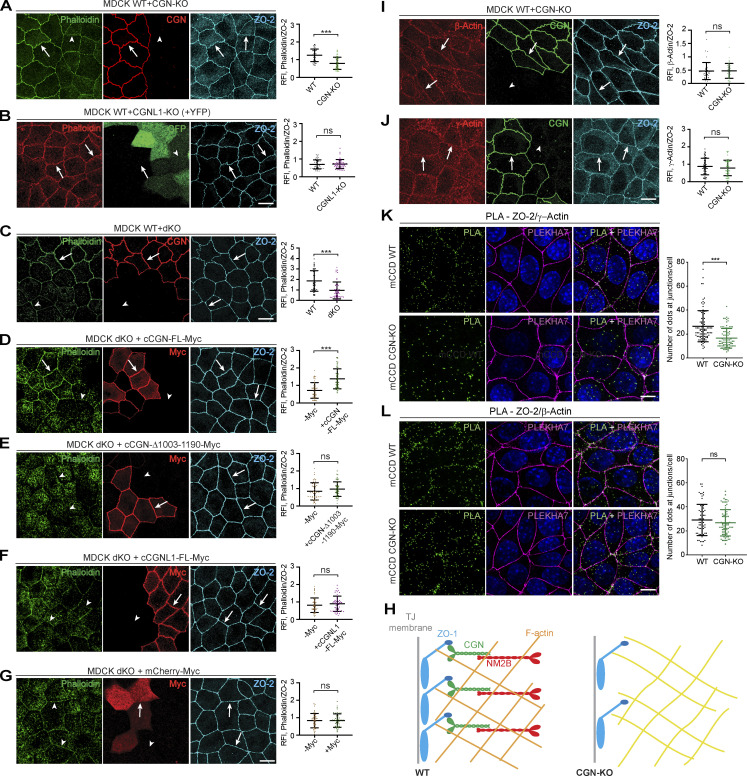
**CGN binding to NM2B promotes junctional phalloidin labeling and CGN is required for TJ proximity of γ-actin. (A–G)** IF microscopy localization of phalloidin (green channel, except for red in panel B), endogenous CGN (red, A and C), GFP (to identify CGNL1-KO cells, green in B), exogenously expressed myc-tagged rescue constructs (red, D–G) and ZO-2 used as a junctional reference marker (A–G, magenta) in either mixed cultures of CGN-KO and WT (A), CGNL1-KO and WT (B), double-KO (dKO) and WT (C) and rescued double-KO (D–G) MDCK cells. Double-KO cells were rescued with either myc-tagged FL CGN (D), or C-terminally truncated (CGN-Δ1003-1190) CGN (E), or myc-tagged FL CGNL1 (F), or mCherry-myc (negative control; G). Quantifications of relative fluorescent intensity (RFI) on the right of IF panels show the ratio between the junctional staining of phalloidin versus ZO-2. Data are represented as mean ± SD (*n* = 48 from two independent experiments). **(H)** Simplified scheme of the effect of CGN-KO on phalloidin labeling and ZO-1 junctional accumulation. Orange and yellow colors of schematic actin filaments indicate stronger and weaker phalloidin labeling, respectively. The filaments are shown as a branched network (Heuzé et al., 2019), either under tension (WT, orange) or relaxed (yellow, KO; see Discussion). ZO-1 accumulation at TJ is reduced in CGN-KO cells (scheme). **(I and J)** IF microscopy analysis and quantification of β-actin (I) and γ-actin (J) in mixed cultures of WT and CGN-KO MDCK cells. Arrows indicate junctional labeling, arrowheads indicate junctions between CGN-KO cells (β-actin *n* = 42 and γ-actin *n* = 48 from two independent experiments). **(K–L)** In situ PLA (green) to detect proximity between endogenous γ-actin (K) or β-actin (L) and ZO-2 in either mCCD WT or CGN-KO cells labeled with anti-PLEKHA7 (magenta) as a junctional reference marker. Nuclei are stained in blue. PLA dots quantification on the right side of the panel (K: *n* = 90 for WT cells and *n* = 83 for CGN-KO cells; L: *n* = 70 for WT cells and *n* = 73 for CGN-KO cells, from two independent experiments). Data are represented as mean ± SD. Statistical significance (A–G and I–L) was determined by unpaired Mann–Whitney’s test, ***P ≤ 0.001. Scale bars = 10 μm.

**Figure S6.**
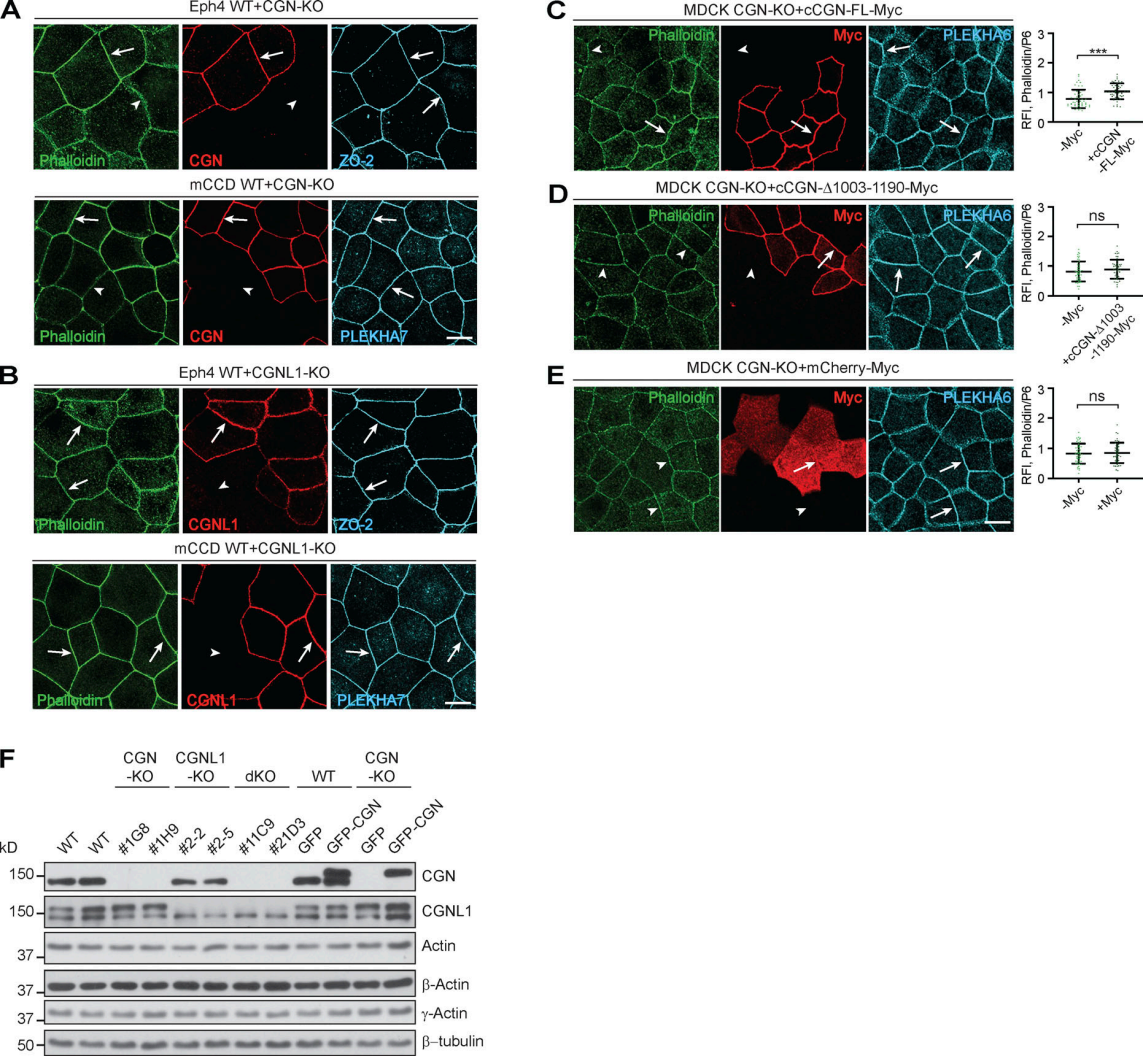
**CGN regulates actin filament organization at tight junctions of mCCD and Eph4 cells.** (Related to Fig. 7.) **(A–E)** IF microscopy analysis of phalloidin labeling either in mixed cultures of either WT + CGN-KO (A) or WT- + CGNL1-KO (B) Eph4 (top panels) and mCCD (bottom panels) cells, or in CGN-KO MDCK cells rescued either with myc-tagged full-length (FL) CGN (C), or C-terminally truncated CGN (D, Δ1003-1190), or mCherry (E, negative control). Either PLEKHA7 or ZO-2 or PLEKHA6 were used as junctional reference marker in mCCD or EpH4 or MDCK cells, and exogenous proteins were detected with antibodies against the myc tag. Arrows = normal labeling and arrowheads = reduced/undetectable cytoplasmic labeling. **(F)** IB analysis using antibodies against CGN, CGNL1, total actin, β-actin, and γ-actin (β-tubulin as loading control) of lysates from different clonal lines of MDCK cells (WT, CGN-KO, CGNL1-KO, double-KO [dKO]) and from CGN-KO MDCK cells rescued with either GFP-cCGN-myc or GFP-myc. Numbers on the left show migration of prestained markers. The scale bars (normal magnification panels, A–E) represent 10 μm. **(C–E)** Data in plots are represented as mean ± SD (*n* = 48 from two independent experiments). Statistical significance was calculated with unpaired Mann–Whitney test (***P < 0.001). RFI, relative fluorescence intensity. Source data are available for this figure: SourceData FS6.

Since myosins organize actin networks, we explored the role of CGN and CGNL1 in the organization of junctional actin filaments using phalloidin, which binds to actin filaments. In mixed confluent cultures of WT and KO MDCK cells, the KO of either CGN alone or of both CGN and CGNL1 resulted in a significant decrease in junctional labeling for phalloidin (arrowheads, Fig. 7, A and C, quantifications on the right). Instead, phalloidin labeling in CGNL1-KO cells was similar to WT cells (arrows, Fig. 7 B). A decrease in intensity in phalloidin junctional labeling was also observed in CGN-KO Eph4 and mCCD cells (arrowheads, Fig. S6 A), again with no detectable change upon KO of CGNL1 (arrows, Fig. S6 B). The junctional labeling for phalloidin was rescued in MDCK double-KO and CGN-KO cells by expression of full-length CGN (arrows, Fig. 7 D and Fig. S6 C, quantifications on the right), but not by expression of either the CGN mutant lacking the NM2BR (arrowheads, Fig. 7 E and Fig. S6 D), or CGNL1 (arrowhead, Fig. 7 F), or GFP alone (arrowhead, Fig. 7 G and Fig. S6 E).

Next, we examined the localization and expression of β-actin and γ-actin using isoform-specific monoclonal antibodies (Dugina et al., 2009). Junctional and cytoplasmic labeling (Fig. 7, I and J) for β-actin and γ-actin were similar in WT and CGN-KO cells (Fig. 7, I and J). IB analysis showed that the levels of expression of total actin, β-actin, and γ-actin were similar in different clonal lines of WT, CGN-KO, CGNL1-KO, double-KO, and rescued MDCK cells (Fig. S6 F). Next, we used the proximity ligation assay (PLA) to gauge the proximity between either γ-actin or β-actin and the TJ marker ZO-2. PLA signal for γ-actin was significantly reduced at TJ of CGN-KO cells, when compared to WT (Fig. 7 K), whereas the signal for β-actin was similar in WT and CGN-KO cells (Fig. 7 L).

In summary, these results show that accumulation of phalloidin labeling at junction and the TJ proximity of γ-actin require binding of CGN to NM2B and suggest that KO of CGN results in relaxation of juxta-membrane actin filaments (scheme, Fig. 7 H).

### TJ membrane tortuosity requires CGN and CGNL1 binding to NM2s and depends on NM2 activity

**Figure 8.**
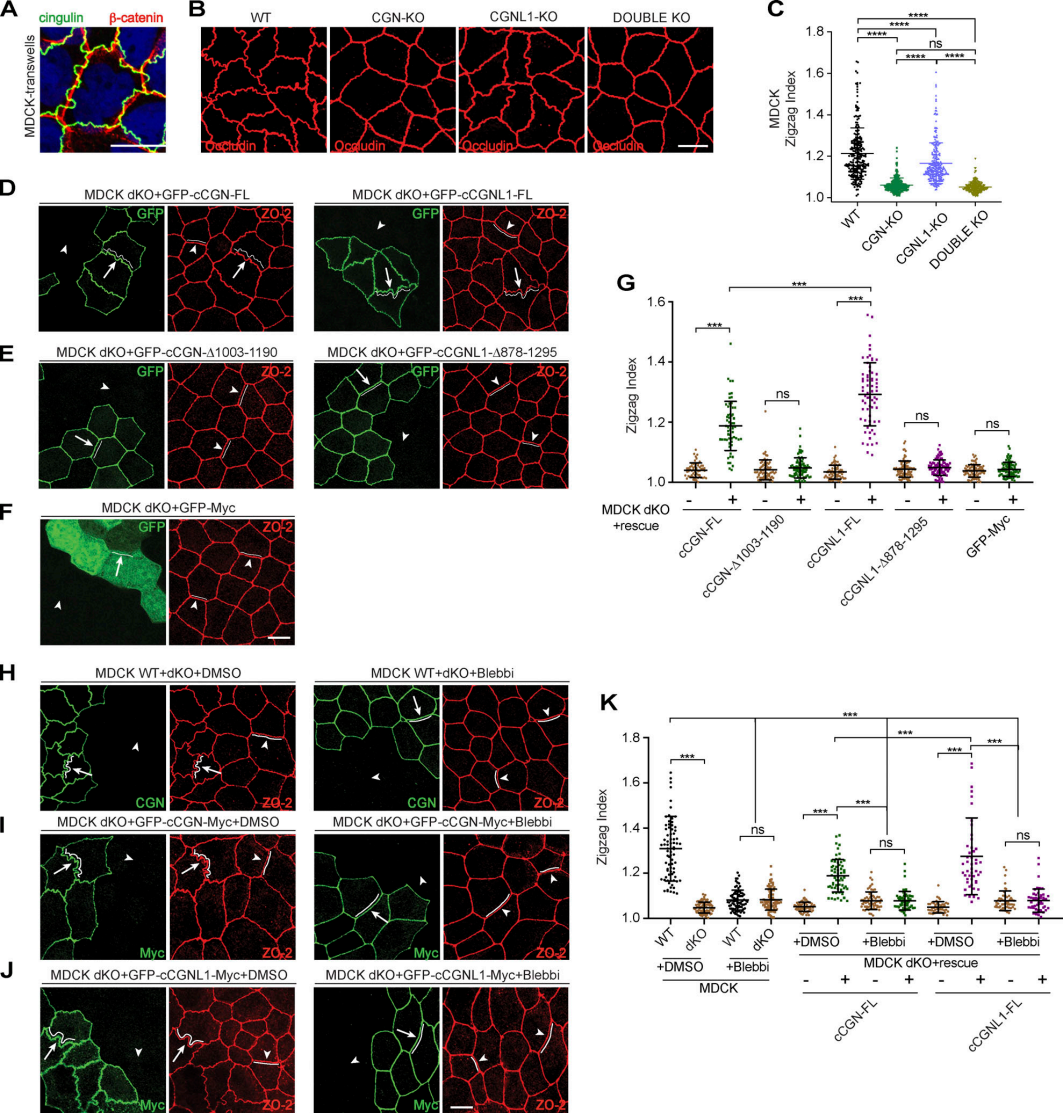
**CGN binding to NM2B and NM2 activity is required to maintain TJ membrane tortuosity in MDCK cells. (A–C)** IF microscopy localization of β-catenin (red) and CGN (green) in WT MDCK cells (A), of occludin (red) in WT, CGN-KO, CGNL1-KO, and CGN/CGNL1-double-KO (dKO) MDCK clonal cell lines (B), and quantifications of membrane tortuosity (ZI) in WT, CGN-KO, CGNL1-KO, and CGN/CGNL1-double-KO cells (C). Dots show replicates (*n* = 220) and bars represent mean ± SD. Statistical significance was determined by one-way ANOVA with post hoc Dunnett’s test (****P < 0.0001). **(D–G)** IF microscopy analysis of double-KO cells rescued either with full-length (FL) CGN (D, left panels) or CGNL1 (D, right panels), or with C-terminally truncated CGN (E, left panels) or C-terminally truncated CGNL1 (E, right panels), or GFP (F), and quantifications of ZI in CGN/CGNL1-double-KO cells rescued with the indicated constructs (G) were measured with TJ marker ZO-2. Lines in ZO-2 channel trace membranes to highlight tortuosity. (−) and (+) refer to the same cell line without and with exogenous expression of rescue construct. Dots show replicates (*n* = 60–87) and bars represent mean ± SD. Statistical significance was determined by unpaired Mann–Whitney’s test, ***P ≤ 0.001. **(H–K)** IF microscopy localization (H–J) of CGN (green, to detect WT vs. double-KO cells) and the TJ marker ZO-2 (red) either in WT cells (H), or in double-KO cells rescued with FL CGN (I), or FL cCGNL1 (J), and quantification of ZI (K) in the indicated cell lines (WT, double-KO, rescue, and treatments with either DMSO or blebbistatin [blebbi]). In H–J, the panels on the left show cells treated with DMSO (negative control) and the panels on the right indicate cells treated with blebbistatin (4 h, 50 μM). White lines and arrows/arrowheads trace membranes to highlight the shape of the TJ membrane. Arrowheads indicate junctions in double-KO cells. Scale bars = 10 μm. Dots in K show replicates (*n* = 50–78) and bars represent mean ± SD. Statistical significance was determined by unpaired Mann–Whitney’s test, ***P ≤ 0.001.

**Figure S7.**
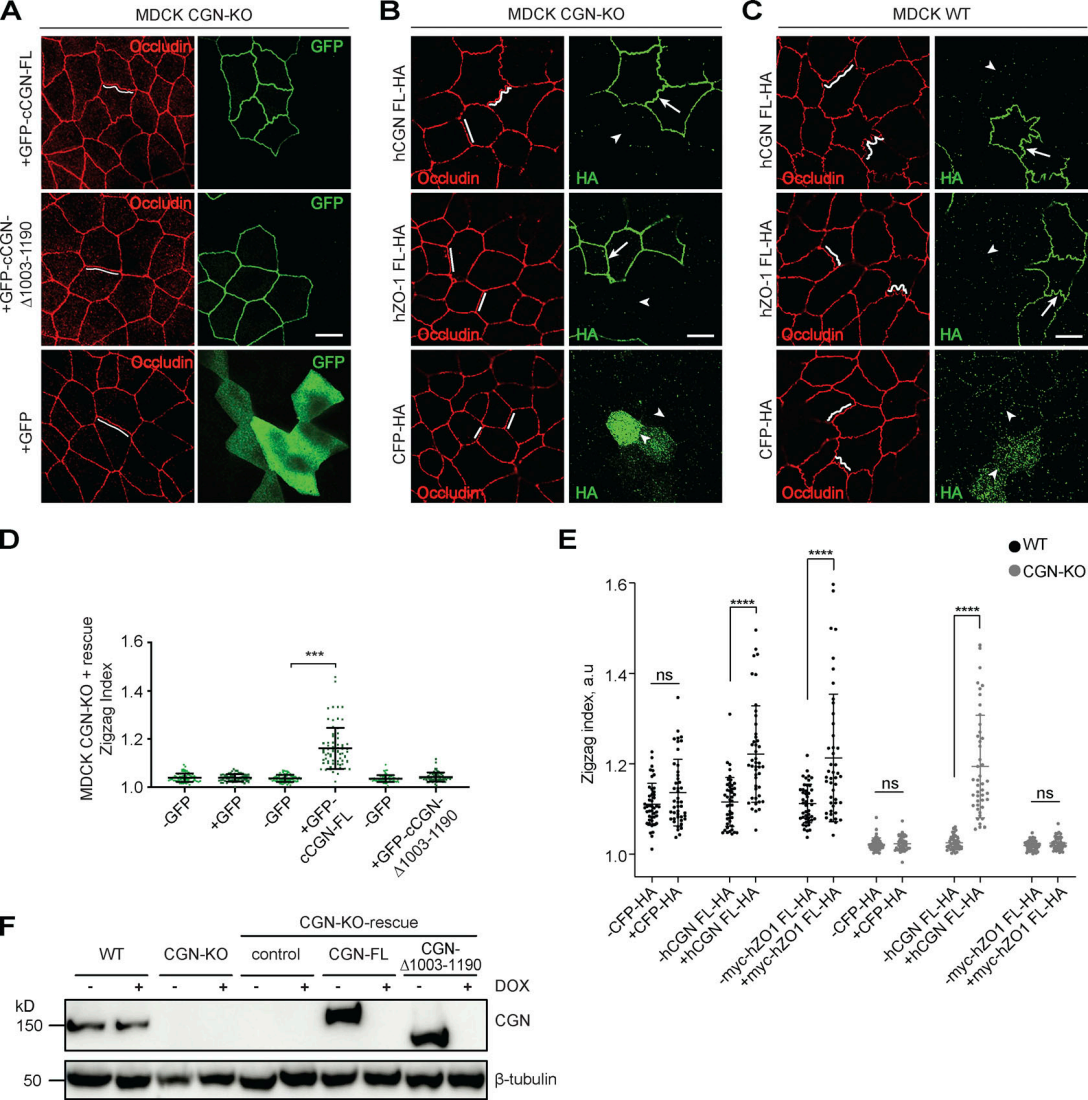
**TJ membrane tortuosity is regulated by CGN interaction with NM2B.** (Related to Figs. 8 and 9.) **(A and D)** IF microscopy localization of endogenous occludin and GFP-tagged exogenous CGN constructs (A) and quantification of ZI (D) in CGN-KO MDCK cells. Cells were rescued with either full-length (FL) CGN (top panel), or CGN lacking the NM2BR (middle panel), or GFP alone (bottom panel, negative control). Lines in occludin channel trace membranes to highlight tortuosity. Data are represented as mean ± SD (*n* = 67–80 from three independent experiments). Statistical significance was determined by unpaired Mann–Whitney test, ***P < 0.001. **(B, C, and E)** IF microscopy localization (B and C) of endogenous occludin in either CGN-KO (B) or WT (C) MDCK cells overexpressing either HA-tagged CGN (top panels), or HA-tagged ZO-1 (middle panels), or HA-CFP (bottom panels, negative control) and quantification of ZI (E). Dots in E show replicates (*n* = 45 junctional segments from three independent experiments), and data are represented as mean ± SD. Statistical significance was determined by unpaired Mann–Whitney test, ****P < 0.0001. Scale bars (A–C), 10 μm. **(F)** IB analysis of the expression of endogenous and exogenous CGN constructs (either CGN-FL orCGN-Δ1003-1190) either in WT MDCK cells, or CGN-KO cells, or CGN-KO cells inducibly rescued with the indicated constructs for the measurement of apical membrane stiffness (Fig. 9). Source data are available for this figure: SourceData FS7.

The forces applied to the AJC determine the shape of the TJ membrane (Tokuda et al., 2014; Tang, 2018). In WT MDCK cells, the TJ membrane showed a tortuous shape, as shown by labeling for CGN (green, Fig. 8 A), whereas β-catenin labeling, corresponding to the circumferential AJ, was straight (red, Fig. 8 A). To quantify TJ membrane tortuosity, we used the zigzag index (ZI; Tokuda et al., 2014). KO of CGN or both CGN and CGNL1 resulted in less tortuous occludin labeling (Fig. 8 B), as indicated by a significant decrease in the ZI (quantification, Fig. 8 C). KO of CGNL1 alone resulted in a smaller decrease in the ZI (Fig. 8, B and C). The decreased TJ membrane tortuosity of CGN-KO and double-KO MDCK cells was rescued by re-expression of either full-length CGN or CGNL1 (Fig. 8 D, quantification in Fig. 8 G, and Fig. S7 A, top panels, quantification Fig. S7 D), but was not rescued either by CGN and CGNL1 constructs lacking the NM2BR (Fig. 8 E, quantification in Fig. 8 G; and Fig. S7 A, middle panels, quantification Fig. S7 D) or by GFP alone (Fig. 8 F and Fig. S7 A, bottom panels, quantifications in Fig. 8 G and Fig. S7 D). Importantly, TJ membrane tortuosity of CGN-KO cells was not rescued by exogenous expression of ZO-1 (Fig. S7 B, middle panels, quantification in Fig. S7 E) whereas in WT cells, it was increased by exogenous expression of either CGN or ZO-1 (Fig. S7 C, quantification in Fig. S7 E), demonstrating that ZO-1 requires CGN to affect TJ membrane tortuosity.

To determine how NM2 activity contributes to TJ membrane tortuosity, we treated cells with blebbistatin (Fig. 8, H–K). Blebbistatin decreased the ZI of WT cells but had no effect on double-KO cells, which showed straight TJ (Fig. 8 H, quantification in Fig. 8 K). Rescue of double-KO cells with either CGN or CGNL1 (Fig. 8, I and J, quantification in Fig. 8 K) resulted in an increase in the ZI, and the increase in tortuosity was reverted by treatment with blebbistatin (Fig. 8, I and J, quantification in Fig. 8 K).

Together, these observations show that tethering of NM2s by CGN and CGNL1 promotes TJ membrane tortuosity, which depends on myosin-dependent contractility.

### CGN regulates apical surface stiffness by binding to NM2B

**Figure 9.**
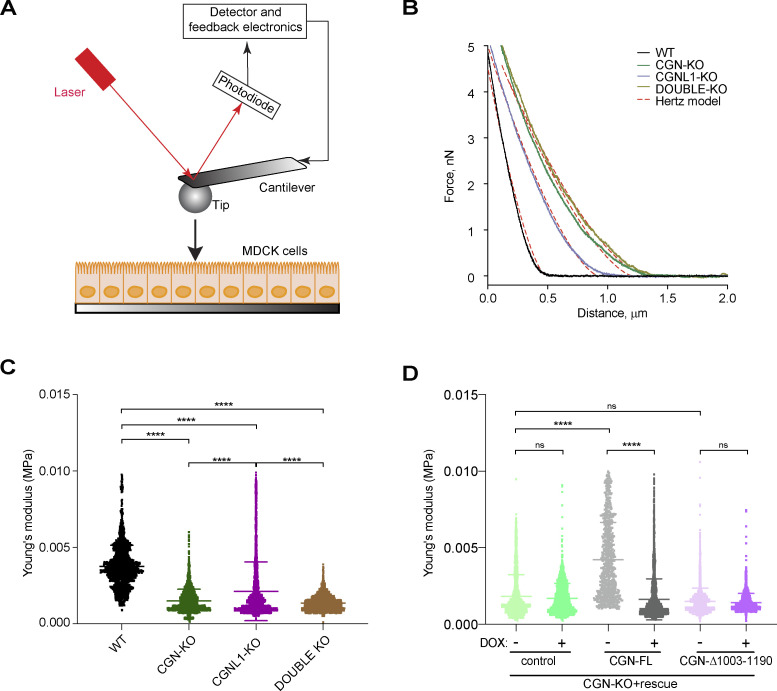
**CGN controls apical membrane stiffness by binding to NM2B. (A)** Schematic diagram of experimental setup for AFM. **(B)** Representative AFM force-indentation curves of WT, CGN-KO, CGNL1-KO, and CGN/CGNL1-double-KO (dKO) MDCK cell lines fitted with Hertz (Spherical) model. **(C)** Averaged stiffness (Young’s modulus) of WT, CGN-KO, CGNL1-KO, and CGN/CGNL1-double-KO MDCK cell lines (*n* = 1,908 for WT, *n* = 2,445 for CGN-KO, *n* = 2,118 for CGNL1-KO and *n* = 3,061 for dKO). **(D)** Averaged stiffness (Young’s modulus) of CGN-KO cells with no rescue (control), or rescued with either full-length CGN (CGN-FL), or with C-terminally truncated CGN (CGN-Δ-1003-1190) either in the absence (−) or in the presence (+) of doxycycline (DOX), which represses rescue transgene expression (*n* = 2,002 for control –DOX, *n* = 1,992 for control +DOX, *n* = 877 for CGN-FL –DOX, *n* = 4,536 for CGN-FL DOX, *n* = 2,592 for CGN-Δ-1003-1190 –DOX and *n* = 2,307 for CGN-Δ-1003-1190 +DOX). **(C and D)** Statistical significance was determined by Kruskal–Wallis test followed by Dunn’s multiple comparison, ****P < 0.0001.

Since CGN and CGNL1 tether specific NM2 isoforms to the AJC, and actomyosin regulates the mechanics of the plasma membrane (Brückner and Janshoff, 2015), we asked whether CGN and CGNL1 regulate the stiffness of the apical plasma membrane. Atomic force indentation microscopy (AFM) was used to measure the local micro-elasticity and stiffness of the apical surface of epithelial monolayers, by determining the relationship between applied mechanical stress and deformation (Fig. 9 A). Force-indentation curves of MDCK cells were fitted by Hertz model (Harris and Charras, 2011; Fig. 9 B) to obtain the Young’s modulus (Fig. 9, C and D; and Table 2). The Young’s modulus of CGN-KO and double-KO MDCK cells was less than half the value of WT cells (e.g., 0.0015 and 0.0014 MPa, compared to 0.0037 MPa, Fig. 9 C and Table 2), indicating a significant loss of stiffness of CGN-KO and double-KO cells. The decrease in Young’s modulus for CGNL1-KO MDCK cells was smaller (0.0021 MPa), likely due to the low levels of expression of CGNL1 in MDCK cells, indicating that in MDCK cells CGN is epistatic to CGNL1 with regard to apical membrane stiffness. Importantly, when CGN-KO cells were rescued with full-length CGN, but not with C-terminally truncated CGN, the Young’s modulus was significantly increased (Fig. 9, D, IB analysis of rescued cells in Fig. S7 F). These findings indicate that tethering of NM2s to apical junctions by CGN and to a lesser extent by CGNL1 regulates apical plasma membrane stiffness.

**Table 2.** Young’s modulus values for MDCK lines

Line genotype	Young’s modulus (MPa)
WT	0.0037 ± 0.0014
CGN-KO	0.0015 ± 0.0007
CGNL1-KO	0.0021 ± 0.0019
CGN/CGNL1-double-KO	0.0014 ± 0.0005

### CGNL1 binding to NM2s is required to maintain the linear integrity of the AJ complexes

**Figure 10.**
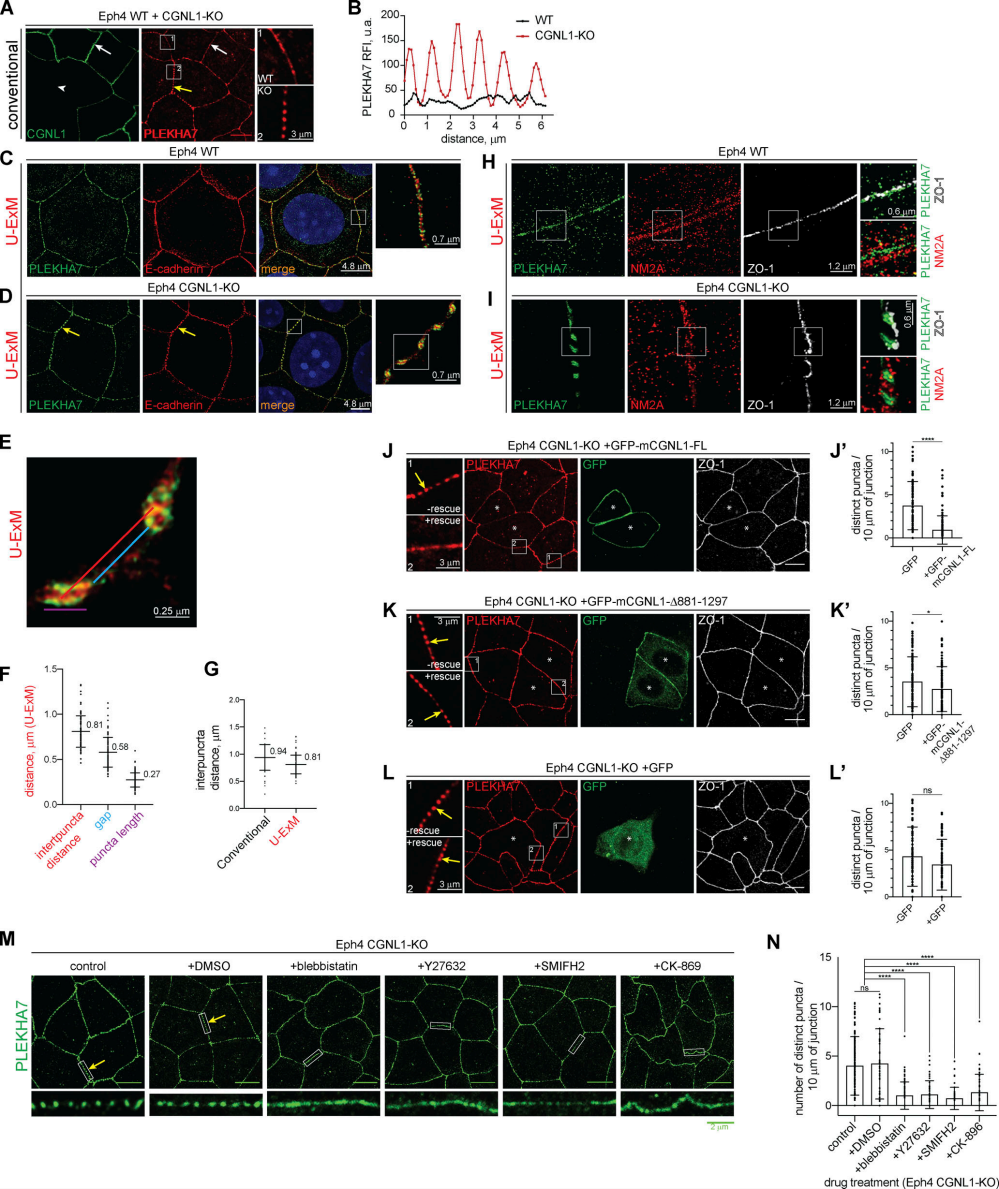
**CGNL1 binding to NM2s is required to prevent the fragmentation of AJ protein complexes into discrete puncta. (A and B)** IF microscopy analysis of the localization of PLEKHA7 (A) and profile of labeling intensity of PLEKHA7 (B) of the localization of PLEKHA7 in mixed cultures of WT and CGNL1-KO Eph4 cells. Insets on the right of images in A show high magnification details of areas outlined in square boxes. White arrows indicate linear junctional labeling. White arrowheads indicate gaps in junctional labeling. Yellow arrows indicate PLEKHA7 puncta in CGNL1-KO cells. **(C–G)** U-ExM analysis (C–E) and measured distances (F and G) of the localization of PLEKHA7 (green) and E-cadherin (red) in Eph4 WT cells (C) or CGNL1-KO (D and E) cells. Insets on the right of images in C and D show high magnification details of areas outlined in square boxes. Yellow arrows indicate punctate PLEKHA7/E-cadherin signal. **(E)** High magnification of area outlined in the inset of D showing the different distances/length quantified in F. **(F)** Quantification of the inter-puncta distance (red), gap between two puncta (light blue), and length of the puncta (purple) as depicted in E. Dots show replicates (*n* = 82–100) and bars represent mean ± SD. **(G)** Comparison of inter-puncta distances calculated on conventional and U-ExM images. Dots show replicates (*n* = 82–131) and bars represent mean ± SD. **(H and I)** IF microscopy analysis of the localization of PLEKHA7 (green), NM2A (red), and ZO-1 (white) in expanded (U-ExM) Eph4 WT (H) or CGNL1-KO (I) cells. Insets on the right of images show high magnification details of areas outlined in square boxes (double color channel, indicated on the right). **(J–L′)** IF microscopy analysis and quantification of PLEKHA7 dots in CGNL1-KO Eph4 cells transfected with GFP-tagged full-length (FL) CGNL1 (J and J′), CGNL1 lacking the NM2-binding site (Δ881-1297; K and K′) or GFP alone (L and L′). Insets on the left of images show high magnification details of areas outlined in square boxes. Asterisks indicate transfected cells and yellow arrows indicate punctate PLEKHA7 signal. **(J′–L′)** Dots show replicates (*n* = 64–132) and bars represent mean ± SD. **(M and N)** IF microscopy analysis (M) and quantification of number of PLEKHA7 puncta (N) in CGNL1-KO Eph4 cells, either untreated (control) or treated with solvent (DMSO) or treated with drugs that affect the contractility and polymerization of the actomyosin cytoskeleton. Note that control and DMSO-treated cells show punctate PLEKHA7 labeling, (yellow arrows) which is attenuated after treatment with the drugs. Dots show replicates (*n* = 41–180) and bars represent mean ± SD. Unless otherwise indicated on the micrographs (A, C–E, and H–M), scale bar = 10 µm. Data in J′–L′ and N are represented as mean ± SD. Statistical significance was determined by unpaired Mann–Whitney test (J′–L′) and Kruskal–Wallis test followed by Dunn’s multiple comparison (N), *P < 0.05, ****P < 0.0001.

The weak phenotypes of CGNL1-KO MDCK with regard to the junctional accumulation of ZO proteins and phalloidin, TJ membrane tortuosity, and apical membrane stiffness could be due to the low affinity of binding between CGNL1 and ZO-1 (Fig. S5, C and D), and/or to low levels of CGNL1 mRNA and protein expression in MDCK cells (Fig. S3, A and B; Vasileva et al., 2017), and/or to the localization of CGNL1 at AJ (Ohnishi et al., 2004). To further study the role of CGNL1–NM2 interaction in the AJC, we used Eph4 cells, which express higher levels of CGNL1 compared to MDCK cells (Fig. S3 B), and we focused on the architecture of the AJ. Either mixed or separate cultures of WT and CGNL1-KO Eph4 cells were analyzed either by conventional IF confocal microscopy (Fig. 10 A) or by ultra-expansion IF microscopy (U-ExM; Fig. 10, C–E; and Fig. 10, H and I). WT cells showed a homogeneous linear distribution of labeling for the AJ markers PLEKHA7 and E-cadherin (Fig. 10, A, C, and H). In contrast, CGNL1-KO cells showed a fragmentation of the PLEKHA7 labeling into distinct puncta (insets in Fig. 10, A, D, and I). The distance between the puncta was ≈0.9 μm, the empty gap between puncta was ≈0.66 μm, and each punctum had a length of ≈0.27 μm (Fig. 10, E and F). The inter-puncta distance was similar when calculated on conventional (0.94 μm) or U-ExM (0.81 μm) microscopy images (Fig. 10 G). U-ExM showed that in WT cells ZO-1 and PLEKHA7 were closely colocalized and homogeneously distributed near the junctional membrane (white and green in magnified inset, Fig. 10 H), whereas NM2A labeling was in a peri-junctional localization, farther away from the membrane (red in magnified inset, Fig. 10 H). In CGNL1-KO cells, PLEKHA7 signal was fragmented into puncta (arrowheads, Fig. 10 I), but ZO-1 labeling was detectable in the inter-puncta spaces (arrows, white signal, Fig. 10 I), consistent with previous observations (Vasileva et al., 2022), suggesting that fragmentation only concerns the AJ plaque, and not the TJ plaque. NM2A labeling was detected in the inter-puncta space (arrow, red signal, magnified inset in Fig. 10 I).

Next, to ask whether the linkage of CGNL1 to NM2s was involved in the AJ puncta phenotype, we rescued CGNL1-KO Eph4 cells with either FL or C-terminally truncated CGNL1. IF microscopy showed that while full-length CGNL1 rescued the fragmentation of PLEKHA7 labeling into puncta (Fig. 10 J, quantification in Fig. 10 J′), no rescue was observed when using either the CGNL1 construct lacking the NM2BR (Fig. 10 K, quantification in Fig. 10 K′) or GFP alone (Fig. 10 L, quantification in Fig. 10 L′).

Finally, to determine whether the fragmentation of PLEKHA7 labeling into puncta was a consequence of actomyosin-generated force, we treated CGNL1-KO cells with drugs that affect the contractility and polymerization of actomyosin. Treatment of cells with either blebbistatin, Y27632, SMIFH2 or CK-869, which inhibit either myosin activity, or myosin activation by the Rho-ROCK pathway, or linear or branched actin polymerization, resulted in significant decrease in the fragmentation of PLEKHA7 labeling into puncta (Fig. 10 M, quantification Fig. 10 N).

Together, these results suggest that by tethering NM2s to the AJ, CGNL1 spreads the spatially uneven force from the AJ-associated peri-junctional actomyosin belt to maintain a homogeneous linear distribution of AJ plaque proteins.

## Discussion

The mechanisms driving the subcellular sorting of NM2 and actin isoforms and their mechanical coupling to distinct cellular compartments is a biological question of fundamental importance, since actomyosin contractility must occur at specific spatial sites in the cell, and not randomly. At junctions, co-polymerization of NM2B with NM2A (Beach et al., 2014; Shutova et al., 2014; Melli et al., 2018; Shutova and Svitkina, 2018a) could account for the presence of NM2B within the peri-junctional circumferential belt (Smutny et al., 2010; Heuzé et al., 2019). However, the mechanisms underlying the juxta-membrane localization of NM2B in a region lacking NM2A (Heuzé et al., 2019) remain unclear. The results presented here support a model where CGN and CGNL1 recruit and stabilize selected NM2 isoforms to the AJC plaque, and specifically NM2B in the juxta-membrane region, through direct interactions between their coiled-coil rod domains. This model is based on the direct and selective binding of purified full-length and rod fragments of CGN and CGNL1 to purified rod fragments of NM2A and NM2B isoforms, coupled with the effect of KO, rescue and exogenous expression of CGN and CGNL1 WT and mutant constructs on the isoform-specific localization of NM2s in different cell lines, consistent with the in vitro binding results.

Previously proposed mechanisms of spatial sorting of NM2 isoforms include different rates of filament assembly and turnover and different duty ratios for NM2A and NM2B (Sandquist and Means, 2008; Beach et al., 2014; Shutova et al., 2014), as well as NM2s interaction with proteins such as supervillin (Chen et al., 2003), anillin (Straight et al., 2005), septin-2 (Joo et al., 2007), Lgl (Dahan et al., 2012), S100 family proteins (Li and Bresnick, 2006; Ecsédi et al., 2018), 14-3-3 (Zhou et al., 2010), Unc54A (Lechuga et al., 2022), and Lmo7 (Matsuda et al., 2022; see also Dulyaninova and Bresnick, 2013; Beach and Hammer, 2015; Shutova and Svitkina, 2018b). However, unlike CGN and CGNL1, the proteins cited above are not specific components of the AJC, and for most of them it is not clear whether their interactions with NM2s are direct, or whether there is a NM2 isoform preference. Conversely, we show that NM2s interact directly with the C-terminal ∼200–250 residues of CGN and CGNL1, and that CGN and CGNL1 show preference for NM2B and NM2A/B, respectively, both in vitro and in cells. We also show that the KO of CGN leads to reduced junctional accumulation of NM2B, indicating that CGN tethering is required for junctional localization of NM2B. In contrast, the KO of CGNL1 did not result in decreased NM2A/B junctional labeling in the experimental models used here, although CGNL1 exogenous expression promoted the junctional accumulation of both NM2B and NM2A. Moreover, some diffuse peri-junctional labeling for NM2B was observed even in MDCK cells lacking both CGN and CGNL1. Together, these observations indicate that in addition to the interaction of NM2s with CGN and CGNL1, redundant mechanisms, such as co-assembly into existing filaments, are involved in NM2A and NM2B recruitment to junctions. Moreover, neither CGN nor CGNL1 appeared to control the localization of NM2C at junctions, indicating that alternative mechanisms must regulate this isoform.

The sequences of the NM2BRs of CGN and CGNL1 are highly conserved and homologous to NM2 rods, arguing for a physiological significance in direct NM2 tethering. Interestingly however, unlike NM2B rod, the purified rod fragments of CGN did not assemble into filaments, consistent with informatic prediction and published experimental data (Citi et al., 2000; D’Atri et al., 2002). In addition, the CGN Rod2 fragment inhibited the in vitro assembly of the NM2B rod, and the predicted favored interaction between CGN and NM2B is antiparallel. These observations suggest a model where CGN molecules are bound through their head domain to ZO-1, and in turn they tether either NM2B monomers (simplified schemes in Figs. 5 N, 6 G, and 7 H), or, potentially, dimers or oligomers, through antiparallel interaction of their Rod2 regions. Such a model represents a new paradigm for functional NM2, since bipolar filaments have been traditionally considered the only functional form of NM2s (Beach and Hammer, 2015). This model does not exclude the possibility that the rod regions of CGN and CGNL1 intercalate within myosin filaments, similarly to what observed for myosin-18 (Billington et al., 2015). Indeed, our observation that a CGN rod fragment co-pellets with NM2B rod filaments would support this hypothesis. However, several observations support the idea that monomeric/oligomeric myosin molecules may be present and active in specific subcellular contexts, such as the juxta-membrane region. First, monomeric/oligomeric nonmuscle myosin has been detected in cells. Quick-freeze-deep etch microscopy shows cross-linking strands in the terminal web of intestinal epithelial cells, which were proposed to be dimeric or oligomeric myosin molecules (Hirokawa and Heuser, 1981; Hirokawa et al., 1982). Platinum replica electron microscopy shows that fibroblasts contain activated monomers/dimers of NM2A and NM2B with phosphorylated light chains, which were proposed to link the actin cytoskeleton to organelles (Shutova et al., 2014). Second, at epithelial junctions, the juxtamembrane region interposed between the plasma membrane and the actomyosin circumferential belt, where NM2B is located, is very narrow (<200 nm; Efimova and Svitkina, 2018; Heuzé et al., 2019), and it is difficult to imagine how 250-nm-long NM2B bipolar filaments (Nagy et al., 2013) could fit in this space. Third, monomeric NM2B is non-processive (Nagy et al., 2013), and CGN-tethered monomeric NM2B would be ideally suited to act as a cross-linker to the network of branched actin filaments and maintain tensile stress if kept under load, consistent with its kinetic properties and high duty-ratio (Kovács et al., 2007; Melli et al., 2018). Determining the stoichiometry of interaction between CGN/CGNL1 and NM2s, the precise state of polymerization and mode of interaction of CGN/CGNL1 and NM2B in the juxta-membrane space, and the mechanisms of regulation of the interaction between CGN/CGNL1 and NM2s are challenging questions that await future studies.

The KO and rescue experiments we report here show that CGN and CGNL1 interaction with NM2s regulate the mechanics of junctional proteins and protein complexes, actin filaments, and the plasma membrane, and specifically, they affect: (1) the TJ accumulation and stretching of ZO-1 (CGN); (2) the junctional accumulation of phalloidin (CGN); (3) the apico-basal positioning of γ-actin (CGN); (4) the tortuosity of the TJ membrane (CGN and CGNL1); (5) the stiffness of the apical plasma membrane (CGN and CGNL1); and (6) the linear continuity of AJ protein complexes (CGNL1). The junctional accumulation of ZO-1 requires ZO-1 stretching and actomyosin-generated force (Spadaro et al., 2017) and ZO-1 stretching is promoted by CGN (Vasileva et al., 2022). Here we show that ZO-1 stretching requires the CGN NM2BR, and thus the formation of the ZO-1–CGN–NM2B complex. Thus, CGN mechanically couples ZO-1 to the actomyosin cytoskeleton, and this fact should be considered when analyzing the roles of ZO-1 in epithelial mechanobiology. Similarly, junctional phalloidin was decreased in CGN-KO cells and was rescued only by CGN constructs that bind to NM2B, while total β-actin and γ-actin labeling were not affected. This suggests that the decreased phalloidin labeling is due to a NM2-dependent mechanical relaxation of actin filaments, rather than to a decrease in the number of filaments. This is consistent with the observation that junctional phalloidin labeling is also reduced upon treatment of cells with blebbistatin (Smutny et al., 2010), and that phalloidin binding to actin filaments depends on their structural dynamics, flexibility, thermal fluctuations, and mechanical stress (Allen et al., 1996; De La Cruz and Pollard, 1996; Jégou and Romet-Lemonne, 2021). Moreover, the reduced TJ proximity of γ-actin in CGN-KO cells also suggests a role of CGN in the spatial sorting of the γ-actin isoform to TJ. Importantly, the KO of CGNL1 did not affect phalloidin labeling, correlating with no effect of CGNL1 KO and exogenous expression on junctional accumulation of ZO-1 (Vasileva et al., 2022), and no formation of a ZU5–CGNL1–NM2B complex. Together, these observations suggest that in confluent MDCK cells NM2B and the juxta-membrane branched actin network are mostly tethered to TJ and maintained under tensile stress by the ZO-1–CGN–NM2B complex, whereas CGNL1 does not tether NM2B to ZO-1 in MDCK cells. In contrast, NM2 anchoring by CGNL1 to AJ prevented force-induced fragmentation of the AJ. Since previous studies showed that depletion of NM2B induces fragmentation of E-cadherin labeling (Smutny et al., 2010), this suggests that the NM2B–CGNL1 interaction occurs at AJ and allows to evenly spread to the junctional scaffolding complexes the spatially discontinuous tension generated by the mini-sarcomeric arrangement of myosin filaments (Ebrahim et al., 2013) within the peri-junctional circumferential actomyosin belt. However, since exogenous expression experiments indicate that CGNL1 also promotes the junctional accumulation of NM2A, CGNL1-mediated mechano-transduction may also implicate NM2A, through mechanisms that remain to be investigated.

Mechanical coupling of NM2 isoforms by CGN/CGNL1 also impacts on the tortuosity of the TJ membrane. Although ZO-1–depleted MDCK cells show straight TJ (Van Itallie et al., 2009; Tokuda et al., 2014), in the absence of CGN exogenous expression of ZO-1 was not sufficient to promote TJ membrane tortuosity. Thus, altered junctional levels of claudin-2, which correlate with ZO-1 levels at junctions (Lynn et al., 2020), are unlikely to be mechanistically involved in regulating TJ membrane tortuosity. Instead, our data support a model where ZO-1 regulates TJ membrane tortuosity and NM2 localization at junctions indirectly, by recruiting CGN, and hence establish a tether to NM2B, at TJ. Tortuosity results from the balance between forces that are either parallel or orthogonal to the TJ membrane (Tang, 2018). Expression of either CGN or CGNL1 in double-KO cells, which have straight TJ, promotes tortuosity and correlates with increased junctional NM2B. Since TJ membrane tortuosity and AJ fragmentation are inhibited by blebbistatin (Figs. 8 and 10 and Tokuda et al., 2014), this suggests that CGN/CGNL1-mediated tethering of NM2B allows the transduction of orthogonal forces generated by the circumferential actomyosin bundle to the scaffolding junctional complexes. Moreover, since the KO of CGN was not associated with changes in myosin light chain phosphorylation, the decreased tortuosity observed in CGN-KO cells depends on the physical tethering of NM2B, rather than modulation of myosin light phosphorylation. With respect to barrier function, a direct correlation between TJ membrane tortuosity and permeability is not established (Tokuda et al., 2014; Lynn et al., 2020), and future studies will examine the barrier properties of monolayers of cells KO for CGN, CGNL1, or both.

Finally, the interaction of CGN family proteins with NM2s regulates apical plasma membrane stiffness, which depends on actomyosin organization (Brückner and Janshoff, 2015). The elastic modulus of WT MDCK cell was consistent with previously reported MDCK apical stiffness (Nehls et al., 2019), and the loss of CGN, and to a lesser extent of CGNL1, reduced the cell layer elastic stiffness, while monolayer integrity and junctions were maintained. This agrees with the observation that upon KO of NM2B in mice the apical mesh-like adhesion structure in cells lining the spinal canal collapses (Ma et al., 2007; Conti and Adelstein, 2008). Thus, our phenotype suggests that the CGN/CGNL1–NM2 interaction maintains a stiff apical surface by providing a circumferential junctional tether for the actomyosin cytoskeleton underlying the apical cortex.

Our conclusions on the role of NM2s in the mechanical phenotypes of our KO cells assume that deletion of the NM2BR has no other impact on the functions of CGN and CGNL1, besides the loss of NM2 binding. For example, the loss of the NM2BR could affect the conformation and function of other domains of CGN and/or CGNL1. Although we do not exclude this possibility, or the existence of other as yet unknown interactors of the NM2BR, several observations favor our conclusions. First, the sequences of the Rod2 regions of CGN and CGNL1 show high homology to the Rod domains of NM2s, arguing for a preferential binding to NM2s by coiled-coil interaction, which is supported by direct binding experiments using purified proteins. Second, upon deletion of the NM2BR both CGN and CGNL1 are still recruited to junctions, and in turn they recruit to junctions MgcRacGAP, GEF-1 (Fig. S2), and CAMSAP3 (Flinois et al., 2023), indicating no conformation-dependent changes in their ability to bind to their junctional partners through the head region, and to their known interactors through their Rod1 region. Third, as noted above, depletion of NM2B (Smutny et al., 2010) phenocopies the effect of KO of CGNL1 on the integrity of the AJ complex, which is not rescued by the CGNL1 mutant lacking the NM2BR. Finally, despite extensive searches, no other interactors of the CGN/CGNL1 Rod2 regions have so far been identified, and it is difficult to imagine which putative interactor(s) could have as important a role in mechanotransduction as NM2s.

In summary, we show that CGN and CGNL1 mechanically couple the actomyosin cytoskeleton to junctions by contributing to the recruitment and tethering of NM2B (CGN and CGNL1) and NM2A (CGNL1) and connecting NM2s to ZO-1 and to AJ complexes. This action of CGN and CGNL1 serves to regulate ZO-1 conformation, actin organization, the stiffness and shape of apical and junctional plasma membranes, and AJ linear integrity. These findings open new avenues for future studies on the regulation of NM2 interaction with CGN and CGNL1 and on the roles of CGN and CGNL1 in modulating filament assembly and activity of NM2s. Moreover, they provide a mechanistic framework to understand the roles of CGN and CGNL1 in tissues and organs in physiology and pathology, and highlight the crucial importance of NM2-binding proteins in the spatial regulation of actomyosin function.

## Materials and methods

### Experimental model and subject details

Eph4 (mouse mammary epithelial cell line, female; WT and ZO-1-KO), MDCK (Madin-Darby Canine Kidney II cell line, female), mCCD (mouse cortical collecting duct epithelial cell line) and HEK293T cells were cultured at 37°C, 5% CO_2_ in DMEM containing 10% or 20% FBS (for mCCD). For Eph4, MDCK, and mCCD culture media were supplemented with 1% non-essential amino acids, 100 U/ml penicillin, and 100 μg/ml streptomycin (Spadaro et al., 2017; Vasileva et al., 2017; Rouaud et al., 2019). We trusted cell providers for the authentication of the cell lines.

Cell lines KO for either CGN or CGNL1 or both were generated by CRISPR/Cas9 gene editing (Vasileva et al., 2022). Rescued stable cell lines were obtained by transfection with full-length and mutated plasmids using JetOptimus, and sorting single cells at 48 h after transfection (Beckman Coulter MoFlo Astrios sorter, Flow Cytometry Service, University of Geneva Medical School) into 96-well tissue culture plates. Single clones screened by immunoblot and immunofluorescence analysis. The phenotypes were confirmed on 2–3 distinct clonal lines.

Drugs treatments were as follows (final concentration, duration): blebbistatin (50 μM, 4 h), Y27632 (10 μM, 4 h), SMIFH2 (50 μM, 4 h), CK-869 (100 μM, 4 h), and DMSO (control solvent).

### Antibodies and IF

Antibodies are described in Table S1. The specificity of antibodies against NM2A (n. 909801; BioLegend), NM2B (n. 909901; BioLegend), and NM2C (n. 8189; Cell Signaling Technology) was verified in previous studies (Nguyen-Ngoc et al., 2017; Heuzé et al., 2019; Weißenbruch et al., 2021). The specificity of additional antibodies against NM2C (00111015; Covalab and 20716-1-AP; Proteintech) was verified by IB analysis lysates from MDCK cells treated with siRNAs targeting NM2C (Fig. S2, A–C). The Covalab anti-NM2C antibody was used for IB analysis (Fig. S2 J and Fig. S3, B, D, and E) and the Proteintech anti-NM2C antibody for IB analysis of GST pulldowns (Fig. S1, C and D). The cell-signaling anti-NM2C antibody was used for IF microscopy analysis (Fig. 3 and Fig. S3 C). The above-cited anti-NM2A and anti-NM2B were used for both IB and IF.

For IF, cells were cultured either on glass coverslips in 24-well plates for 3 d seeded at a density of 1–2 × 10^5^ cells/well or on 24-mm Transwell filters for 5 d seeded at a density of 5 × 10^5^ cells.

For IF analysis of MDCK cells (Transwells, coverslips), mCCD cells (Transwells), and Eph4 cells (coverslips), cells were washed with PBS containing Ca^2+^ and Mg^2+^ (PBS++), fixed in 1% PFA pre-warmed at 30°C for 12 min, followed by rinsing 2× with PBS++ and incubating with methanol (MeOH) at −20°C for 5 min, followed by gradual rehydration in PBS++ (3× replacing 50% of volume with PBS), and 2× washes in PBS++ (Dugina et al., 2009; Baranwal et al., 2012). Cells on coverslips or on filters were permeabilized with 0.2% of Triton X-100 in PBS++ (5 min at RT) and saturated 30 min with 2% of BSA in PBS++. For Fig. 8 A, MDCK cells were washed 2× with cold PBS, fixed in MeOH for 16 h at −20°C, followed by 1 min treatment with acetone (−20°C). Filters were rehydrated in IMF buffer (0.1% Triton X-100, 0.15 M NaCl, 5 mM EDTA, 20 mM Hepes, pH 7.5, 0.02% NaN_3_).

For Fig. 10, Eph4 cells were fixed with cold (−80°C) MeOH for 8 min at −20°C.

Incubation with primary antibodies was carried out for 16 h at 4°C or 2 h at RT in a humidified chamber, followed by washing 3× with PBS, incubation with secondary antibodies (1–2 h at RT), and washing. Filters were placed on glass slides (cells facing up) and mounted with Fluoromount-G. Coverslips were mounted in either Vectashield/DAPI or Fluoromount-G.

Slides were imaged on a Zeiss LSM800 confocal microscope using a Plan-Apochromat 63×/1.40 oil objective or a Plan-Apochromat 100×/1.40 oil objective (1,024×1,024 px). In alternative, we used an upright Leica DM4B microscope, using 63×/1.40 oil objective (2,048×2,048 px, pixel size = 0.10 μm). Either single confocal plane images (typically for cells grown on coverslips) or maximum intensity projections of z-stack images (typically 3–6 confocal planes over 1.0–1.5 µm, step size = 0.3–0.6 µm) for cells growing on Transwell filters were obtained. Images were extracted from .lif, .lsm, or .czi files using ImageJ, adjusted and cropped using Adobe Photoshop, and assembled in Adobe Illustrator figures.

### Plasmids

Constructs of HA-myc-tagged hZO-1 in pCDNA3.1(+) (Spadaro et al., 2017), myc-his-tagged msCGN in pCDNA3.1(−) and HA-tagged hCGN in pCDNA3.1(+) (Vasileva et al., 2022), GFP-myc-his in pCDNA3.1(−) (Guerrera et al., 2016), CFP-HA in pCDNA3.1(+) (Spadaro et al., 2014), GFP-myc in pTRE2Hyg (Paschoud et al., 2014), and YFP-myc in pTRE2Hyg (Paschoud et al., 2012) were described previously. mCherry-myc was a gift from the Picard laboratory (Department of Cell Biology, University of Geneva, Geneva, Switzerland). pET21c-hNM2A-1343–1965, pET21c-hNM2B-1337–1976, and pET21c-mNM2C-1297–2000 were a gift from the laboratory of Prof. S. Ravid (Hebrew University of Jerusalem, Jerusalem, Israel).

The following new constructs were generated by PCR amplification with appropriate oligonucleotides and subcloned into the indicated cloning sites. mCherry-Flag (S2429) KpnI-NotI in pCDNA3.1(−) GFP-cCGN-myc (1–1,190aa; S1115) and GFP-cCGN-myc-Δ1003-1190 (1–1,002aa; S2694) XbaI-KpnI and NotI-ClaI, respectively, in pCDNA3.1(−). GFP-mCGN-myc (1–1,192aa; S2363) NotI-ClaI in pTRE2Hyg. cCGN-myc (1–1,190aa; S2697) and cCGN-myc- Δ1003-1190 (1–1,002aa; S2695) BamHI-ClaI in pTRE2hyg. GFP-cCGNL1-myc (1–1,295aa; S1148) and GFP-cCGNL1-myc-Δ878-1295 (1–877aa; S2816) NotI-ClaI+AccI and SacII-XhoI, respectively, in pCDNA3.1(−). CFP-cCGNL1-HA (1–1,295aa; S1353) NotI-AceI+ClaI in pCDNA3.1(+). cCGNL1-myc (1–1,295aa; S2808) and cCGNL1-myc-Δ878-1295 (1–877aa; S2809) BamHI-ClaI in pTRE2hyg. GFP-mCGNL1-myc (1–1,298aa; S2799) and cCGNL1-myc-Δ881-1298 (1–880aa; S2815) EcoRI-NotI and EcoRV-XhoI, respectively, in pCDNA3.1(+). CGN-FL-10xHis-2xStrep (1–1,203aa; S2517) and hCGN-FL-10xHis-2xStrep (1–1,302aa; S2518) KpnI-XbaI in pACEBac1.

mNM2A, cNM2A, hNM2B, cNM2B, msNM2C, and cNM2C were synthesized by Genescript and the construct of HA-mNM2A (1–1,959aa; S2748), HA-mNM2A-Flag (1–1,960aa; S2782), HA-hNM2B (1–1,975aa; S2749), HA-cNM2B-1757–2006 (1–1,756; S2777), HA-cNM2B-Flag (1–2,006aa; S2747), and HA-cNM2C (1–2,065aa; S2804) were generated with BamHI-NotI sites of pcDNA3.1(+). mCherry-C1-mNM2C-mCherry (1–2,000aa; S2543) was a ready-to-use construct from Genescript.

Constructs of GST-tagged fragments of CGN and CGNL1 in pGEX4T1 (Guillemot et al., 2014), GST-tagged mZO1-C-terminal large (Vasileva et al., 2022), and GST-tagged central part of hPLEKHA7 (Pulimeno et al., 2011) were described previously. GST-tagged fragments of mCGN: 1–112aa (S2810) and hCGN: 230-353aa (S98), 1,161-1,203aa (S2666), 667-1,203aa (S2667), 667-1,160aa (S2668), and mCGNL1: 1–122aa (S2811) hCGNL1: 250–420aa (S1262), 421–603aa (S1020), 884–1,127 (S1251), 1,105–1,302 (S1252) were generated by PCR and subcloning into pGEX4T1 (EcoRI-XhoI, S1789, S97, S98; EcoRI-NotI, S2810, S2811, S1851; BamHI-EcoRI, S1024; EcoRI-SalI, S1262, S1251, S1252; BamHI-SalI, S1020; SalI-NotI, S2666, S2667, S2668). GST-tagged fragments of cNM2A, cNM2B, cNM2C all construct described in key resources table were generated by PCR and subcloning into pGEX4T1 with EcoRI-NotI, only GST-cNM2A (1,461–1,710; S2766) and GST-cNM2C (1,566–1,815; S2767) were generated with EcoRI-XhoI.

### Protein purification

The C-terminal coiled-coil fragments of hCGN (667–1203) hNM2A (1330–1960), hNM2B (1337–1976), and mNM2C (1287–2000) were purified as described in (Straussman et al., 2007). Briefly, bacteria were induced with 0.1 mM IPTG, lysed using a French press (three cycles at 1000 psi, SLM Instruments, Inc.) and clarified by centrifugation at 30,000 *g*, 45 min, 4°C (JA 30.5 rotor Beckmann coulter). The supernatant was incubated at 95°C for 15 min and cleared by centrifugation (45,000 rpm, Ti 70 rotor, Beckmann Coulter, 1 h at 4°C). The heat-stable soluble proteins were precipitated by slowly adding a saturated ammonium sulfate solution (1.5 ml per ml of supernatant), collected by centrifugation (20,000 *g*, 15 min, 4°C) and the pellet was dialyzed against Buffer G (20 mM Tris-HCl, pH 7.5, 600 mM NaCl, 5 mM EDTA, 1 mM DTT; overnight for 16 h at 4°C).

Full-length hCGN and hCGNL1 were purified from insect cell lysates lysed in 25 mM Hepes, pH 7.4, 300 mM NaCl, 1mM EDTA, 1 mM Benzamidine, 0.1% Triton, and 2 mM DTT, and the clarified lysate was purified by affinity chromatography using StrepTag resin.

For MST experiments, we used purified proteins. His-tagged hCGN Rod2 (667–1203) with either hNM2A Rod (1330–1960) or hNM2B Rod (1337–1976; purification as described above). Affinity-purified GST-tagged proteins (either mCGN [1–112] or mCGNL1 [1–122]) with affinity-purified His-tagged mZU5 (1520–1745). For the mCGN and mCGNL1 N-terminal fragments, affinity purification on glutathione Sepharose was preceded by denaturation of the bacterial pellet in 6 M guanidium hydrochloride, sonication, incubation on ice for 1 h, and renaturation by dropwise dilution into 10 volumes of renaturation buffer (25 mM Hepes, 200 mM NaCl, 10 mM DTT, and 1 mM EDTA, pH 7.4).

### GST pulldown

For GST pulldowns, GST-tagged protein baits (Table S1) were expressed in BL21 bacteria and purified by affinity chromatography on magnetic beads as described in (Sluysmans et al., 2021b). Preys were either purified full-length hCGN or hCGNL1, or purified rod fragments of hNM2A (1330–1960), hNM2B (1337–1976), mNM2C (1287–2000), or tagged (either GFP or HA) full-length and mutant proteins expressed in HEK293T cells. Pulldowns were carried out as described in (Sluysmans et al., 2021b).

### CGN and NM2B rod assembly and solubility assays

For the solubility assay, proteins were clarified by centrifugation (45,000 rpm for 25 min, TLA 100.3, Beckmann Coulter, 4°C). The samples (either NM2B Rod or CGN Rod2 or CGN Rod2+NM2B Rod) were diluted to 5 μM in buffer G and dialyzed overnight against 10 mM sodium phosphate, 150 mM NaCl, and 2 MgCl_2_ in mini GeBAflex dialysis tubes. The dialyzed proteins (100 μl) were centrifuged at 45,000 rpm for 1 h at 4°C to pellet filaments. Supernatant and pellet fractions were resuspended in an equal volume of buffer G, diluted with 5× SDS sample buffer and analyzed by SDS-PAGE. The intensity of the Coomassie-stained supernatant and pellet bands was measured using gel quantifier in Image J. The intensity values from the raw gel images were obtained from the area of the peaks selected using the first lane command. The soluble fraction of NM2B Rod was determined as a fraction (%) of total intensity (obtained by combining the intensities of supernatant and pellet).

### Negative staining electron microscopy

Samples after dialysis were diluted to 0.1 mg/ml, and 10 μl was placed on lacy carbon grids (300 mesh, 150 μm) for 1 min. The grids were washed twice with dialysis buffer (5 s each) and stained with 1% uranyl acetate for 1 min. The samples were washed, stained, and dried by blotting the grids with Whattman paper. Samples were imaged with a Talos L120C microscope (120 KeV, single tilt holder, Thermo Fisher Scientific). For quantification of filament length and width, we used the Line Profile command of ImageJ (*n* = 200), and statistical analysis was performed using Prism software (Mann–Whitney test).

### MST

His-tagged proteins (either hCGN Rod2 or mZU5) were labeled with the red tris-NTA dye using the monolith His-Tag labeling kit (Nanotemper Technologies). Briefly, equal volumes of protein (200 nM) and dye (100 nM) solutions were mixed and incubated for 30 min at RT. The labeled protein was clarified by centrifugation at 15,000 *g* for 10 min at 4°C before use. The buffer (1× PBS containing 0.05% Tween 20 [PBS-T]) used for labeling and subsequent binding assay was provided in the kit.

For MST experiments, 10 μl of labeled protein were mixed with 10 μl of interactor at the appropriate concentration range. For GST-mCGN (1–112) and GST-mCGNL1 (1–122; target: mZU5, 1520–1745) the range was 0–1,000 nM (in PBS-T buffer). For hNM2B Rod (1337–1976) and hNM2A Rod (1330–1960; target: hCGN Rod2, 667–1203), the range was 0–10,000 nM. Data were fitted using the Monolith NT.115 fitting algorithm (Nanotemper Technologies) to obtain a dissociation constant (K_d_).

### Transfection, siRNA-mediated depletion, and exogenous expression of proteins

For transfections (rescue and exogenous expression experiments), cells grown either on glass coverslips in 24-well plates seeded at a density of 1–2 × 10^5^ cells/well or on 24-mm Transwell filters seeded at a density of 5 × 10^5^ cells, transfected next day using jetOPTIMUS DNA transfection reagent according to the manufacturer’s protocol and fixed for IF 3 d (cells plates on glass coverslips) or 5 d (cells plates on Transwell filters) after transfection.

For siRNA-mediated PLEKHA7 and NM2C depletion (target sequences in Table S1) cells grown on glass coverslip in 24-well plates, transfected next days with Lipofectamine RNAiMAX and fixed for IF 3 d after transfection.

HEK cells were plated in 10-cm dish (2 × 10^6^ cells/dish), transfected next day using Lipofectamine 2000, and lysed 48 h after transfection.

Imaging settings and treatment were carried out as described in IF section.

### IB

Cell lysates were obtained using radioimmunoprecipitation assay buffer containing Pierce protease inhibitor. IB was performed as previously described (Spadaro et al., 2017; Vasileva et al., 2017). Samples (20 μg total protein) were separated by 8–15% SDS-PAGE, and β-tubulin was used for protein loading normalization. Numbers on the left of IB indicate migration of pre-stained molecular size markers (kD).

### AFM indentation measurements

The AFM-based indentation measurements were carried out using a commercial AFM (Dimension FastScan, Icon Scanner; Bruker). A polystyrene bead (5 µm radius; Invitrogen) was stuck on the tipless silicon nitride cantilever (MLCT-O10-E, Bruker) by epoxy fix. The spring constant of the home-made cantilevers, calibrated each time before measurement by thermal fluctuation method, was in the range of 0.10–0.15 N m^−1^.

All AFM indentation measurements were carried out in cell-culture medium at room temperature. The cells were cultured in 60 mm petri dish for 36 h in incubator until forming monolayers (confluency >80%). In a typical experiment, the cantilever was brought to the cell layer with the constant speed of 1 µm s^−1^ until reaching the maximum contact force of 5 nN, where the maximum indentation distance of cells was in the range of 0.5–1.5 µm. Then, the cantilever was retracted and moved to another spot for the next cycle. A box pattern containing 100 spots in 40 × 40 µm region was set and typically 5–10 such regions were randomly selected in each measurement to obtain the averaged stiffness of the cell.

The force-indentation traces were analyzed to obtain the Young’s modulus of the cells using the NanoScope Analysis program. After baseline correction and contact point estimation, the approaching force-indentation curve was fitted with the Hertz (Spherical) model (Eq. 1) in the contact force range from 0.5 to 4.5 nN. Constant parameters and data range were chosen to minimize the bias for different cell types (Harris and Charras, 2011)

$$F\left(x\right)=\frac{4Er^{1/2}}{3\left(1‐v^{2}\right)}x^{3/2},$$ (1)

where F is the force of the cantilever, x is the indentation distance of the cell pressed by the cantilever, E is the Young’s modulus of the cell layer, r is the radius of the spherical indenter, and v is the Poisson ratio. The Poisson ratio of cell is normally in the range of 0.3–0.5. We chose v = 0.5 in all calculations.

### In situ PLA

The PLA was conducted according to the manufacturer’s instructions (Sigma-Aldrich). In brief, after fixation with PFA and methanol, endogenous ZO-2, γ-actin, β-actin, and PLEKHA7 were detected using rabbit anti–ZO-2, mouse anti–γ-actin, mouse anti–β-actin (Dugina et al., 2009), and guinea pig anti-PLEKHA7, respectively. PLA probes anti-Rabbit MINUS and anti-Mouse PLUS were used, as well as Detection Reagent Green. PLA dots were acquired with an LSM 800 Zeiss confocal microscope. Quantifications were performed with ICY software (spot detector function; http://icy.bioimageanalysis.org). Two regions of interest (ROI) were drawn by hand: one all around the cell (ROI-Cell) and another in the cytoplasmic part (ROI-Cyto). To get PLA dots just at the junctional level, we subtracted the number of PLA dots in ROI-Cell by the number of PLA dots in ROI-Cyto. PLEKHA7 labeling was performed during the last washes of PLA. Alexa Fluor 647 anti–guinea pig secondary antibodies were diluted in PLA Wash Buffer B and incubated for 20 min at RT.

### STED microscopy

The endogenous ZO-1, CGN, and NM2B were detected using rat anti–ZO-1, mouse anti-CGN, and rabbit anti-NM2B, respectively. Alexa Fluor 488 anti-rat, Abberior STAR 580 anti-mouse, and Abberior STAR Red anti-rabbit were used as secondary antibodies.

2D-STED imaging was performed with a Leica TCS SP8 STED 3X microscope in a thermostated chamber at 21°C and equipped with a STED motorized oil immersion objective (HC PL Apo 100×/N.A. 1.40 CS2). Fluorescently labeled samples were mounted in Prolong Antifade Gold (Thermo Fisher Scientific) between a coverslip (0.170 ±0.01 mm thick, Hecht-Assistent) sealed on a microscope slide with nail polish. Excitation was performed with a White Light Laser (WLL), depletion with either a continuous 592 nm laser (STED 592) or a 775-nm pulsed laser (STED 775). Excitation and depletion lasers were calibrated with the STED Auto Beam Alignment tool during imaging sessions (Leica LAS X software, Leica Microsystems CMS GmbH).

2D-STED was made using an excitation at 587 nm (WLL) and a STED 775 depletion laser line for STAR 580 (CGN), an excitation at 638 nm (WLL) and a STED 775 depletion laser line for STAR Red (NM2B), followed by an excitation at 488 nm (WLL) and a STED 592 depletion laser line for Alexa Fluor 488 (ZO-1). Detection signals were collected from 597 to 630 nm for STAR 580, from 648 to 690 nm for STAR Redm and from 498 to 540 nm for Alexa Fluor 488 using highly sensitive Leica Hybrid Detectors with a fixed gain and offset (100 mV and 0, respectively). Time-gated detection was used for all channels (0.3–6 ns). Acquisitions were performed sequentially with a line average of 4 and an optimized pixel size. Images were deconvolved using the Leica Lightning Mode and analyzed with ImageJ software. Linescans and distances from midline were determined using ImageJ software (plot profile function; http://rsbweb.nih.gov/ij/).

### Ultra-expansion microscopy

Cells were cultured on glass coverslips in 24-well plates for 3 d seeded at a density of 1–2 × 10^5^ cells/well. Upon confluency, drugs were added to the medium: blebbistatin (50 μM, 4 h), Y27632 (10 μM, 4 h), SMIFH2 (50 μM, 4 h), CK-869 (100 μM, 4 h), and DMSO (negative control). Fixation and subsequent immunofluorescence are carried out after incubation with the drugs, using the protocol described in Gambarotto et al. (2019). Cells were fixed with cold (−80°C) methanol for 8 min at −20°C.

### RNA sequencing (RNASeq) analysis of CGN and CGNL1 mRNA levels

For RNA extraction, 3 × 10^5^ MDCKII cells were cultured into 6-well plates for 3 d, washed with PBS, trypsinized, and pelleted at 5,000 g for 5 min. After removing the supernatant, RNA was extracted using the NucleoSpin RNA kit (cat: 740955.50; Macherey-Nagel). RNA quantification was performed with a Qubit fluorimeter (Thermo Fisher Scientific) and RNA integrity assessed with a Bioanalyzer (Agilent Technologies). The TruSeq mRNA stranded kit from Illumina was used for the library preparation with 500 ng of total RNA as input. Library molarity and quality were assessed with the Qubit and Tapestation (DNA High sensitivity chip). Libraries were sequenced on a Hiseq 4000 Illumina sequencer with an average of 25 million of SR100 reads per sample. To map and quantify differential gene expression, the reads were aligned with STAR v.2.7.0 to the NCBI canis lupus familiaris canFam3 genome (NC_006583.3). The gene expression was quantified with HTSeq v.0.9.1. The differential expression analysis was performed with R/Bioconductor edgeR package (Anders et al., 2015). The counts were normalized according to the library size and filtered. The genes having a count above 1 count per million reads in at least three samples were kept for the analysis. The differentially expressed genes tests were done with a general linear model using a negative binomial distribution. The genes were considered as differentially expressed when the fold change was at least twofold with a 5% false discovery rate Benjamini-Hochberg multiple testing correction.

### Prediction of molecular assembly of rod domains

The structure and axial assembly of α-fibrous proteins was studied using a customized in-house suite of programs called AASAP (Amino Acid Sequence Analysis Program). This includes a fast Fourier transform technique to delineate sequence periodicities, routines to predict likely secondary conformation and flexibility variations along the length of the molecules, and an assembly routine that considers all possible modes of molecular alignment in terms of the numbers of intermolecular apolar and ionic interactions that could be made in silico. Here, the latter was employed to determine the most likely modes of molecular assembly of the linear coiled-coil structures of CGN, CGNL1, NM2A, NM2B, and NM2C. The conclusions were based on the modes of assembly that led to the maximum number of intermolecular interactions being made (Hulmes et al., 1973; Parry et al., 1977; Fraser et al., 1986). Specifically, one molecule was computationally slid past a second in both parallel and antiparallel orientations and the total numbers of apolar and ionic interactions were determined as a function of relative axial stagger between the molecules. An apolar interaction is defined as the state where an apolar residue in the first molecule lies within plus or minus one residue axially of another apolar residue in the second molecule. Likewise, an ionic interaction is said to occur when a charged residue in one molecule (aspartic acid, glutamic acid, arginine, and lysine) lies axially within plus or minus two residues of one with an opposite charge in the second molecule. The peaks in the interaction distributions are postulated to correspond to the most likely modes of molecular assembly (Hulmes et al., 1973; Parry et al., 1977; Fraser et al., 1986). A normalized cut-off score of 0.23 was arbitrarily selected to highlight only the most significant peaks in the interaction distribution. These calculations were combined for the relevant proteins of both human and dog. When both molecules have similar direction (i.e., parallel), the stagger is defined as the number of residues in a coiled-coil conformation between the N-terminus of CGN (or CGNL) and the N-terminus of NM2A, NM2B, or NM2C. For an antiparallel arrangement, the stagger is defined between the N-terminus of CGN/CGNL and the C-terminus of the NM2 molecules.

### Quantification and statistical analysis

Data processing and analysis were performed in GraphPad Prism 8. All experiments were carried out at least in duplicate, and data are shown either as dot plots, as histograms, or as line graph (with mean and SD indicated). Statistical significance was determined by unpaired Mann–Whitney’s test (when comparing two sets of data), or unpaired *t* test, or Kruskal–Wallis test followed by Dunn’s multiple comparison, as detailed in the figure legends (ns = not significant difference, *P ≤ 0.5, **P ≤ 0.01, ***P ≤ 0.001, ****P ≤ 0.0001).

### Analysis of immunofluorescence data

For the quantification of junctional immunofluorescent signal pixel intensity for each channel was measured in the selected junctional area using the polyhedral tool of ImageJ, and the averaged background signal of the image was subtracted. Relative intensity signal was expressed as a ratio between the signal of protein of interest and an internal junctional reference (either ZO-2, or PLEKHA7 or occludin, or PLEKHA6). Typically 30–70 junctional segments were analyzed, for each of independent duplicate or triplicate experiments.

For the analysis of distances of TJ proteins and their N- and C-termini, slides were imaged on a Zeiss LSM800 confocal microscope using a Plan-Apochromat 100×/1.40 oil objective at a resolution of 1,024×1,024 px. Linescan-Analysis (ImageJ) was carried out on a 1-μm linear distance across the junction, centering on the maximum intensity signal. Using the Plot-Profile plugin, ImageJ pixel intensities (y axis) of red, green, and far-red fluorophores were plotted as a function of distance (x axis) across the junction. The x-coordinate of the maximum intensity peak was determined using the mean function of the Gaussian curve (Prism software).

For the measurement of the ZI (L[TJ]/L[St]; ratio between actual length of bicellular junction and the distance between two vertexes), we used the method described in Tokuda et al. (2014), and measured the length of the TJ (L[TJ]) using the freehand line trace in ImageJ, and the straight length of junction (L[St]) using a straight line between vertexes. Typically between 40 and 200 bicellular junctions were analyzed.

For the quantification of PLEKHA7 puncta, the length of the junction was measured using the Straight Line tool of Fiji/ImageJ. For each junction, PLEKHA7 puncta were counted (defined as distinct individual immunofluorescent puncta along a junction marked with ZO-1) and divided by a tenth of the junction length in order to plot the number of puncta for every 10 µm of junction. Between 60 and 130 junctional segments were analyzed across at least three experiments, each segment being used as a replicate. For the quantification of inter-puncta distance in conventional images, the Straight Line tool of Fiji/ImageJ was used to draw a line along junctions containing PLEKHA7 puncta, and the Plot Profile command was applied. Distance between peaks of PLEKHA7 immunofluorescent signal was plotted as replicates. For the quantification of inter-puncta distance, gap, and puncta length in U-ExM images, the Straight Line tool of Fiji/ImageJ to measure the different distances as depicted in Fig. 10 E. Each measure was used as a replicate. Each dot of dot plot graphs represents one measurement, and data are shown in arbitrary units (a.u.).

### Resource availability

#### Lead contact

Further information and requests for resources and reagents should be directed to and will be fulfilled by the lead contact, Sandra Citi (sandra.citi@unige.ch).

#### Materials availability

Reagents generated in this study will be made available on request. We may require a payment for shipping and a completed Materials Transfer Agreement.

### Online supplemental material

Fig. S1 (related to Fig. 1) shows in vitro interaction of CGN and CGNL1 with NM2A, NM2B, and NM2C. Fig. S2 (related to Fig. 3) shows the specificity of anti-NM2 antibodies and the role of CGN in the regulation of NM2s, MgcRacGAP, and GEF-H1 in cells. Fig. S3 (related to Fig. 4) shows that the junctional accumulation of NM2C is not promoted by CGN and CGNL1. Fig. S4 (related to Fig. 5) shows the mapping of antibody-binding regions and the calculated distances between CGN and CGNL1 and ZO proteins. Fig. S5 (related to Fig. 5) shows that CGNL1 does not link ZO-1 to NM2B in vitro and binds to the ZU5 domain of ZO-1 with 10-fold lower affinity than CGN. Fig. S6 (related to Fig. 7) shows that cingulin regulates actin filament organization at tight junctions of mCCD and Eph4 cells. Fig. S7 (related to Figs. 8 and 9) shows that TJ membrane tortuosity is regulated by CGN interaction with NM2B. Table S1 lists resources.

## Supplementary Material

Table S1 is a resources table.

SourceData F1 is the source file for Fig. 1.

SourceData F2 is the source file for Fig. 2.

SourceData F5 is the source file for Fig. 5.

SourceData FS1 is the source file for Fig. S1.

SourceData FS2 is the source file for Fig. S2.

SourceData FS3 is the source file for Fig. S3.

SourceData FS4 is the source file for Fig. S4.

SourceData FS5 is the source file for Fig. S5.

SourceData FS6 is the source file for Fig. S6.

SourceData FS7 is the source file for Fig. S7.

## Data Availability

The RNASeq data obtained in this study have been deposited at the NCBI GEO under accession number GSE228955. The key data are available in the article itself and its supplementary materials. Data for additional experiments used for statistical analysis and quantifications will be deposited in a public archive in the future and are available upon reasonable request from the lead contact, Sandra Citi (sandra.citi@unige.ch).
