# Supplementary material for: Cingulin and paracingulin tether myosins-2 to junctions to mechanoregulate the plasma membrane
Source: J Cell Biol. 2023 May 19;222(7):e202208065. doi: 10.1083/jcb.202208065 (PMC10202830; doi:10.1083/jcb.202208065)
Supplement: Table S1 — is a resources table. [file JCB_202208065_TableS1.docx]

**Table S1.** Resources Table.

| **REAGENT or RESOURCE** | **SOURCE** | **IDENTIFIER** |
| --- | --- | --- |
| Antibodies | | |
| Mouse monoclonal anti-GFP (IB, IF) | Roche | Cat# 11814460001, RRID:AB_390913 |
| Rabbit polyclonal anti-GFP (IF) | Thermo Fisher Scientific | Cat# A-11122, RRID:AB_221569 |
| Mouse monoclonal anti-HA (IB) | Thermo Fisher Scientific | Cat# 32-6700, RRID:AB_2533092 |
| Rabbit polyclonal anti-HA (IF) | Santa Cruz | Cat# sc-805, RRID:AB_631618 |
| Mouse monoclonal anti-myc (IF) | Gerard Evan, MRC | Cat# 9E10, RRID:AB_2266850 |
| Mouse monoclonal anti-Flag (IB) | Sigma-Aldrich/Merck | Cat# F1804  RRID:AB_262044 |
| Mouse monoclonal anti-His (IB) | BD Biosciences | Cat# 552565  RRID:AB_394432 |
| Rabbit polyclonal anti-cingulin (IB, IF) | Citilab | C532 |
| Mouse monoclonal anti-cingulin (IB, IF) | Citilab | 22BD5A1 |
| Rabbit polyclonal anti-paracingulin (IB) | Citilab | 20893 |
| Mouse monoclonal anti-paracingulin (IB) | Santa Cruz | Cat# sc-377525 |
| Rat monoclonal anti-ZO-1 (IB, IF) | D. Goodenough, Harvard Medical School | R40.76, RRID:AB_2205518 |
| Guinea pig polyclonal anti-PLEKHA7 (IF) | Citilab | GP2737 |
| Rat polyclonal anti-PLEKHA6 (IF) | (Sluysmans et al., 2021a) | RtSZR127 |
| Goat polyclonal anti-ZO-2 (IF) | Santa Cruz | Cat# sc-8148, RRID:AB_2271821 |
| Rabbit polyclonal anti-ZO-3 (IB, IF) | Thermo Fisher Scientific | Cat# 36-4100, RRID:AB_148475 |
| Mouse monoclonal anti-occludin (IF) | Thermo Fisher Scientific | Cat# 33-1500, RRID:AB_87033 |
| Mouse monoclonal anti-E-cadherin (IF) | BD Biosciences | Cat# BD 610181  RRID:AB_397580 |
| Mouse monoclonal anti-β-tubulin (IB) | Thermo Fisher Scientific | Cat# 32-2600, RRID:AB_2533072 |
| Rabbit polyclonal anti-NMIIA (IB, IF) | Biolegend | Cat# 909801 RRID:AB_291638 |
| Rabbit polyclonal anti-NMIIB (IB, IF) | Biolegend | Cat# 909901 RRID:AB_291639 |
| Rabbit polyclonal anti-NMIIC (IB) | Covalab | Cat# 00111015  RRID:AB_not available |
| Rabbit polyclonal anti-NMIIC (IB) | Proteintech | Cat# 20716-1-AP  RRID:AB_10859247 |
| Rabbit polyclonal anti-NMIIC (IF) | Cell Signaling Technology | Cat# 8189  RRID:AB _10886923 |
| Mouse monoclonal anti-β-actin (IB, IF) | C. Chaponnier, University of Geneva | RRID:AB_2571580 |
| Mouse monoclonal anti-γ-actin (IB, IF) | C. Chaponnier, University of Geneva | RRID:AB_2571583 |
| Rabbit polyclonal anti-MLCII (IB) | Cell Signaling Technology | Cat#3672  RRID:AB_10692513 |
| Mouse monoclonal anti-P-MLCII (Ser19) (IF, IB) | Cell Signaling Technology | Cat#3675  RRID:AB_2250969 |
| Rabbit polyclonal PP-MLCII (Thr18/Ser19) (IB) | Cell Signaling Technology | Cat#3674  RRID:AB_2147464 |
| Alexa Fluor 488-AffiniPure Donkey Anti-Rabbit IgG | Jackson Laboratory | Cat# 711-545-152, RRID:AB_2313584 |
| Alexa Fluor 488-AffiniPure Donkey Anti-Mouse IgG | Jackson Laboratory | Cat# 715-546-150, RRID: AB_2340849 |
| Cy3-AffiniPure Donkey Anti-Rabbit IgG | Jackson Laboratory | Cat# 711-165-152, RRID:AB_2307443 |
| Cy3-AffiniPure Donkey Anti-Mouse IgG | Jackson Laboratory | Cat# 715-165-151, RRID:AB_2315777 |
| Cy3-AffiniPure Donkey Anti-Rat IgG | Jackson Laboratory | Cat# 712-166-150, RRID:AB_2340668 |
| Cy3-AffiniPure Donkey Anti-Goat IgG | Jackson Laboratory | Cat# 705-166-147, RRID:AB_2340413 |
| FITC-phalloidin | Sigma | Cat# P5282 |
| Rhodamine-phalloidin | Thermo Fisher Scientific | Cat# R415, RRID:AB_2572408 |
| Alexa Fluor 647-AffiniPure Donkey Anti-Guinea Pig IgG | Jackson Laboratory | Cat# 706-605-148,  RRID:AB_2340476 |
| Alexa Fluor 647-AffiniPure Donkey Anti-Rabbit IgG | Jackson Laboratory | Cat# 711-605-152,  RRID:AB_2492288 |
| Alexa Fluor 647-AffiniPure Donkey Anti-Goat IgG | Jackson Laboratory | Cat# 705-606-147,  RRID:AB_2340438 |
| Cy5-AffiniPure Donkey Anti-Mouse IgG | Jackson Laboratory | Cat# 715-175-150, RRID:AB_2340819 |
| Cy5-AffiniPure Donkey Anti-Rat IgG | Jackson Laboratory | Cat# 712-175-153, RRID:AB_2340672 |
| Abberior STAR 580 Goat Anti-Mouse IgG | Abberior | Cat# 2-0002-005-1  RRID: AB_2620153 |
| Abberior STAR Red Goat Anti-Rabbit IgG | Abberior | Cat# 2-0012-011-9  RRID:AB_2620152 |
| Anti-Mouse IgG (H+L), HRP Conjugate | Promega | Cat# W4021, RRID:AB_430834 |
| Anti-Rabbit IgG (H+L), HRP Conjugate | Promega | Cat# W4011, RRID:AB_430833 |
| Anti-Rat IgG (H+L), HRP Conjugate | Thermo Fisher Scientific | Cat# 62-9520,  RRID: AB_2533965 |
| Anti-Goat IgG (H+L), HRP Conjugate | Promega | Cat# V8051, RRID:AB_430838 |
| Duolink in situ PLA probe anti-rabbit MINUS | Sigma | DUO92005-100RXN |
| Duolink in situ PLA probe anti-mouse PLUS | Sigma | DUO92001-100RXN |
| Duolink in situ detection reagents green | Sigma | DUO92014-100RXN |
| Plasmids | | |
| **CGN** |  |  |
| pCDNA3.1(-)- mCGN-myc-his | (Vasileva et al., 2022) | S2407 |
| pCDNA3.1(+)- hCGN-HA | (Vasileva et al., 2022) | S2411 |
| pCDNA3.1(-)-GFP-cCGN-myc | This paper | S1115 |
| pCDNA3.1(-)-GFP-cCGN-Δ1003-1190-myc | This paper | S2694 |
| pTRE2Hyg-GFP-mCGN-myc | This paper | S2363 |
| pTRE2Hyg-cCGN-Myc | This paper | S2697 |
| pTRE2Hyg-cCGN- Δ 1003-1190-Myc | This paper | S2695 |
| pET21b-hCGN-667-1203-6His | This paper | S2764 |
| pACEBac1-10xHis-2xStrep-hCGN-FL | This paper | S2517 |
| **CGNL1** |  |  |
| pCDNA3.1(-)-GFP-cCGNL1-Myc | This paper | S1148 |
| pCDNA3.1(-)-GFP-cCGNL1-Δ878-1295-Myc | This paper | S2816 |
| pCDNA3.1(+)- CFP-cCGNL1-HA | This paper | S1353 |
| pTRE2Hyg-cCGNL1-Myc | This paper | S2808 |
| pTRE2Hyg-cCGNL1- Δ 878-1295-Myc | This paper | S2809 |
| pCDNA3.1(+)-GFP-msCGNL1-Myc | This paper | S2799 |
| pCDNA3.1(+)-GFP-msCGNL1- Δ 881-1298-Myc | This paper | S2815 |
| pACEBac1-10xHis-2xStrep-hCGNL1-FL | This paper | S2518 |
| **NM2** |  |  |
| pCDNA3.1(+)-HA-msNM2A | This paper | S2748 |
| pCDNA3.1(+)-HA-cNM2A-Flag | This paper | S2782 |
| pCDNA3.1(+)-HA-hNM2B | This paper | S2749 |
| pCDNA3.1(+)-HA-cNM2B-Flag | This paper | S2747 |
| pCDNA3.1(+)-HA-cNM2B- Δ 1757-2006 | This paper | S2777 |
| pCDNA3.1(+)-HA-cNM2C | This paper | S2804 |
| pET21c-hNM2A-1343-1965 | S. Ravid, Hebrew University | S2732 |
| pET21c-hNM2B-1337-1976 | S. Ravid, Hebrew University | S2730 |
| pET21c-mNM2C-1297-2000 | S. Ravid, Hebrew University | S2733 |
| **ZO-1** |  |  |
| pCDNA3.1(+)- myc-hZO-1-FL-HA | (Spadaro et al., 2017) | S1947 |
| **Control constructs** |  |  |
| pCDNA3.1(-)- GFP-myc-his | (Guerrera et al., 2016) | S1166 |
| pCDNA3.1(+)- CFP-HA | (Spadaro et al., 2014) | S1150 |
| pTRE2Hyg-mCherry-myc | D. Picard, University of Geneva | S2633 |
| pCDNA3.1(-)-mCherry-Flag | This paper | S2429 |
| pTRE2Hyg-GFP-myc | (Paschoud et al., 2014) | S1210 |
| pTRE2Hyg-YFP-myc | (Paschoud et al., 2012) | S1152 |
| Recombinant Proteins | | |
| **ZO-1** |  |  |
| GST-mZO-1-Cter large (1520-1745) | (Vasileva et al., 2022) | S2511 |
| GST-mZO-1-Cter large-His (1520-1745) | This paper | S2794 |
| **PLEKHA7** |  |  |
| GST-hPLEKHA7(351-820) | (Pulimeno et al., 2011) | S1193 |
| **CGN** |  |  |
| GST-mCGN (1-112) | This paper | S2810 |
| GST-hCGN(1-226) | (Guillemot et al., 2014) | S605 |
| GST-hCGN(230-353) | This paper | S98 |
| GST-hCGN(355-579) | (Guillemot et al., 2014) | S739 |
| GST-hCGN(571-794) | (Guillemot et al., 2014) | S740 |
| GST-hCGN(782-1025) | (Guillemot et al., 2014) | S741 |
| GST-hCGN(1015-1203) | (Guillemot et al., 2014) | S742 |
| GST-hCGN(1161-1203) | This paper | S2666 |
| GST-hCGN(667-1203) | This paper | S2667 |
| **CGNL1** |  |  |
| GST-mCGNL1 (1-122) | This paper | S2811 |
| GST-hCGNL1(1-250) | (Guillemot et al., 2014) | S1023 |
| GST-hCGNL1(250-420) | This paper | S1262 |
| GST-hCGNL1(421-603) | This paper | S1020 |
| GST-hCGNL1(591-882) | (Guillemot et al., 2014) | S821 |
| GST-hCGNL1(884-1127) | This paper | S1251 |
| GST-hCGNL1(1105-1302) | This paper | S1252 |
| GST-hCGNL1(884-1302) | (Guillemot et al., 2014) | S1260 |
| **NM2A** |  |  |
| GST-cNM2A (837-1185) | This paper | S2758 |
| GST-cNM2A (1186-1578) | This paper | S2759 |
| GST-cNM2A (837-1578) | This paper | S2778 |
| GST-cNM2A (1461-1960) | This paper | S2737 |
| GST-cNM2A (1461-1710) | This paper | S2766 |
| GST-cNM2A (1711-1960) | This paper | S2738 |
| GST-cNM2A (1861-1960) | This paper | S2739 |
| **NM2B** |  |  |
| GST-cNM2B (875-1222) | This paper | S2760 |
| GST-cNM2B (1223-1615) | This paper | S2761 |
| GST-cNM2B (875-1615) | This paper | S2779 |
| GST-cNM2B (1507-2006) | This paper | S2734 |
| GST-cNM2B (1757-2006) | This paper | S2735 |
| GST-cNM2B (1906-2006) | This paper | S2736 |
| **NM2C** |  |  |
| GST-cNM2C (932-1279) | This paper | S2762 |
| GST-cNM2C (1280-1672) | This paper | S2763 |
| GST-cNM2C (932-1672) | This paper | S2780 |
| GST-cNM2C (1566-2065) | This paper | S2740 |
| GST-cNM2C (1566-1815) | This paper | S2767 |
| GST-cNM2C (1816-2065) | This paper | S2741 |
| GST-cNM2C (1966-2065) | This paper | S2742 |
| Chemicals, Peptides, Miscellaneous | | |
| Blebbistatin | Sigma-Aldrich | Cat# B0560 |
| Y27632 | LKT laboratories | Cat# Y1000 |
| SMIFH2 | Sigma-Aldrich | Cat# S4826 |
| CK-869 | Sigma-Aldrich | Cat# C9124 |
| Hygromycin B Gold | InvivoGen | Cat# ant-hg-2 |
| Molecular Weight Markers for SDS-PAGE | BioRad | Cat# 1610373 |
| Pierce Protease Inhibitor Tablet, EDTA-free | ThermoScientific | Cat# A32965 |
| Dialysis tubing (mini GeBAflex tubes), catalog no. | Sigma-Aldrich | Cat# DO70-6-50 |
| Electron microscopy grids | Elecron Microscopy Sciences | Cat# LC300-CU-150 |
| Critical Commercial Assays | | |
| Lipofectamine RNAiMAX | Invitrogen | Cat# 13778030 |
| jetOPTIMUS | Polyplus | Cat# 117-15 |
| Q5 High fidelity Polymerase | NEB | Cat# M0491L |
| T4 DNA Ligase | Promega | Cat# M1801 |
| Experimental Models: Cell Lines | | |
| Mouse mammary epithelial cell line Eph4 WT | E. Reichmann, University of Zürich | (Fialka et al., 1996) |
| Mouse mammary epithelial cell line Eph4 ZO-1-KO | Tsukita Laboratory | (Umeda et al., 2004) |
| Mouse mammary epithelial cell line Eph4 CGN-KO | (Vasileva et al., 2022) | N/A |
| Mouse mammary epithelial cell line Eph4 CGNL1-KO | (Vasileva et al., 2022) | N/A |
| Human embryonic kidney HEK 293T | ATCC | N/A |
| Mouse Cortical Collecting Duct Cell Line (mCCD) | E. Feraille, University of Geneva | (Wang et al., 2014) |
| Mouse Cortical Collecting Duct Cell Line (mCCD) CGN-KO | (Vasileva et al., 2022) | N/A |
| Mouse Cortical Collecting Duct Cell Line (mCCD) CGNL1-KO | (Vasileva et al., 2022) | N/A |
| MDCKII (Madin–Darby Canine Kidney) Tet‐off | A Fanning, University of North Carolina | Clontech |
| MDCKII (Madin–Darby Canine Kidney) Tet‐off CGN-KO | (Vasileva et al., 2022) | N/A |
| MDCKII (Madin–Darby Canine Kidney) Tet‐off CGNL1-KO | (Vasileva et al., 2022) | N/A |
| MDCKII (Madin–Darby Canine Kidney) Tet‐off CGNL1-KO-YFP-myc | (Vasileva et al., 2022) | N/A |
| MDCKII (Madin–Darby Canine Kidney) Tet‐off CGN/CGNL1-double-KO | (Vasileva et al., 2022) | N/A |
| Experimental Models: Organisms/Strains | | |
| BL21 Competent cells | NEB | Cat# C2530H |
| DH5 alpha Competent cells | Thermo Fisher | Cat# 18265017 |
| DH10B Competent cells | Thermo Fisher | Cat# 18297010 |
| Oligonucleotides | | |
| siRNA target sequence: mouse PLEKHA7 CTCTCAAGGAGAATAAAGA | Sigma-Aldrich | N/A |
| siRNA target sequence: canis NM2C (MYH14)  n. 1: GTGAAGATCAGTCCATTCTCT  n. 2: GCCCTGCATTGACCTCATTGA | Sigma-Alrich | N/A |
| siRNA negative control | Sigma-Aldrich | Cat# N. SIC001 |
| Software and Algorithms | | |
| Image J | N/A | imagej.nih.gov/ij/  RRID:SCR_003070 |
| Adobe Photoshop | N/A | adobe.com  RRID:SCR_014199 |
| Adobe Illustrator | N/A | <http://www.adobe.com>  RRID:SCR_010279 |
| Prism GraphPad | N/A | <https://www.graphpad.com/scientific-software/prism/>  RRID:SCR_002798 |
| Icy | N/A | <http://icy.bioimageanalysis.org>  RRID:SCR_010587 |
| Snapgene Version 3.1.2 | N/A | snapgene.com  RRID:SCR_015052 |
