# Supplementary figures and images for: Cingulin and paracingulin tether myosins-2 to junctions to mechanoregulate the plasma membrane
Source: J Cell Biol. 2023 May 19;222(7):e202208065. doi: 10.1083/jcb.202208065 (PMC10202830; doi:10.1083/jcb.202208065)

**C**

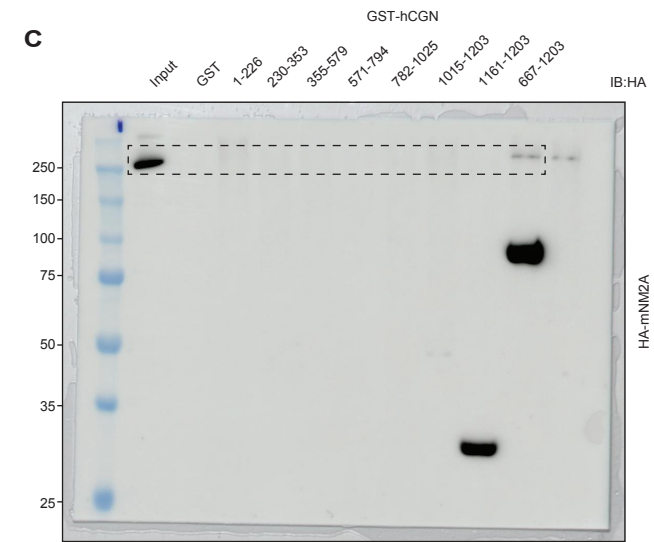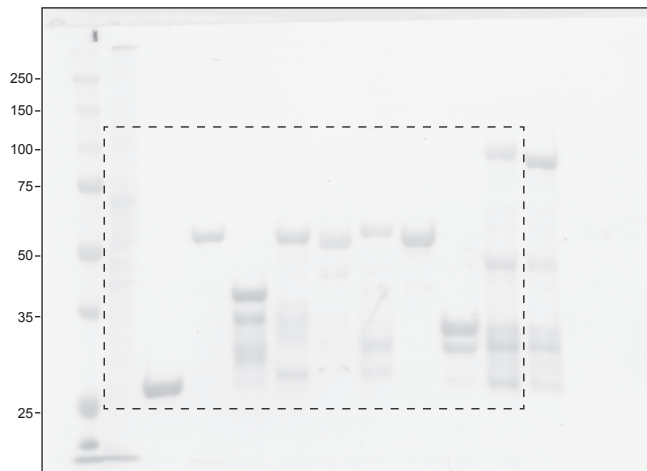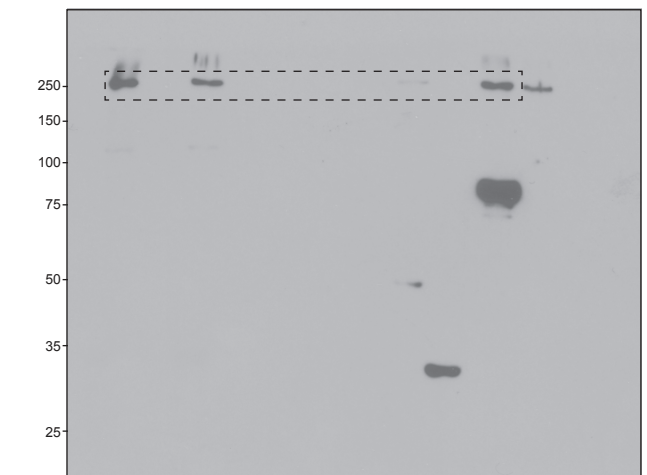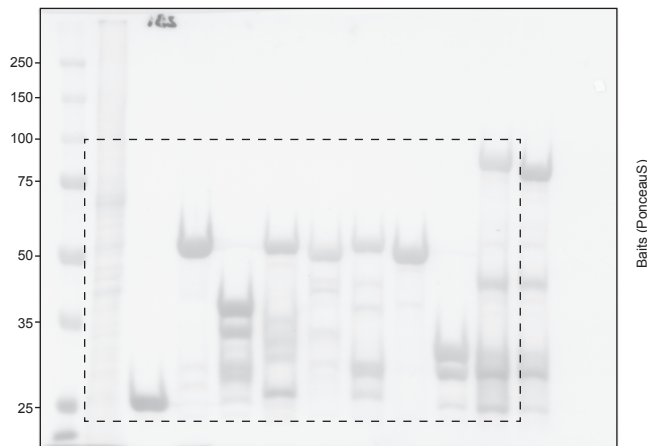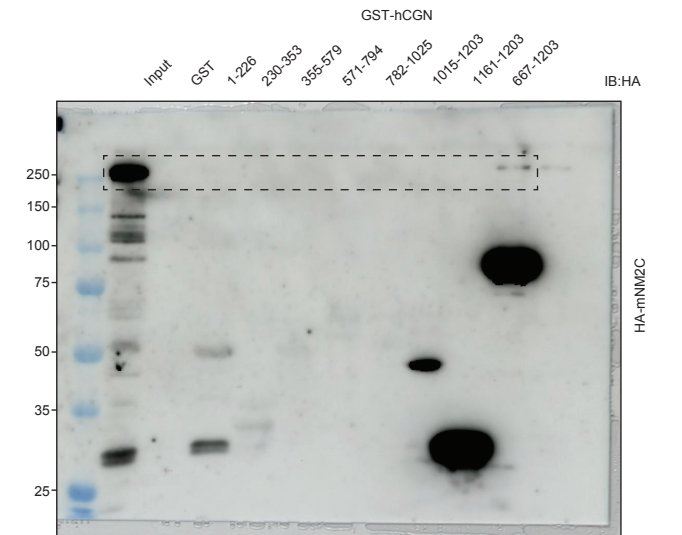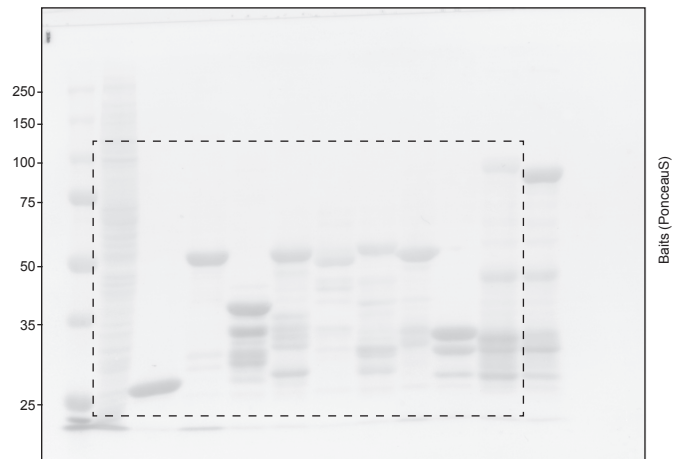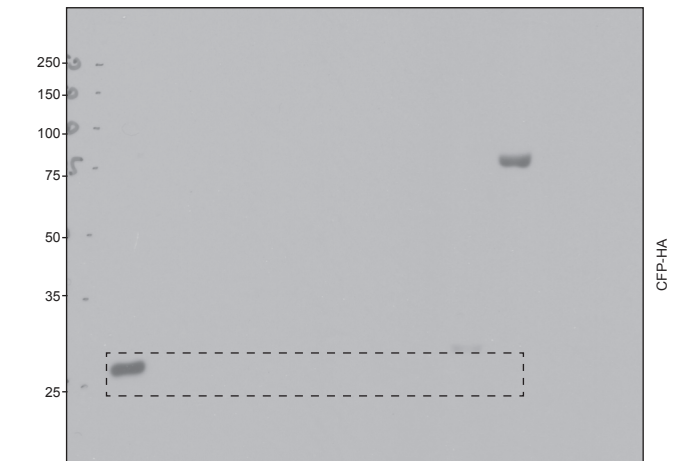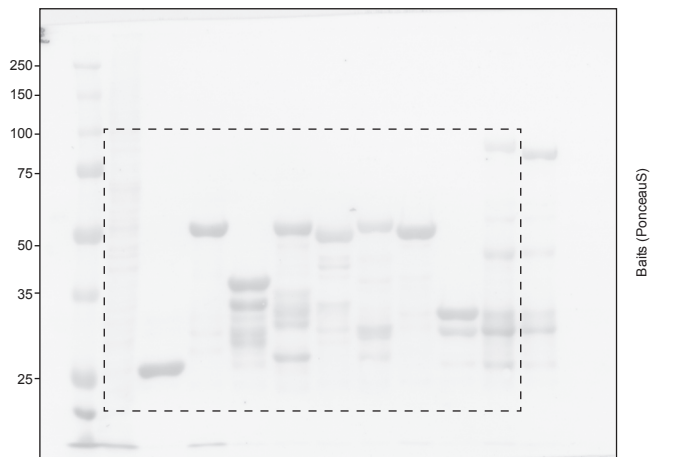

**D**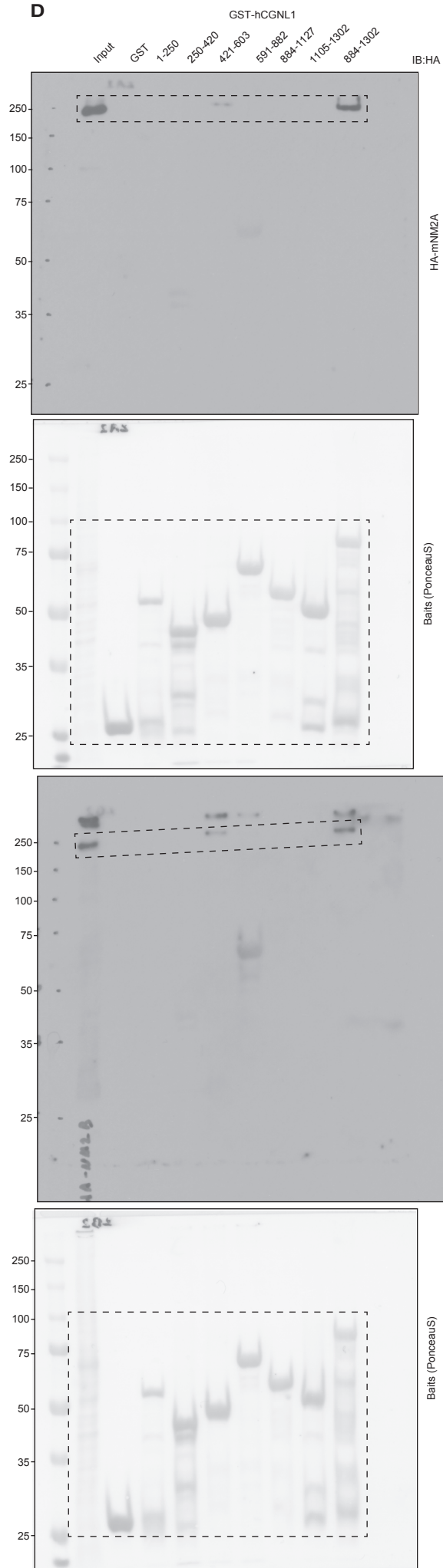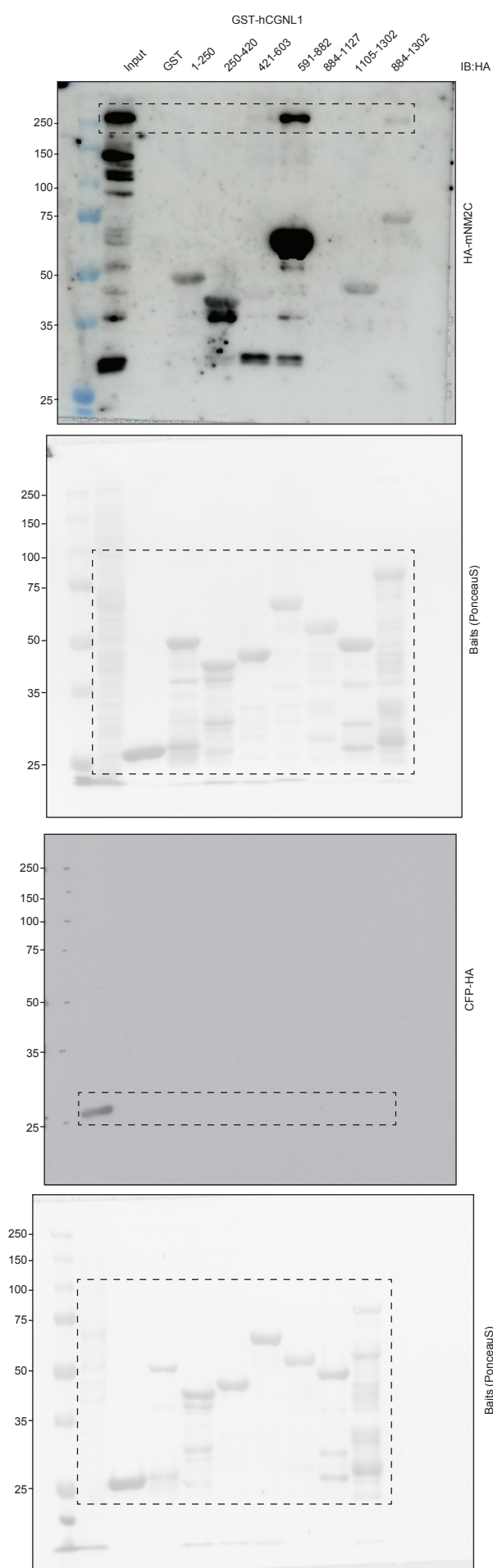

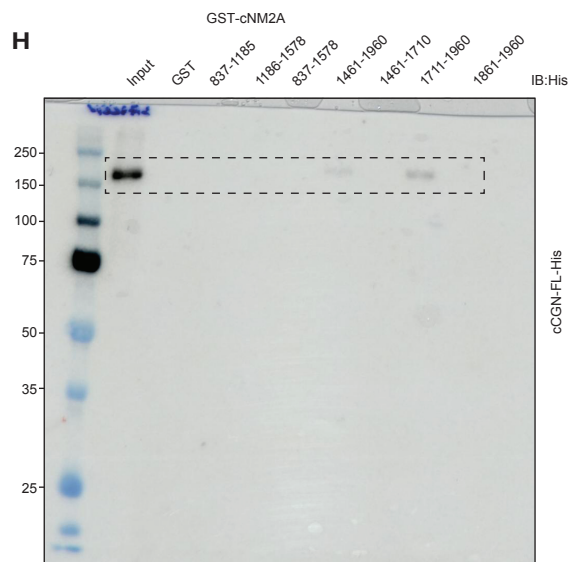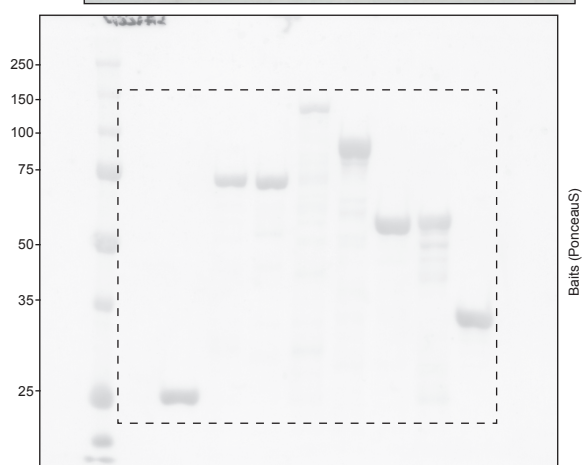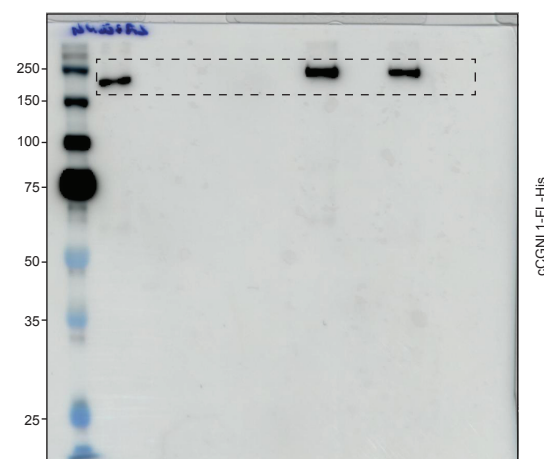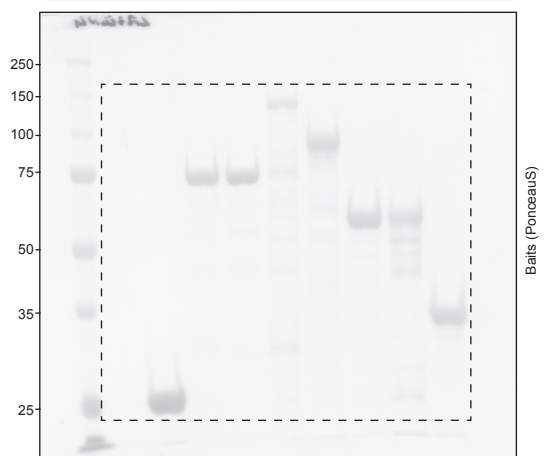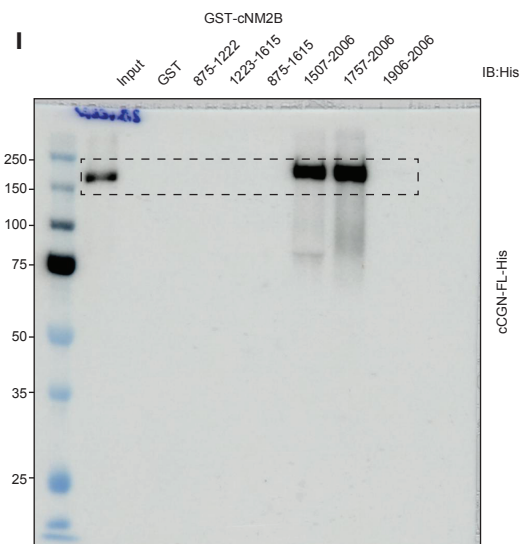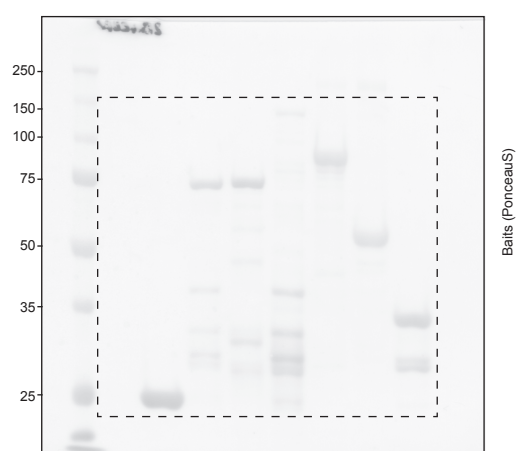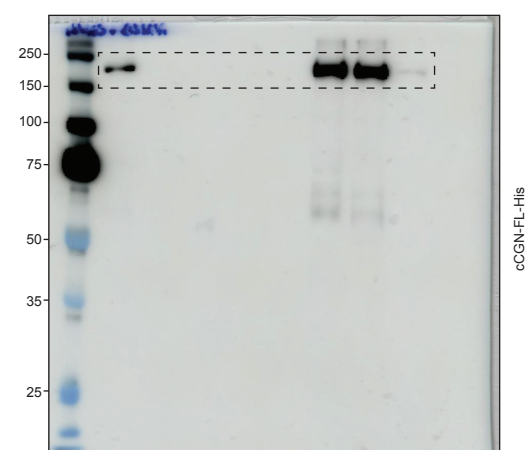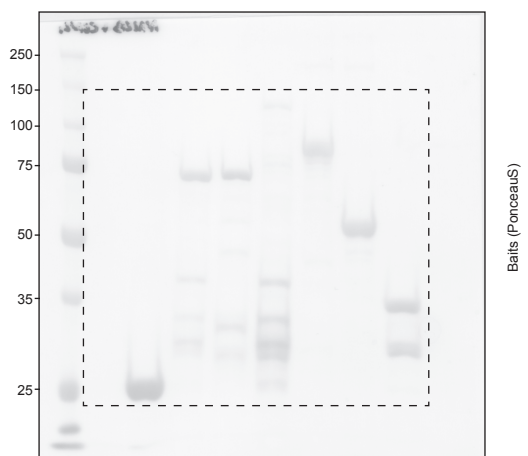

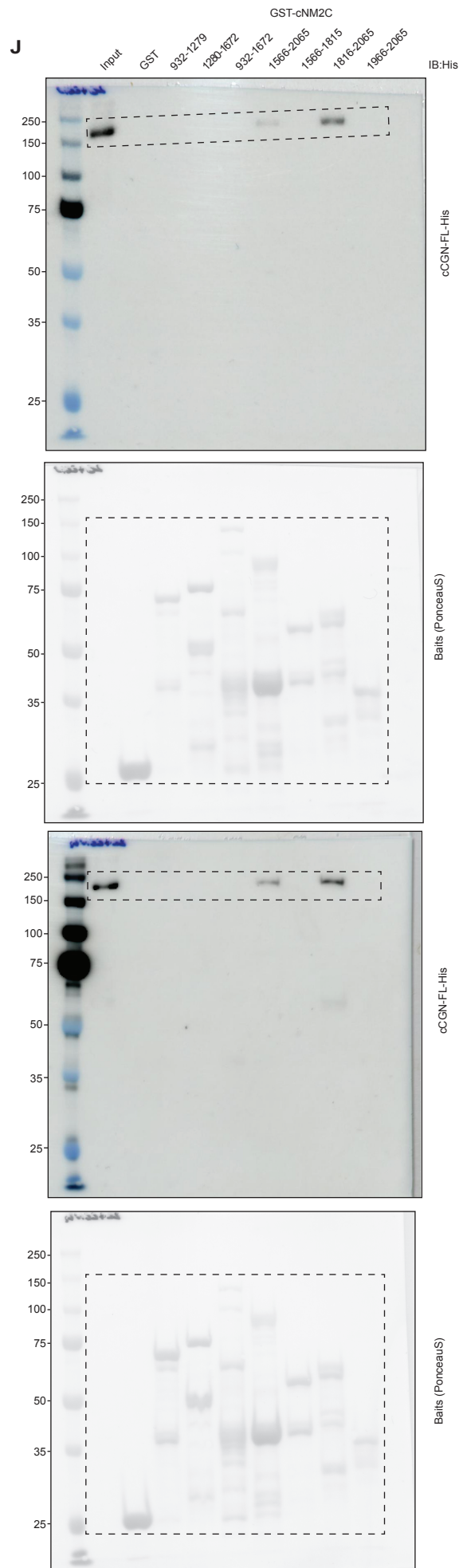

Supplement: SourceData F1 — is the source file for Fig. 1. [file JCB_202208065_SourceDataF1.pdf]

**A**

|                   |   |   |   |   |   |   |
|-------------------|---|---|---|---|---|---|
| hNM2B (1337-1976) | + | + | + | + | - | - |
| hCGN (667-1203)   | - | - | + | + | + | + |
|                   | S | P | S | P | S | P |

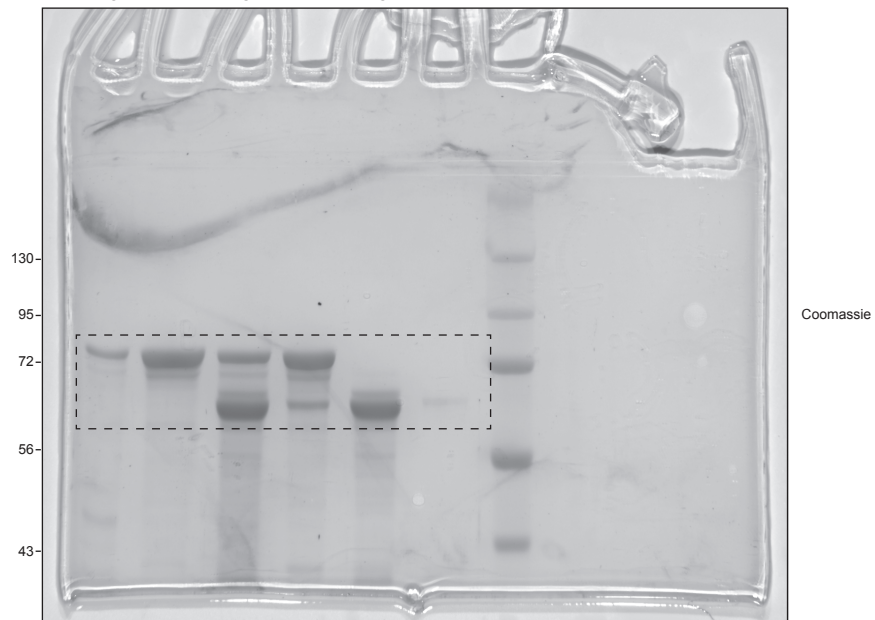

Supplement: SourceData F2 — is the source file for Fig. 2. [file JCB_202208065_SourceDataF2.pdf]

J

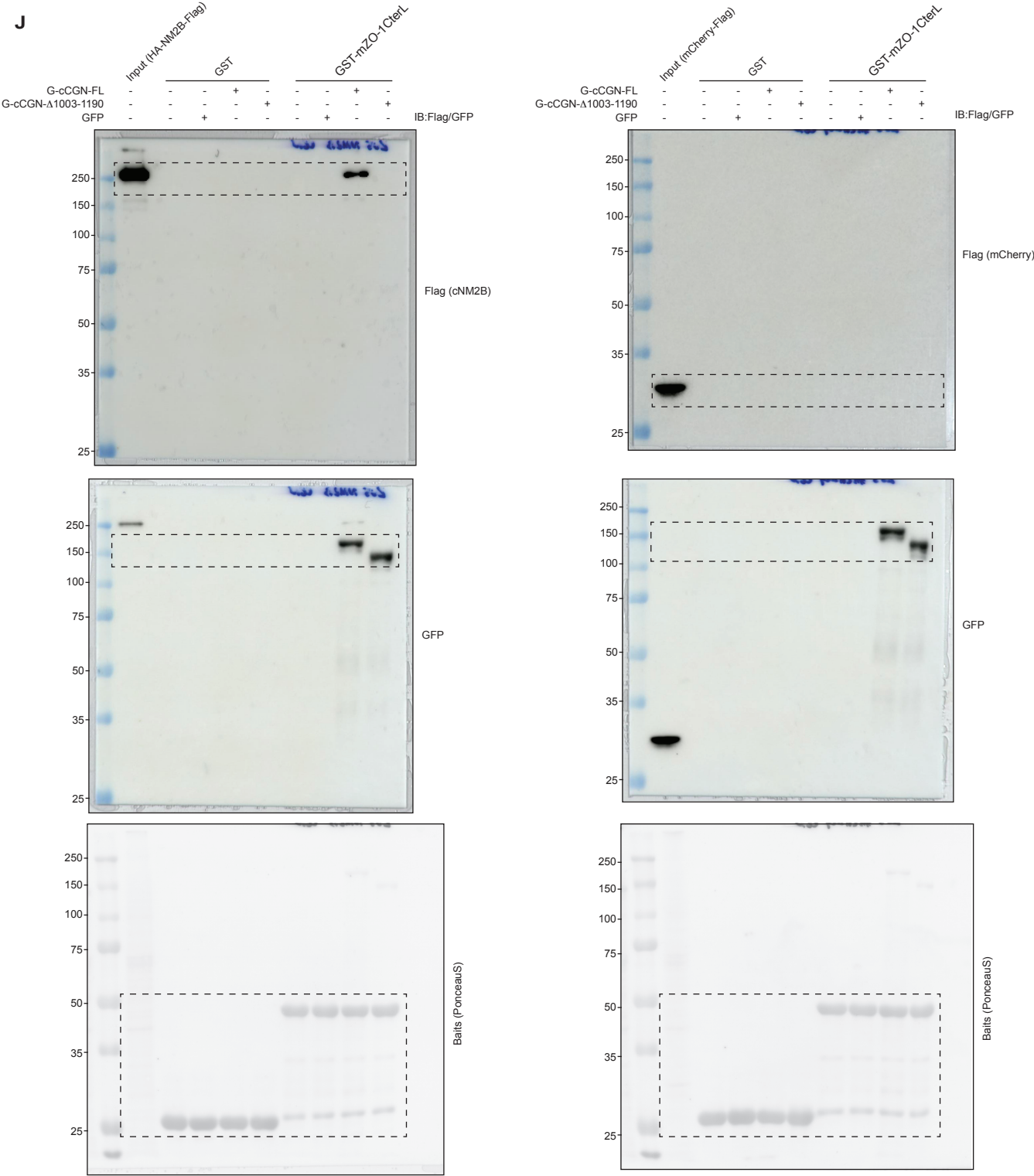

K

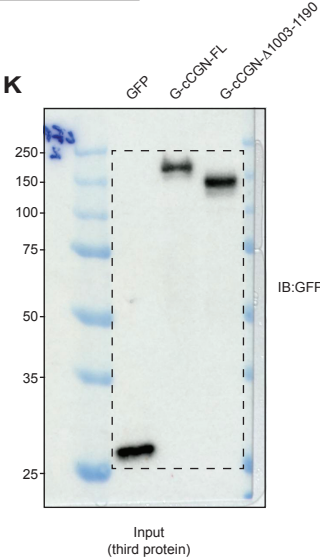

**L**

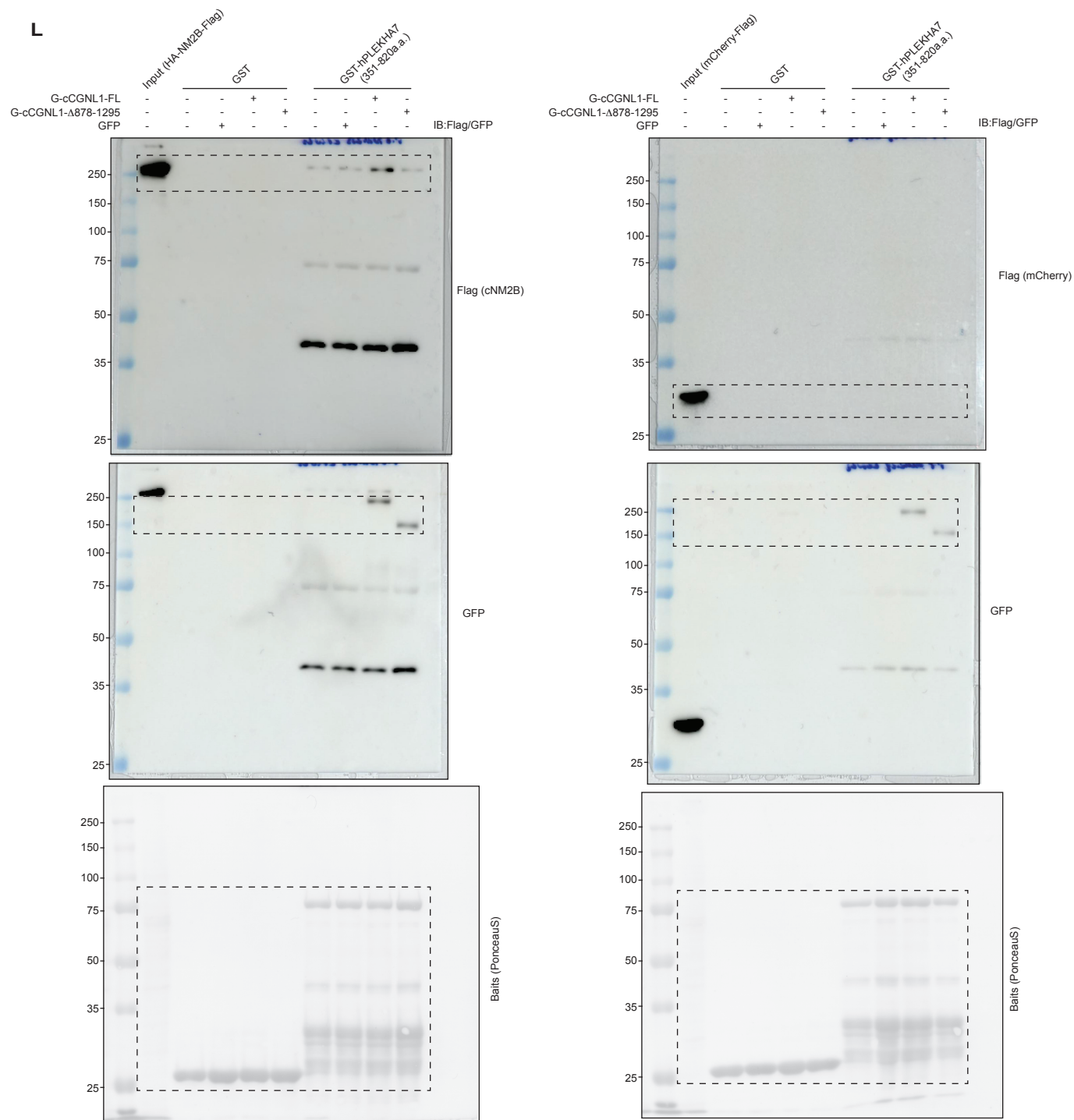

**M**

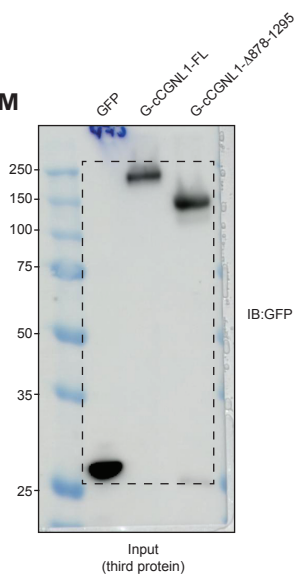

Supplement: SourceData F5 — is the source file for Fig. 5. [file JCB_202208065_SourceDataF5.pdf]

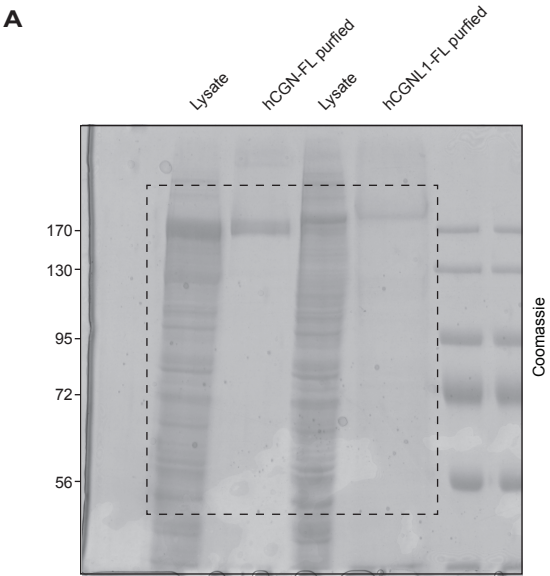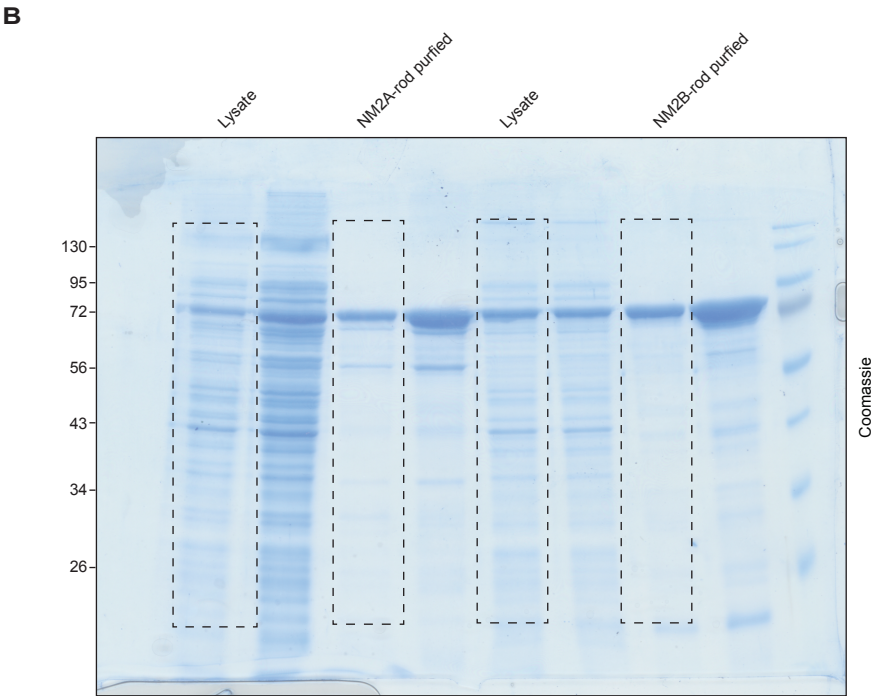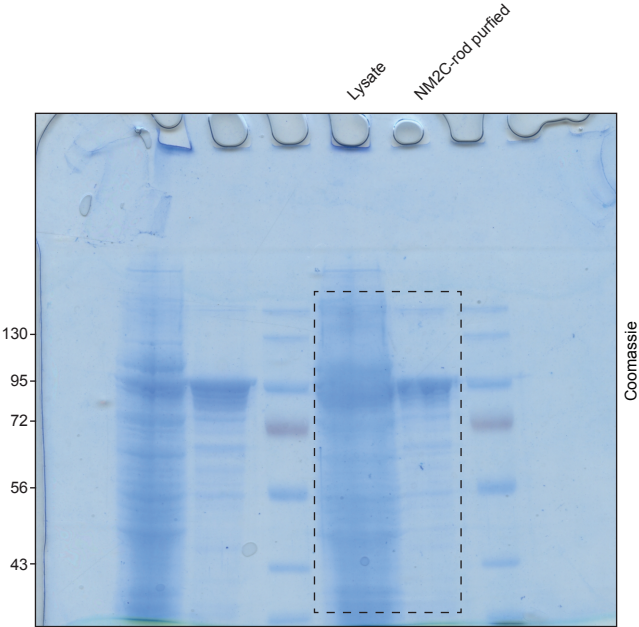

**C**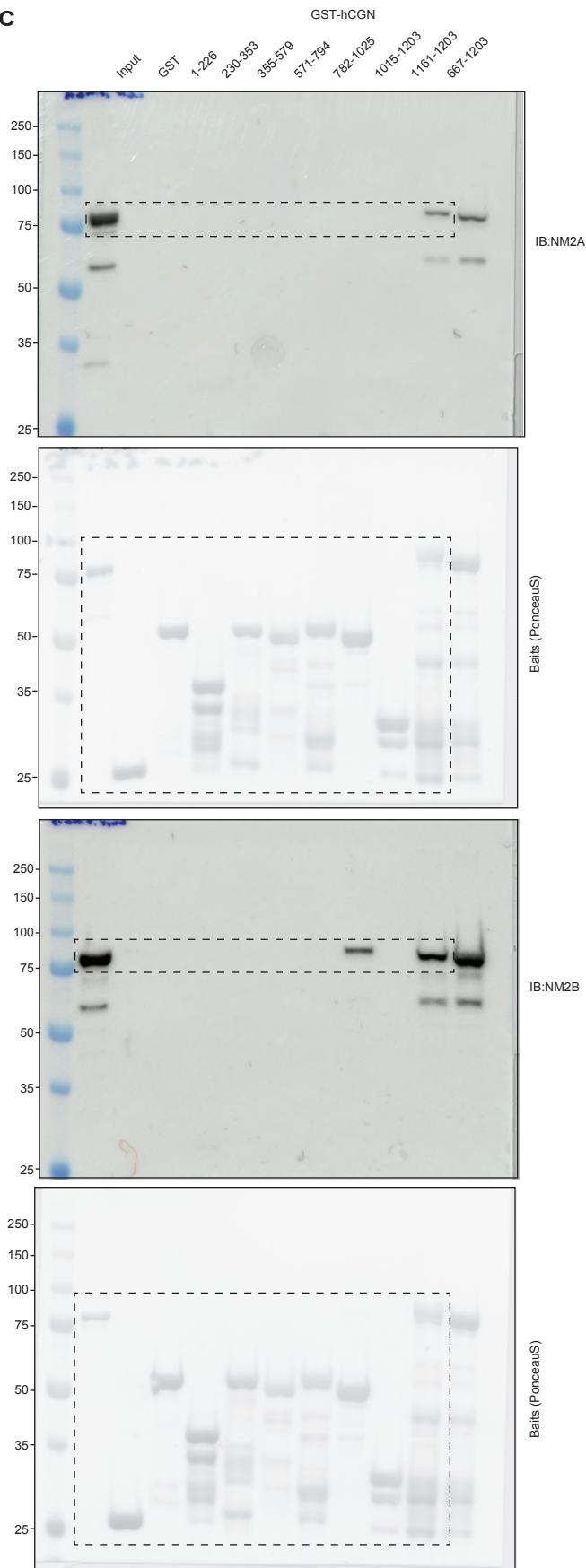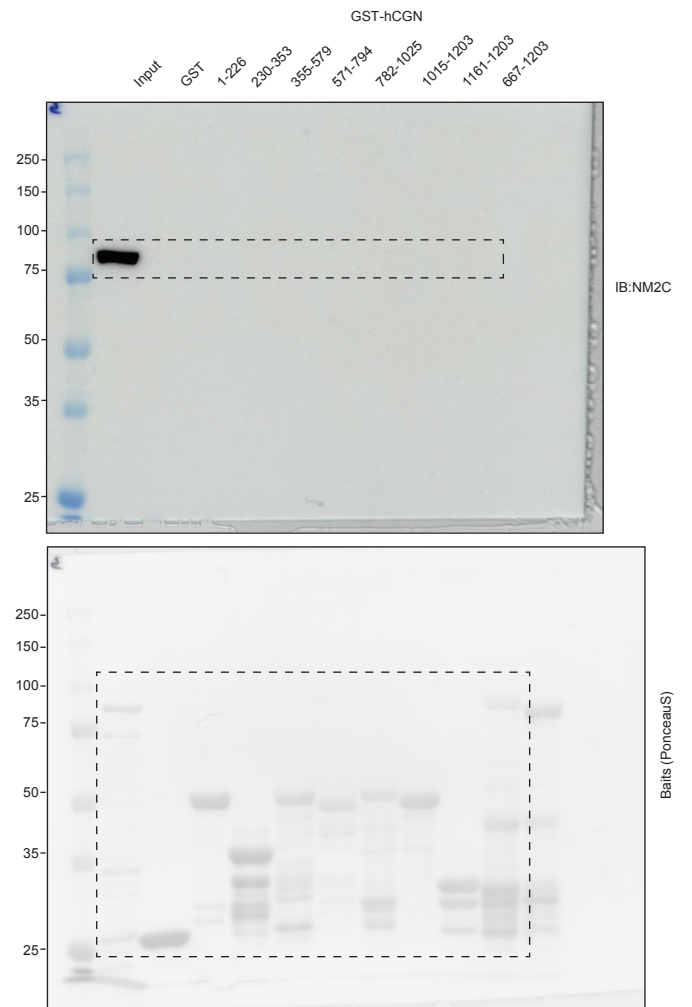

**D**

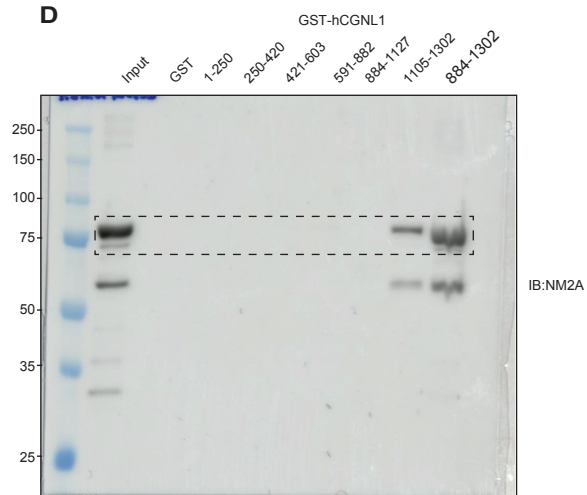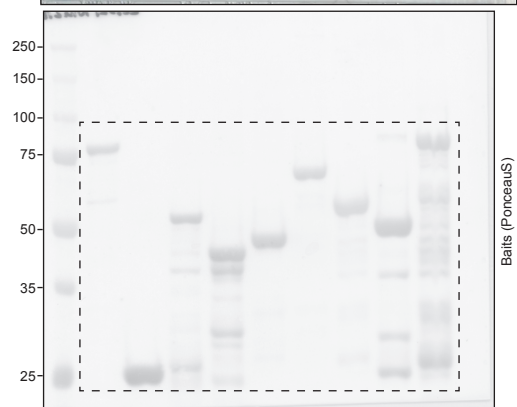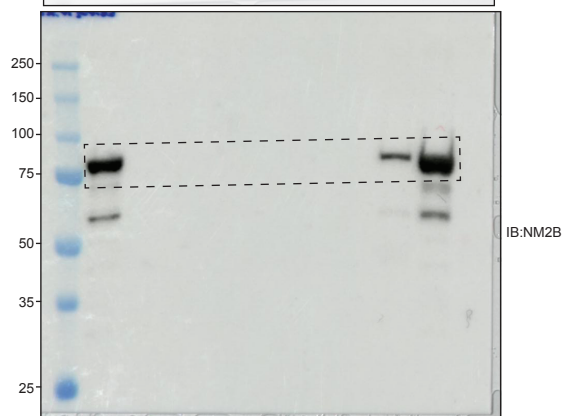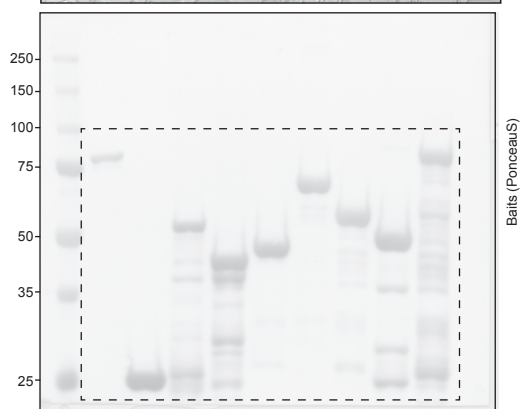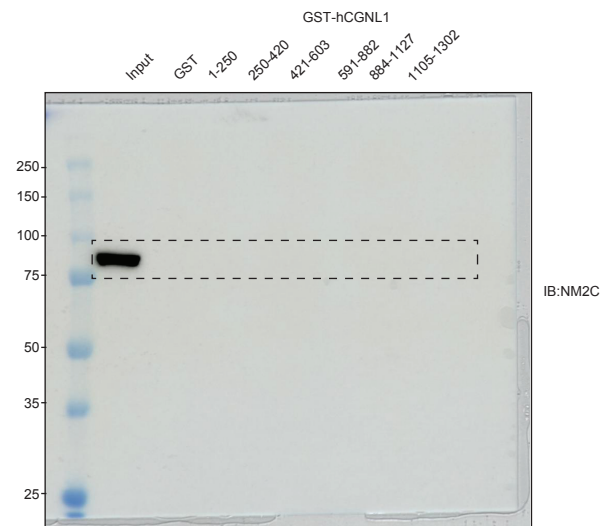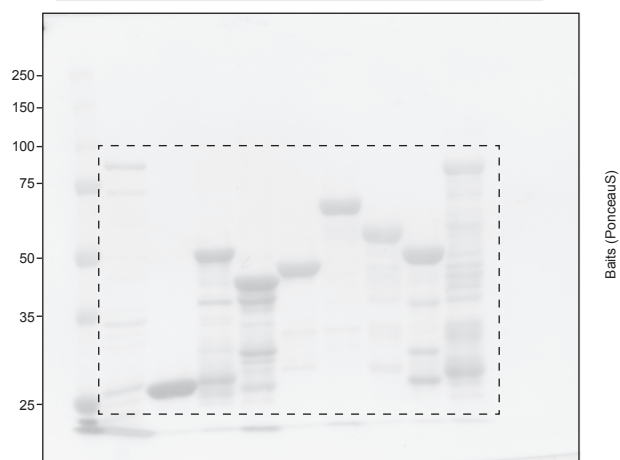

**E**

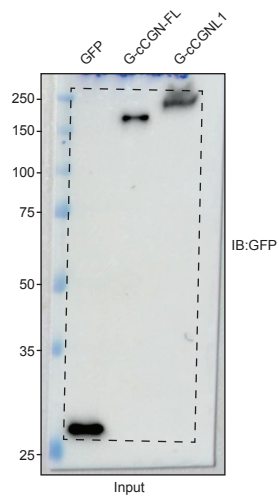

**F**

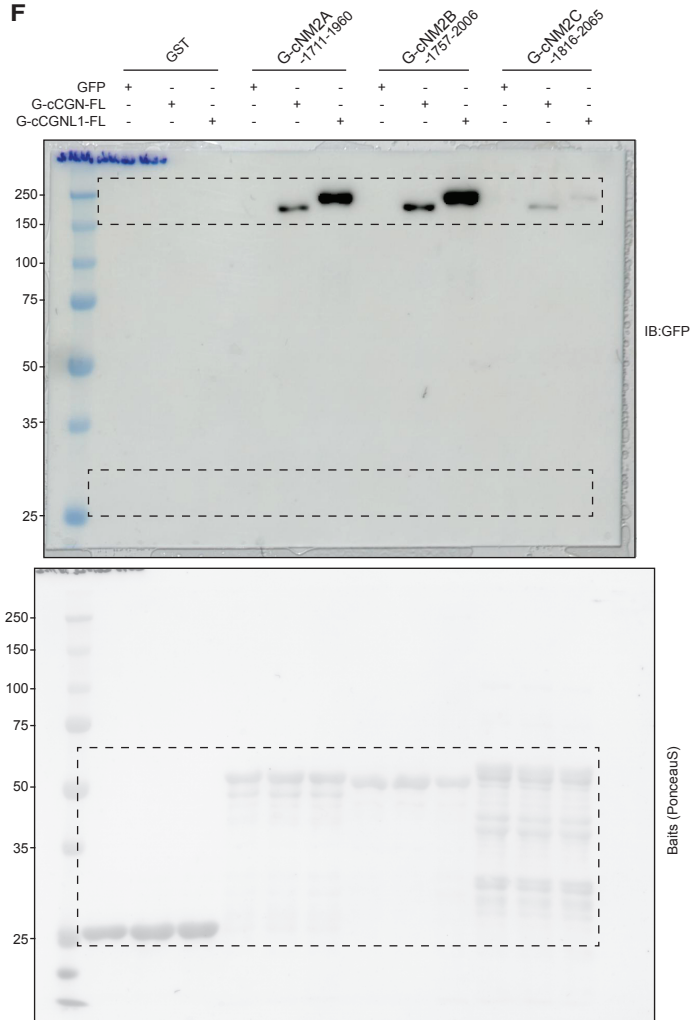

**J**

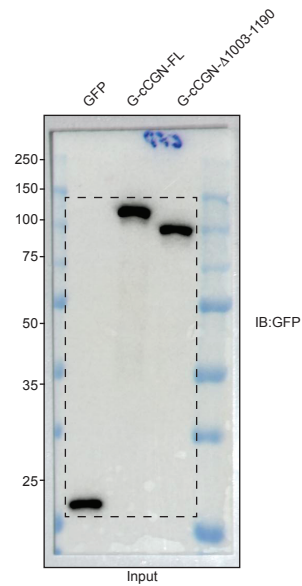

**K**

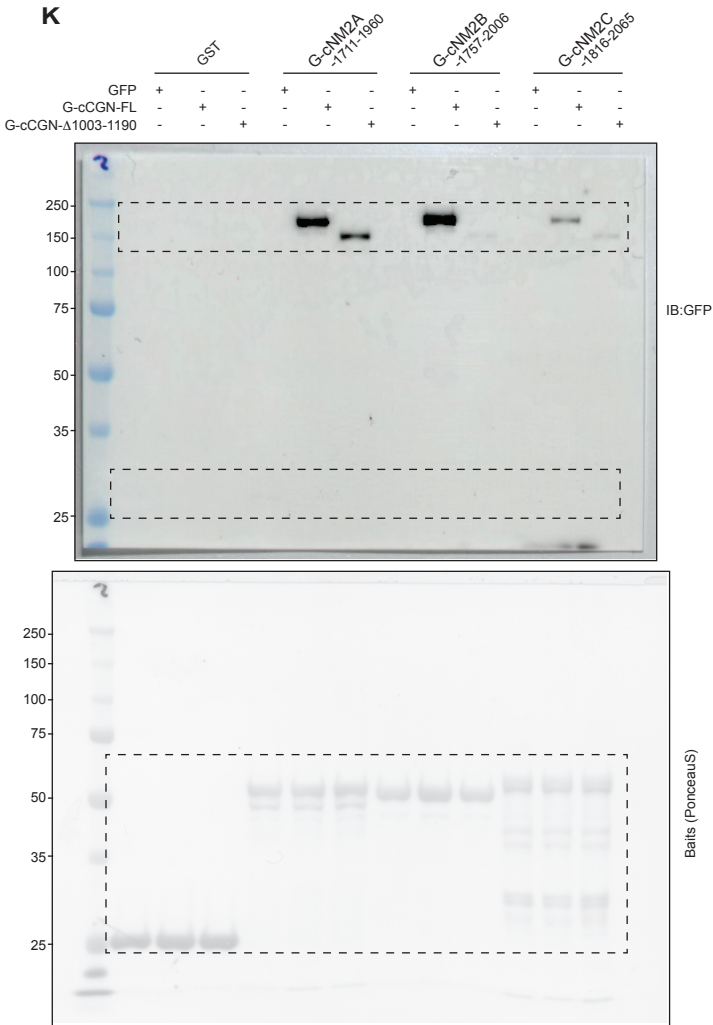

**M**

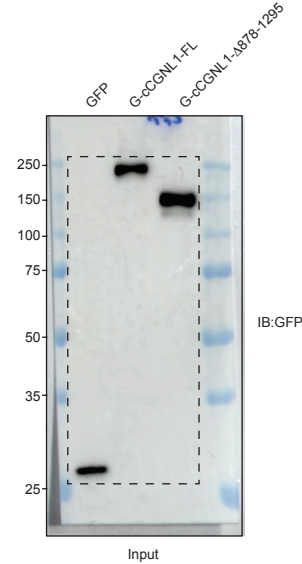

**N**

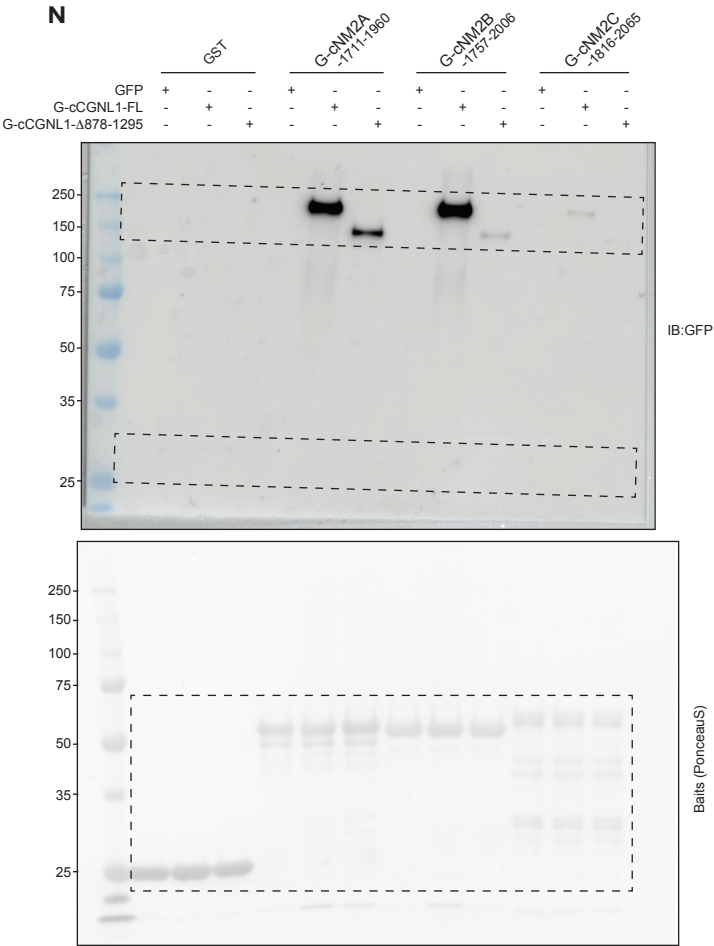

Supplement: SourceData FS1 — is the source file for Fig. S1. [file JCB_202208065_SourceDataFS1.pdf]

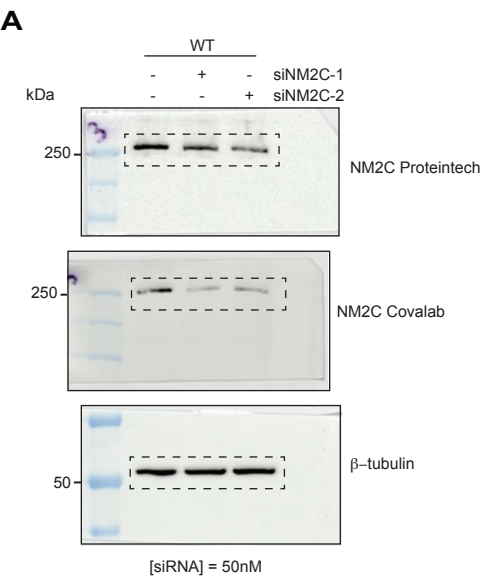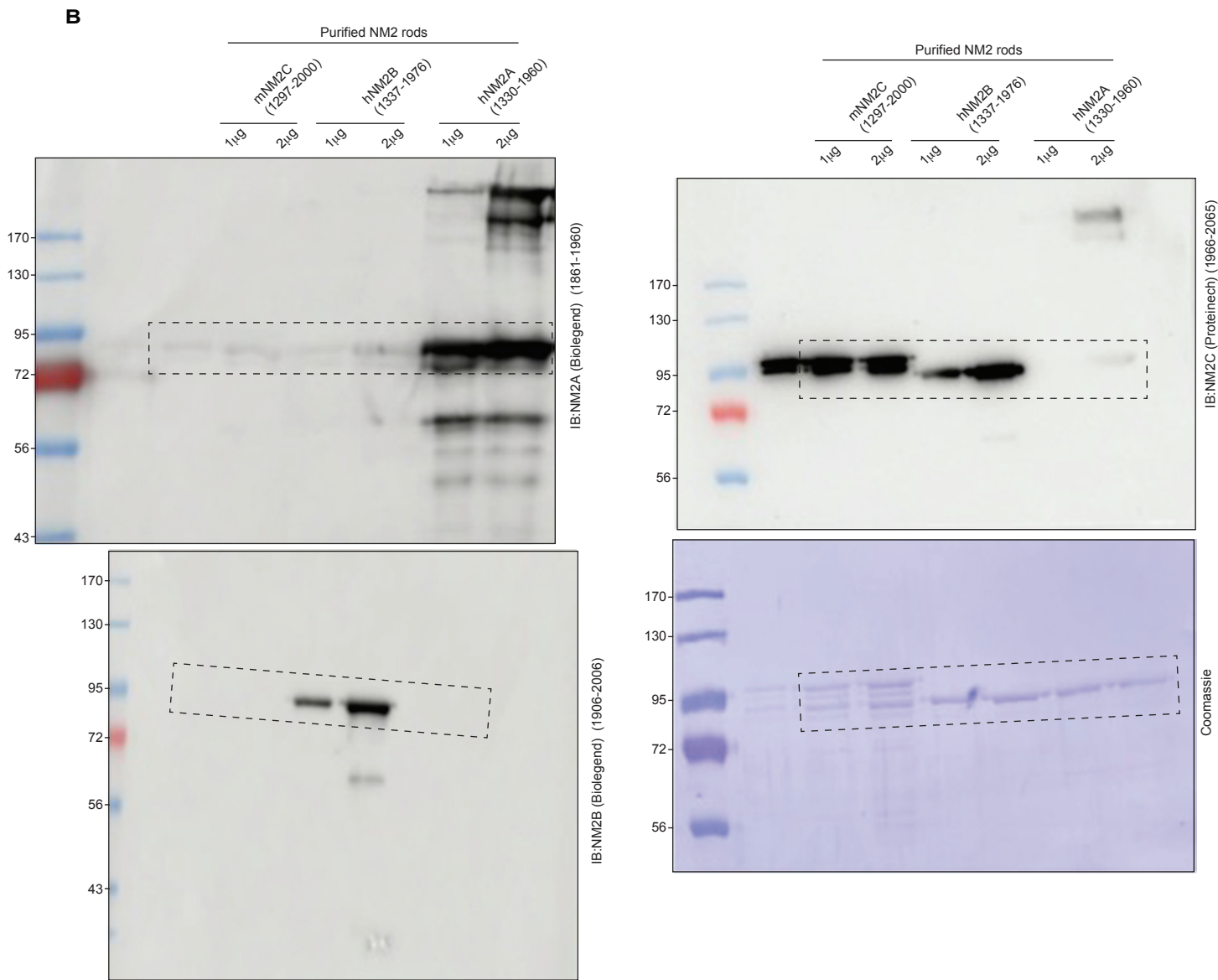

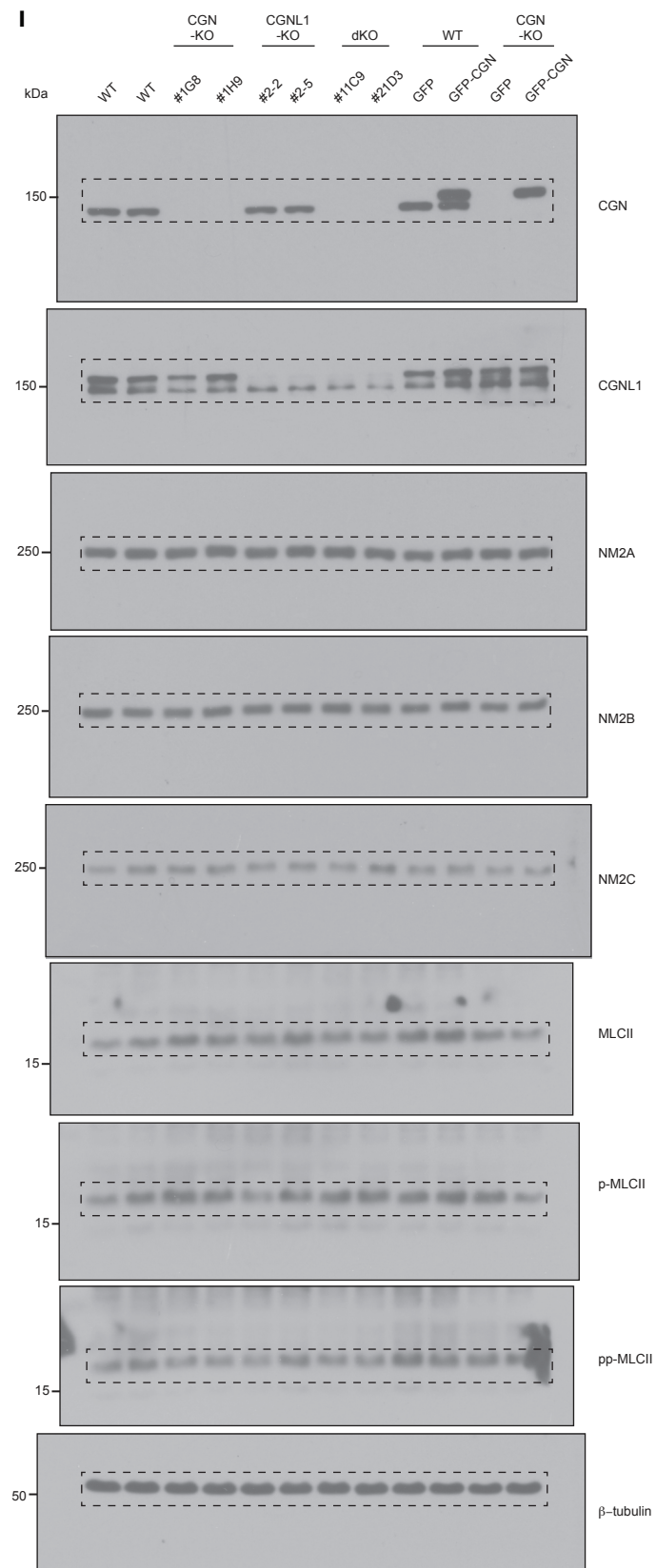

Supplement: SourceData FS2 — is the source file for Fig. S2. [file JCB_202208065_SourceDataFS2.pdf]

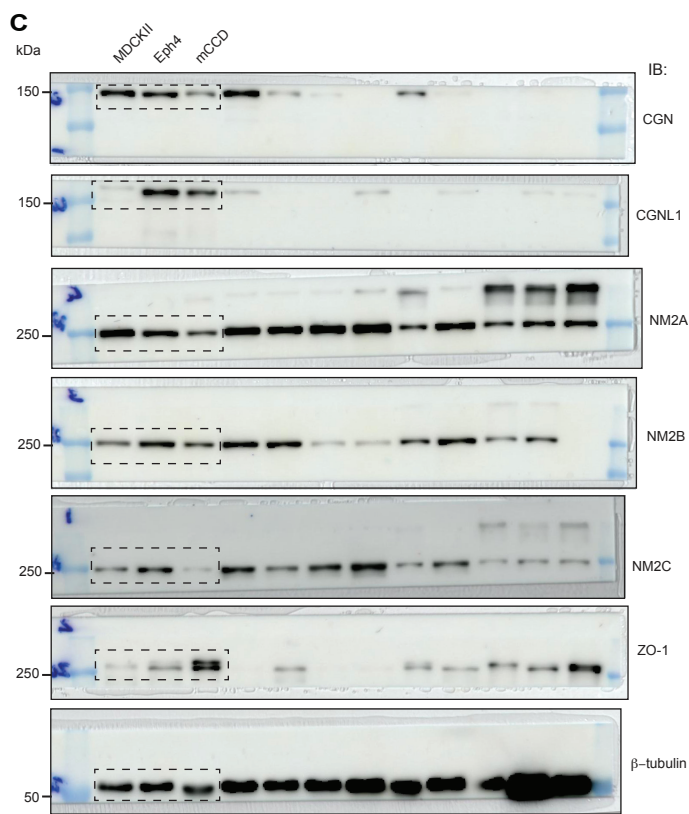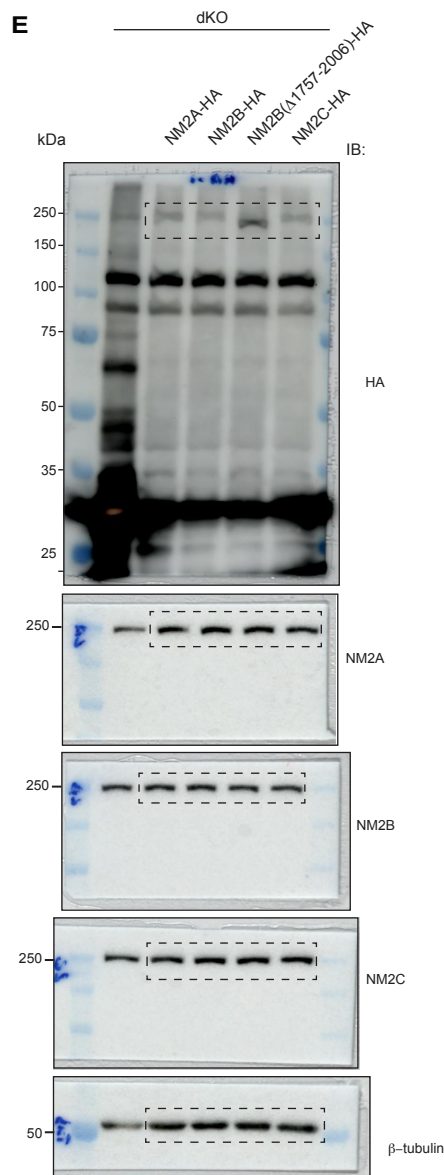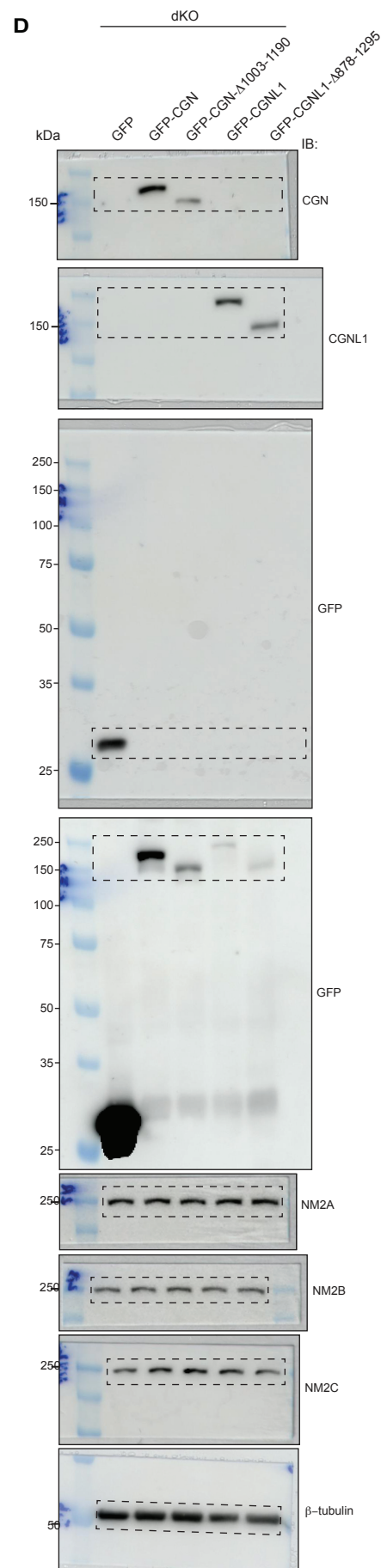

Supplement: SourceData FS3 — is the source file for Fig. S3. [file JCB_202208065_SourceDataFS3.pdf]

SourceDataFS4

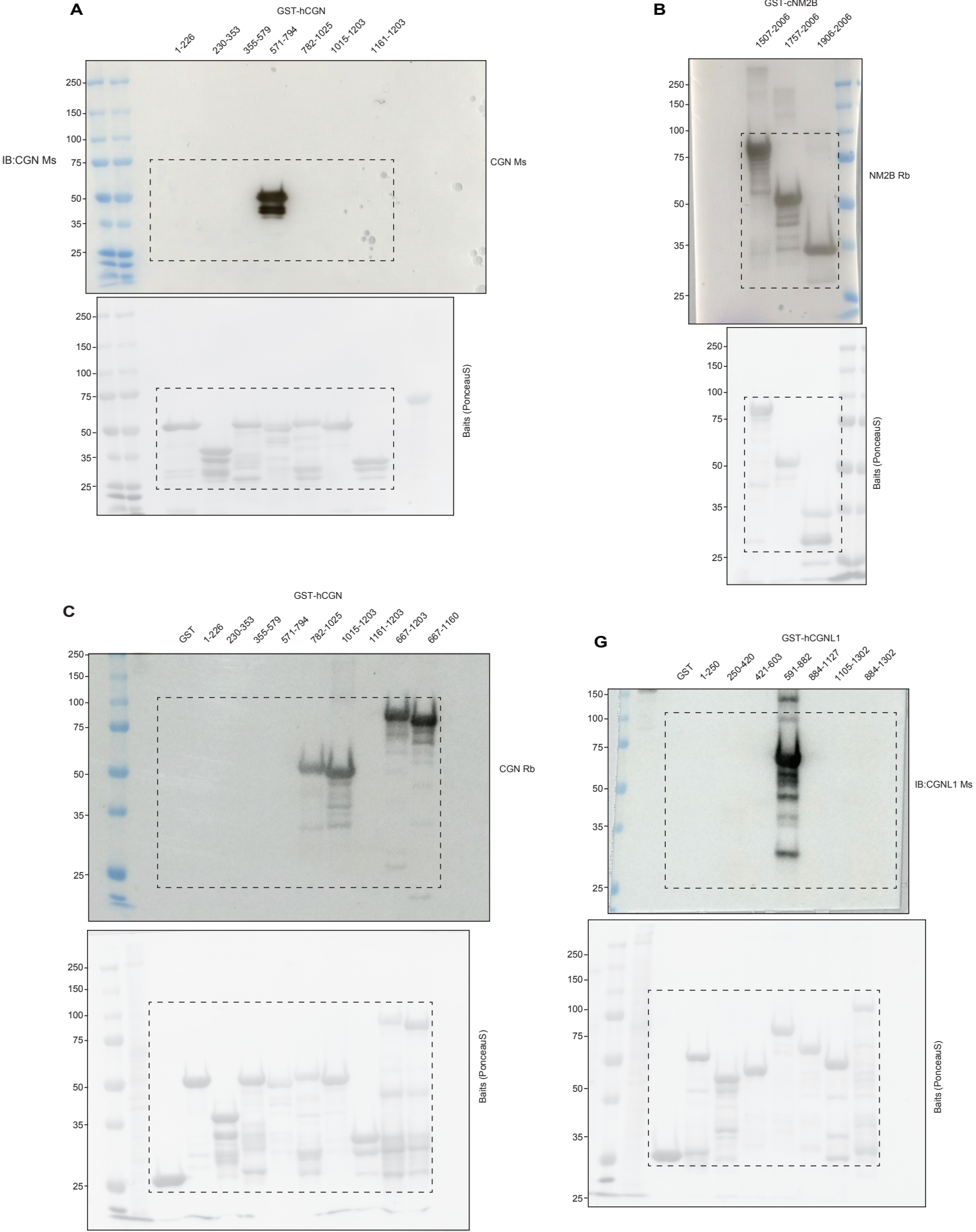

Supplement: SourceData FS4 — is the source file for Fig. S4. [file JCB_202208065_SourceDataFS4.pdf]

A

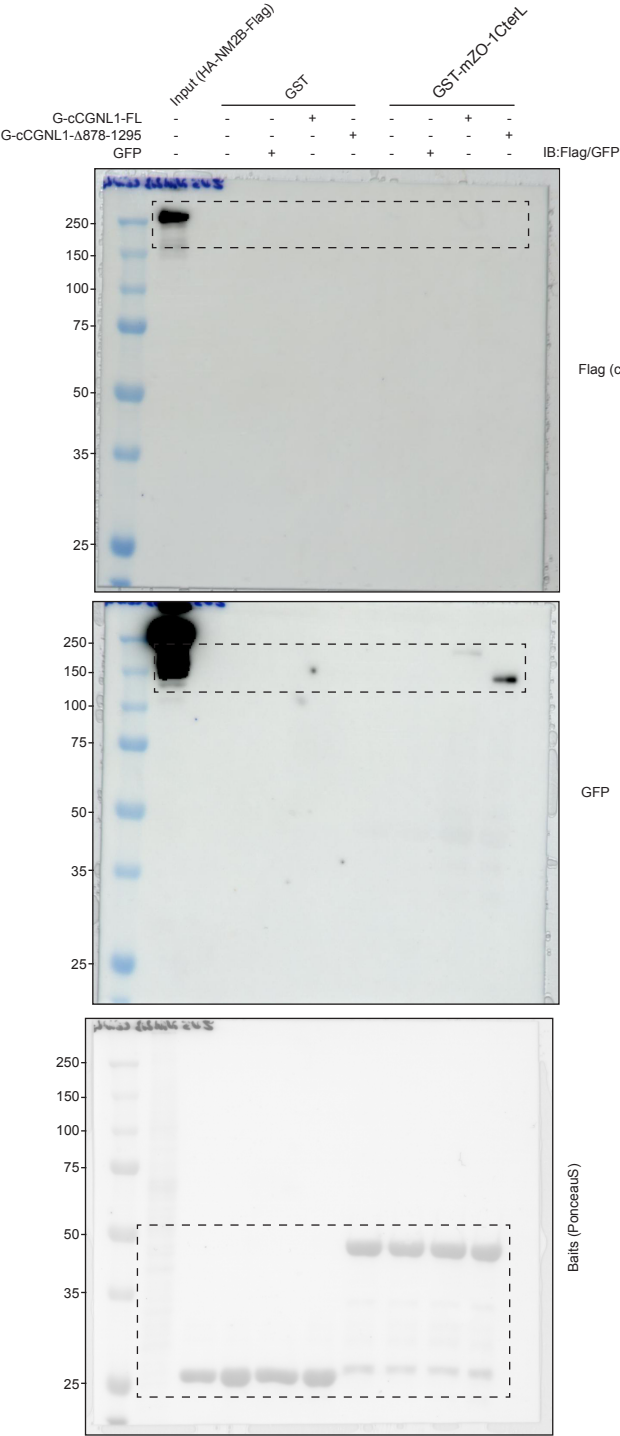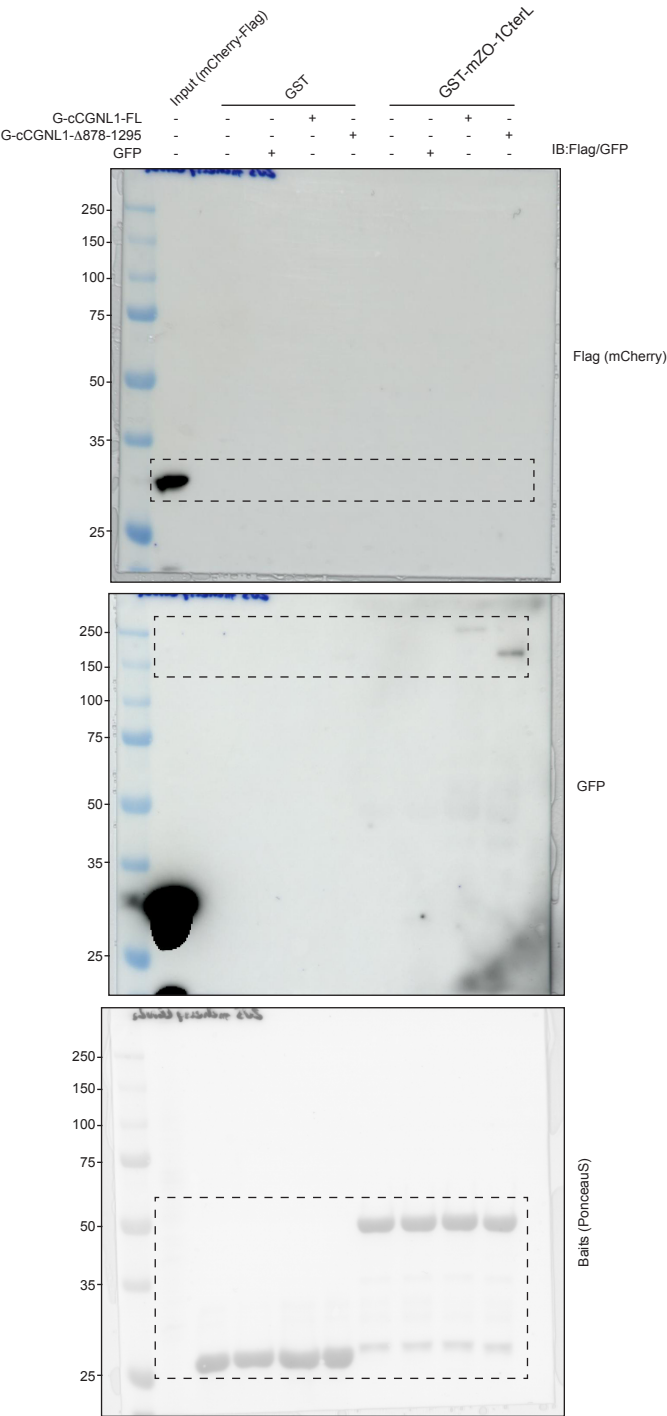

Supplement: SourceData FS5 — is the source file for Fig. S5. [file JCB_202208065_SourceDataFS5.pdf]

**G**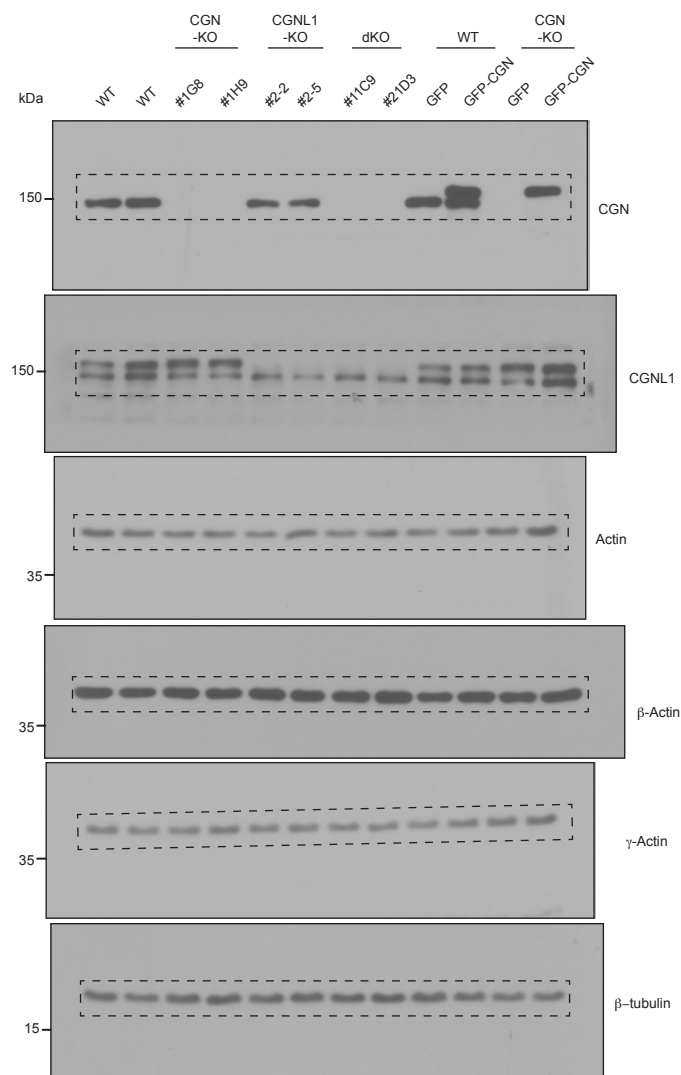

Supplement: SourceData FS6 — is the source file for Fig. S6. [file JCB_202208065_SourceDataFS6.pdf]

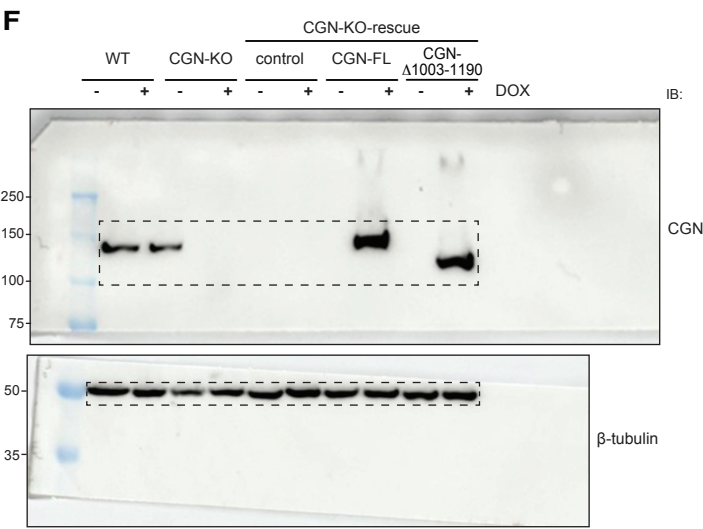

Supplement: SourceData FS7 — is the source file for Fig. S7. [file JCB_202208065_SourceDataFS7.pdf]
